# Sample Sequence Analysis Uncovers Recurrent Horizontal Transfers of Transposable Elements among Grasses

**DOI:** 10.1093/molbev/msab133

**Published:** 2021-05-08

**Authors:** Minkyu Park, Pascal-Antoine Christin, Jeffrey L Bennetzen

**Affiliations:** 1 Department of Genetics, University of Georgia, Athens, GA, USA; 2 Department of Animal and Plant Sciences, University of Sheffield, Sheffield, United Kingdom; 3 State Key Laboratory of Tea Plant Biology and Utilization, Anhui Agricultural University, Hefei, China

**Keywords:** genome evolution, horizontal transfer, *Oryza*, panicoid grasses, Poaceae

## Abstract

Limited genome resources are a bottleneck in the study of horizontal transfer (HT) of DNA in plants. To solve this issue, we tested the usefulness of low-depth sequencing data generated from 19 previously uncharacterized panicoid grasses for HT investigation. We initially searched for horizontally transferred LTR-retrotransposons by comparing the 19 sample sequences to 115 angiosperm genome sequences. Frequent HTs of LTR-retrotransposons were identified solely between panicoids and rice (*Oryza sativa*). We consequently focused on additional *Oryza* species and conducted a nontargeted investigation of HT involving the panicoid genus *Echinochloa*, which showed the most HTs in the first set of analyses. The comparison of nine *Echinochloa* samples and ten *Oryza* species identified recurrent HTs of diverse transposable element (TE) types at different points in *Oryza* history, but no confirmed cases of HT for sequences other than TEs. One case of HT was observed from one *Echinochloa* species into one *Oryza* species with overlapping geographic distributions. Variation among species and data sets highlights difficulties in identifying all HT, but our investigations showed that sample sequence analyses can reveal the importance of HT for the diversification of the TE repertoire of plants.

## Introduction

Evolution results from selection and drift controlling the fate of modifications of the genetic material of organisms. Genetic variants can result from gene or genome duplication (Crow and Wagner 2006), substitutions (Lenski et al. 2003), recombination (Bodmer and Parsons 1963), transposon activity (Kidwell and Lisch 2000), and horizontal transfer (HT) of DNA (Schaack et al. 2010). As more genome sequences become available, we are able to gain new insights into these processes and their impacts. While the importance of HT for some eukaryotes has been clearly established (Keeling and Palmer 2008; Schaack et al. 2010), the phenomenon remains poorly studied, mainly because its existence remained debated until recently (Schaack et al. 2010; Martin 2017; Sibbald et al. 2020).

HT refers to the movement of DNA across mating barriers. The phenomenon has been reported among fungi (Fitzpatrick 2012), insects (Crow and Wagner 2006), and other animals (Thomas et al. 2010), and between kingdoms (Gladyshev et al. 2008; Richards et al. 2009; Hotopp 2011; Mayer et al. 2011). Several lineages of plants have received HTs from viruses, prokaryotes, and nonplant eukaryotes (Yue et al. 2012; Cheng et al. 2019), as well as from other plants (Vallenback et al. 2010; Christin et al. 2012; El Baidouri et al. 2014; Dunning et al. 2019). Previously reported HTs in plants concerned mainly genes and organelle genomes (Dowson et al. 1989; Jain et al. 1999; Bergthorsson et al. 2003; Richardson and Palmer 2007; Stegemann et al. 2012; Xi et al. 2013). Although transposable elements (TEs) are the major components of many plant genomes (Bennetzen 2000; Schnable et al. 2009; Kim et al. 2014), only three studies have focused on their HT among plants (Diao et al. 2006; Roulin et al. 2008; El Baidouri et al. 2014), and have suggested that HT of LTR-retrotransposons in particular is widespread across diverse plant lineages (El Baidouri et al. 2014). However, more studies in plants are required to understand the role of horizontally transferred TEs in plant genome evolution.

In this study, we develop a novel method to investigate HT among plants using low-coverage sample sequences. We focused on panicoid grasses, which have been shown to be involved in HTs (Christin et al. 2012; Dunning et al. 2019). We first look for HT of LTR-retrotransposons from any of 19 panicoid grasses to any of 115 angiosperms with complete genome sequences. We then focus on two genera of grasses for which multiple genome sequences are available to track the dynamics of HT through time. Our study sheds new light on the importance of HT for the diversification of plant genomes.

## Results

### Targeted Investigation of LTR-Retrotransposons Identifies Putative HTs between Panicoideae Grasses and *Oryza sativa*

**Fig. 1.**
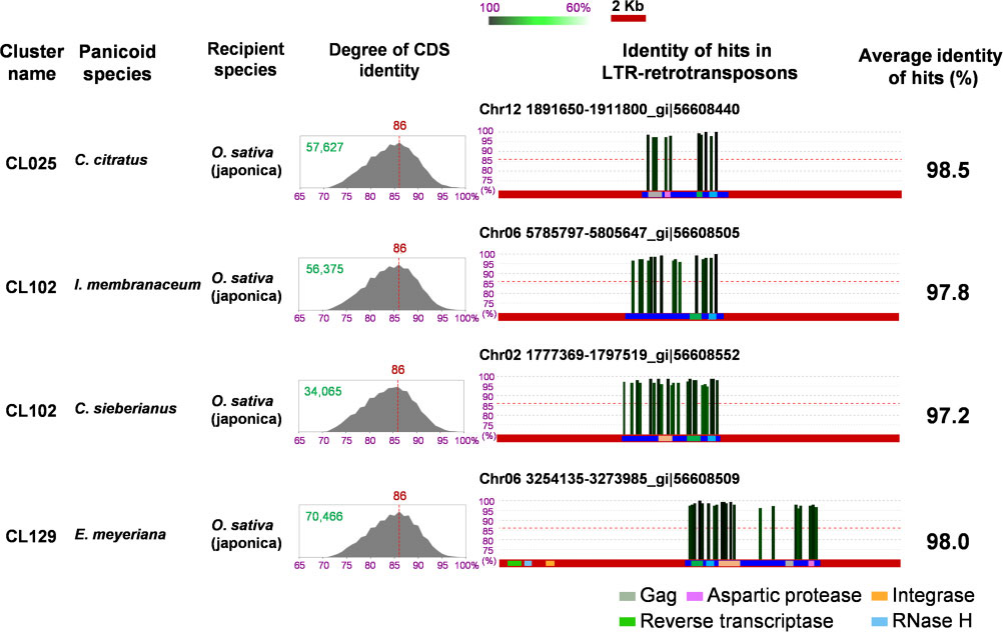
Horizontally transferred LTR-retrotransposons detected between 19 panicoid species and 115 other plant species. The histograms in the “Degree of CDS sequence identity” column indicate the genetic distance between each species pair as shown by the level of CDS sequence homology (the total number of comparisons is shown with a green number on each histogram). The peak point of the histogram is considered as a representation of the degree of divergence from the speciation event and is marked with a vertical dotted red line, with the percent identity at the top. For the four HTs, the identity of the panicoid sample sequence hits to the target genome is shown on the right. Sequences of the recipient genomes are depicted by horizontal red bars and domains of LTR-retrotransposons by gray, pink, orange, green, and light blue boxes that represent gag (GAG), aspartic protease (AP), integrase (IN), reverse transcriptase (RT), and RNase H (RH), respectively. Because of sequence assembly issues or internal rearrangements, some domains are missing (CL102, *Iseilema membranaceum* and CL102, *Cenchrus sieberianus*) or in a nonstandard order (CL129, *Eriochloa meyeriana*). The whole unit of the LTR-retrotransposon is delimited with blue bars, showing that there was no additional high (HT-derived) homology between the panicoid and *Oryza* genomes flanking the precise TE ends themselves. Vertical bars indicate the position and identity of the hits. The height and color intensity of the bars are proportional to the degree of identity. The level of CDS identity indicating the speciation point is depicted by a dotted horizontal red line on each graph.

To search for possible horizontally transferred LTR-retrotransposons, we generated low-depth sample sequences (with ∼2.3× coverages of the expected genomes, given their predicted sizes) from 19 panicoid genomes (supplementary table S1 and fig. S1, Supplementary Material online). Targeted investigation of LTR-retrotransposons was conducted by screening the LTR-retrotransposons of 115 plant species that have reported complete genome sequences (supplementary table S2, Supplementary Material online) with reverse transcriptase (RT) sequences. Initially, the well-conserved YXDD domain of the RT was used in the HMM search. Horizontally transferred LTR-retrotransposons were identified as those with mapped sample sequences with >97% identity. Through this, a total of four cases of possible horizontally transferred LTR-retrotransposons were identified as events into the *O. sativa* genome from a panicoid genome, with an average identity of mapped sample sequences between 97.5% and 98.5% (fig. 1).

To verify the adequacy of the identity threshold, we compared the identity of nuclear gene coding sequence (CDS) between *O. sativa* and each of the four panicoid species that were involved in the putative HT events. The pairwise identity between all four panicoid sample sequences and the CDS of *O. sativa* peaked at 86% (fig. 1). Only 2.6% of the sample sequences had >97% identity with the CDS of *O. sativa*. Therefore, the >97% threshold is high enough for the identification of horizontally transferred LTR-retrotransposons.

### HTs of LTR-Retrotransposons between the Panicoid Grasses and *Oryza*

**Fig. 2.**
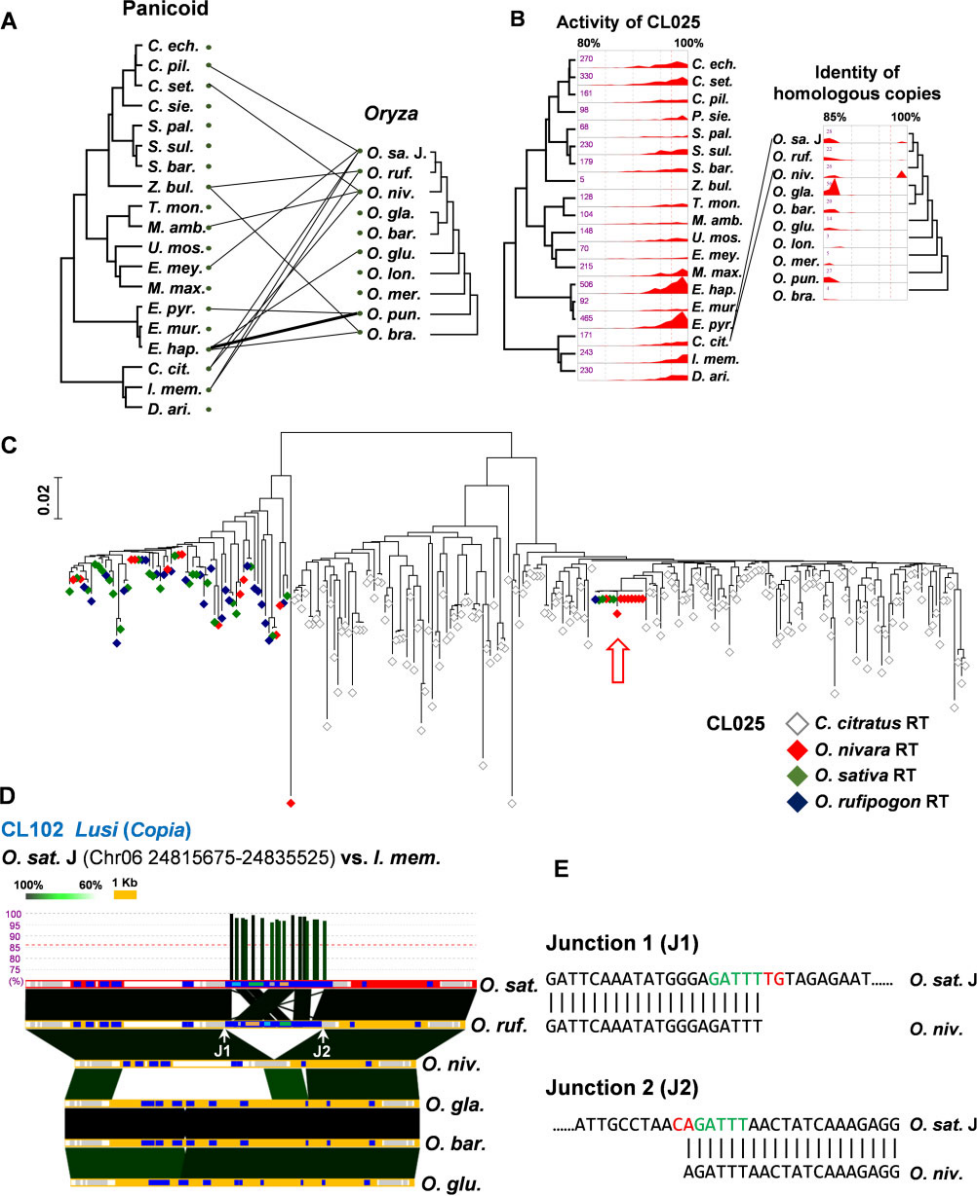
HTs of LTR-retrotransposons between the studied panicoids and ten *Oryza* species. (*A*) Pairs of species involved in horizontal transfers of LTR-retrotransposons are connected. The thick line represents two HT events. Abbreviations for panicoids; *C. ech.*, *Cenchrus echinatus*; *C. pil.*, *Cenchrus pilosus*; *C. set*.: *Cenchrus setigerus*; *C. sie*., *Cenchrus sieberianus*; *S. pal*.: *Setaria cf. palmifolia*; *S. sul*.; *Setaria cf. sulcata*; *S. bar*., *Setaria barbata*; *Z. bul*., *Zuloagaea bulbosa*; *T. mon*., *Tricholaena monachne*; *M. amb*., *Melinis ambigua*; *U. mos*., *Urochloa mosambicensis*; *E. mey*., *Eriochloa meyeriana*; *M. max*., *Megathyrsus maximum*; *E. pyr*., *Echinochloa pyramidalis*; *E. mur*., *Echinochloa muricata*; *E. hap*., *Echinochloa haploclada*; *C. cit*., *Cymbopogon citratus*; *I. mem*., *Iseilema membranaceum*; and *D. ari*., *Dichanthium aristatum*. Abbreviations of *Oryza* species; *O. sa*. J., *O. sativa* Japonica; *O. ruf*., *O. rufipogon*; *O. niv*., *O. nivara*; *O. gla*., *O. glaberrima*; *O. bar*., *O. barthii*; *O. glu*., *O. glumaepatula*; *O. lon*., *O. longistaminata*; *O. mer*., *O. meridionalis*; *O. pun*., *O. punctata*; and *O. bra*., *O. brachyantha*. (*B*) For an HT of LTR-retrotransposons between *C. citratus* and *Oryza* species, the relative activity history of LTR-retrotransposons represented by RTs in CL025 is shown in the left panel. The identity of homologs of the horizontally transferred LTR-retrotransposons in the ten *Oryza* species is presented in the right panel. Homologs with >97% identity are observed in *O. sativa*, *O. rufipogon*, and *O. nivara*. Pairs of species involved in HT events identified by our screening are connected. (*C*) This phylogenetic tree was generated for LTR-retrotransposons of *C. citratus* (empty gray diamonds) and three closely related *Oryza* species; *O. nivara* (red diamonds), *O. sativa* (green diamonds), and *O. rufipogon* (blue diamonds). The red arrow points to the horizontally transferred LTR-retrotransposons related to CL025. (*D*) The genomic region containing an LTR-retrotransposon horizontally transferred from *I. membranaceum* is compared among collinear regions of different *Oryza* species. The vertical bars at the top show the positions and identities of sequences of *I. membranaceum* mapped to the *O. sativa* genome, in red. White and gray boxes in each horizontal bar indicate exons and introns of annotated genes, respectively. Blue boxes indicate annotated LTR-retrotransposons. Domains of LTR-retrotransposons are depicted by gray, pink, orange, green, and light blue boxes, representing gag, aspartic protease, integrase, reverse transcriptase, and RNase H, respectively. Similar regions between species are connected, with colors that indicate the degree of identity. (*E*) The sequence alignments corresponding to the two junctions of CL102 are shown. The “TG……CA” motif of LTR sequences and target site duplications are marked with red and green letters, respectively.

Because all four possible horizontally transferred LTR-retrotransposons were identified in *O. sativa* among the 115 plant species, subsequent analyses focused on the genus *Oryza*. Using ten whole-genome sequences of different *Oryza* species (Goff et al. 2002; Stein et al. 2018), we identified 15 LTR-retrotransposons with high similarity (>97% identity) between the studied panicoid grasses and *Oryza* species (fig. 2*A*, table 1, and supplementary fig. S2, Supplementary Material online).

**Table 1.** Detailed Information Regarding Horizontally Transferred LTR-Retrotransposons.

HT Event	Donor	Recipient	Cluster	Average Identity (%)	Classification by Maize Repeat Database	Classification by GyDB
1	*Echinochloa haploclada*	*Oryza punctata*	CL010	98.7	*CRM1/Gypsy*	*CRM*
2	*Zuloagaea bulbosa*	*Oryza brachyantha*	CL010	98.9		
3	*Melinis ambiigua*	*Oryza nivara*	CL129	97.8	*Wihov/Gypsy*	
4	*Zuloagaea bulbosa*	*Oryza rufipogon*	CL129	99.1		
5	*Eriochloa meyeriana*	*Oryza sativa*	CL129	98.6		
6	*Echinochloa haploclada*	*Oryza brachyantha*	CL212	99.3	*Guhis/Gypsy*	
7	*Cymbopogon citratus*	*Oryza sativa*	CL025	99.3	Unknown/*Copia*	*Tork*
		*Oryza nivara*	CL025	99.3		
8	*Echinochloa haploclada*	*Oryza glumaepatula*	CL112	97.3	*Debeh/Copia*	
9	*Echinochloa pyramidalis*	*Oryza punctata*	CL148	98.4	*Dounil/Copia*	
	*Echinochloa haploclada*		CL148	98.1		
10	*Iseilema membranaceum*	*Oryza rufipogon*	CL102	97	*Lusi/Copia*	*Retrofit*
		*Oryza sativa*	CL102	97.8		
11	*Cenchrus pilosus*	*Oryza sativa*	CL102	97.3		
12	*Cenchrus setigerus*	*Oryza nivara*	CL329	97.1	*Hera/Copia*	

The RT sequences used in the screening of horizontally transferred LTR-retrotransposons were clustered for subsequent analyses and a total of 368 RT clusters were obtained. To investigate which of these 368 might be horizontally transferred, we constructed phylogenetic trees with the RT sequences of the clusters containing possible horizontally transferred LTR-retrotransposons and their homologs in the *Oryza* species. For 14 of the 15 putative horizontally transferred LTR-retrotransposons, phylogenetic analyses confirmed that some *Oryza* RT sequences are nested among those of the panicoid species (supplementary fig. S3, Supplementary Material online), as expected with HT. For the last candidate (CL329), only five RT sequences were present in the cluster, making the phylogenetic tree uninformative. In all phylogenetic trees, the *Oryza* RT sequences were nested among those from panicoid grasses, indicating that the *Oryza* lineage was the recipient of the HT. In two cases, the HTs of two different *Oryza* species were placed together in a single phylogenetic tree (CL025, supplementary fig. S3*G*, Supplementary Material online; CL 102, supplementary fig. S3*J*, Supplementary Material online), while in one case sequences detected as HTs in comparisons between two different panicoid (*Echinochloa*) species and the same *Oryza* species (*Oryza punctata*) were placed in a single tree containing all three species (CL148; supplementary fig. S3*I*, Supplementary Material online), leading to only 11 unique phylogenetic trees (supplementary fig. S3, Supplementary Material online). Considering CL329 as well, the 15 horizontally transferred LTR-retrotransposon candidates would represent 12 HT events (table 1).

To examine whether the presence of horizontally acquired sequences in multiple *Oryza* species results from a single transfer in their common ancestor, we first analyzed the HT case of CL025, which was also identified in the first search (fig. 1). Some CL025 elements were identified with high identity between *Cymbopogon citratus* and both of the closely-related species *O. sativa* and *Oryza nivara* (fig. 2*B*). Although it was not detected in our HT search, potentially because of a low number of mapped reads, one homolog of the sequence was also identified in *Oryza rufipogon*. The phylogenetic analysis of CL025 showed that sequences from the three *Oryza* species grouped together and nested within *C. citratus*, as expected following HT to the common ancestor of the three *Oryza* species (fig. 2*C*). We performed similar analyses with all of the other HT candidates, and the phylogenetic analysis demonstrated that CL102 was likely transferred from *Iseilema membranaceum* or its relative to the common ancestor of *O. sativa* and *O. rufipogon* (supplementary fig. S3*J*, Supplementary Material online). Alternatively, CL102 may have arrived in one of these two lineages, and then have been introgressed into the other in one of their rare crosses. In the case of CL148, a HT was detected between *O. punctata* and each of *Echinochloa pyramidalis* and *Echinochloa haploclada* (supplementary fig. S2, Supplementary Material online, CL148), but the phylogenetic tree indicates that sequences *O. punctata* are nested in a clade composed of the two *Echinochloa* sequences (supplementary fig. S3*I*, Supplementary Material online). This may indicate that the TE was transferred to the *O. punctata* lineage from a close relative of the species or of the common ancestor of *E. pyramidalis* and *E. haploclada*.

The HT of an LTR-retrotransposon would appear as a new insertion when compared with the collinear regions of other *Oryza* genomes that lack the HT. To test this prediction, comparative analyses of genomic fragments examined all putative HTs. Seven out of twelve putative HT events are located in highly repetitive regions, leading to potential assembly problems (table 1, HT events # 1, 2, 3, 5, 6, 8, and 9). For the other five candidates, orthologous sequences to the regions harboring the horizontally transferred LTR-retrotransposon were isolated from different *Oryza* species. The CL102 of *O. sativa* has >97% identity to an *I. membranaceum* LTR-retrotransposon, and the same LTR-retrotransposon is identified in the collinear region of *O. rufipogon*, suggesting that the sequence was transferred to this genomic region in the common ancestor of the two *Oryza* species. The horizontally transferred LTR-retrotransposon was absent in the collinear regions of the other *Oryza* species (fig. 2*D*). The sequences at the junctions of the insertion site contained the expected terminal “TG……CA” motifs, for both LTR sequences, and target site duplications were also observed (fig. 2*E*, green letters in the right panel). This suggests that the LTR-retrotransposon was horizontally transferred as a unit that can integrate into the host genome. Similar patterns are observed for the HT of CL025, the other HT of CL102 (*O. sat.* – *C. pil.*), the HTs of CL129, and CL329 (supplementary fig. S4, Supplementary Material online). To verify the junctions, we aligned the raw reads of *O. sativa* (SRA ID: ERX3148290) to the relevant regions. In the case of CL102 (*O. sat.* – *C. pil.*), we identified reads spanning native and horizontally transferred sequences (supplementary fig. S5, Supplementary Material online), confirming that the foreign elements are integrated in the genome of *O. sativa*. In all cases, the comparative analyses of collinear sequences strongly support the HT cases identified by the investigation of sequence similarities.

Besides providing information about shared HT events, collinearity analyses can also reveal intragenomic dynamics. In the phylogenetic tree of CL025, four *O. sativa* and nine *O. nivara* RTs were identified in the *C. citratus* RT clade (fig. 2*C*). This suggests the LTR-retrotransposon of *C. citratus* amplified in the *O. sativa* and *O. nivara* genomes after the HT event. The homologs of the horizontally transferred LTR-retrotransposon were identified from different locations of the *O. sativa* genome and the identity between the homologs ranged from 98.9% to 100%, confirming post-HT amplification (supplementary fig. S6, Supplementary Material online).

### Nontargeted Investigation of HTs between *Echinochloa* and *Oryza*

Out of 12 HT events of LTR-retrotransposons detected in *Oryza* species, four were received from the *Echinochloa* genus, which was thus selected for nontargeted investigations to allow the detection of other types of horizontally transferred fragments. Six additional *Echinochloa* species were sample sequenced (supplementary table S3, Supplementary Material online), leading to a total of nine *Echinochloa* and ten *Oryza* species in these analyses (supplementary fig. S7, Supplementary Material online).

**Fig. 3.**
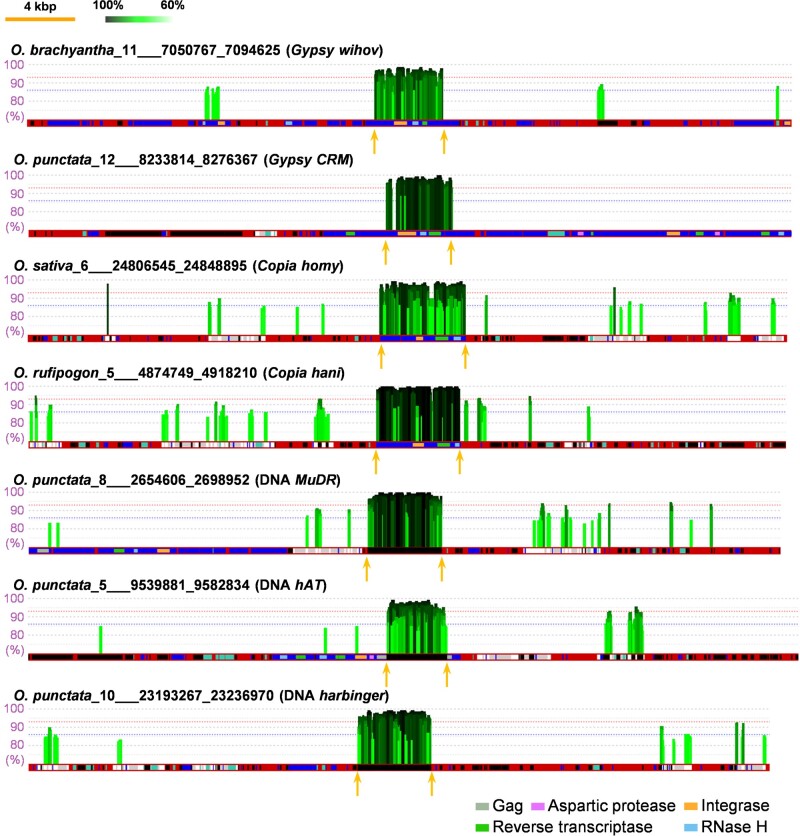
Horizontal transfers detected by nontargeted screens. Mapping results are shown for seven HT cases. The horizontal red bars represent *Oryza* target sequences harboring the HT elements, with vertical bars corresponding to mapped reads from *Echinochloa* species. The height and color intensity indicate the degree of homology of the mapped sequence. Black boxes in the red bars represent DNA transposons. Genes, LTR-retrotransposons, and domains of the LTR-retrotransposons are shown in the same way as in figure 2*D*. Termini of the horizontally transferred LTR-retrotransposons are indicated by arrows.

**Fig. 4.**
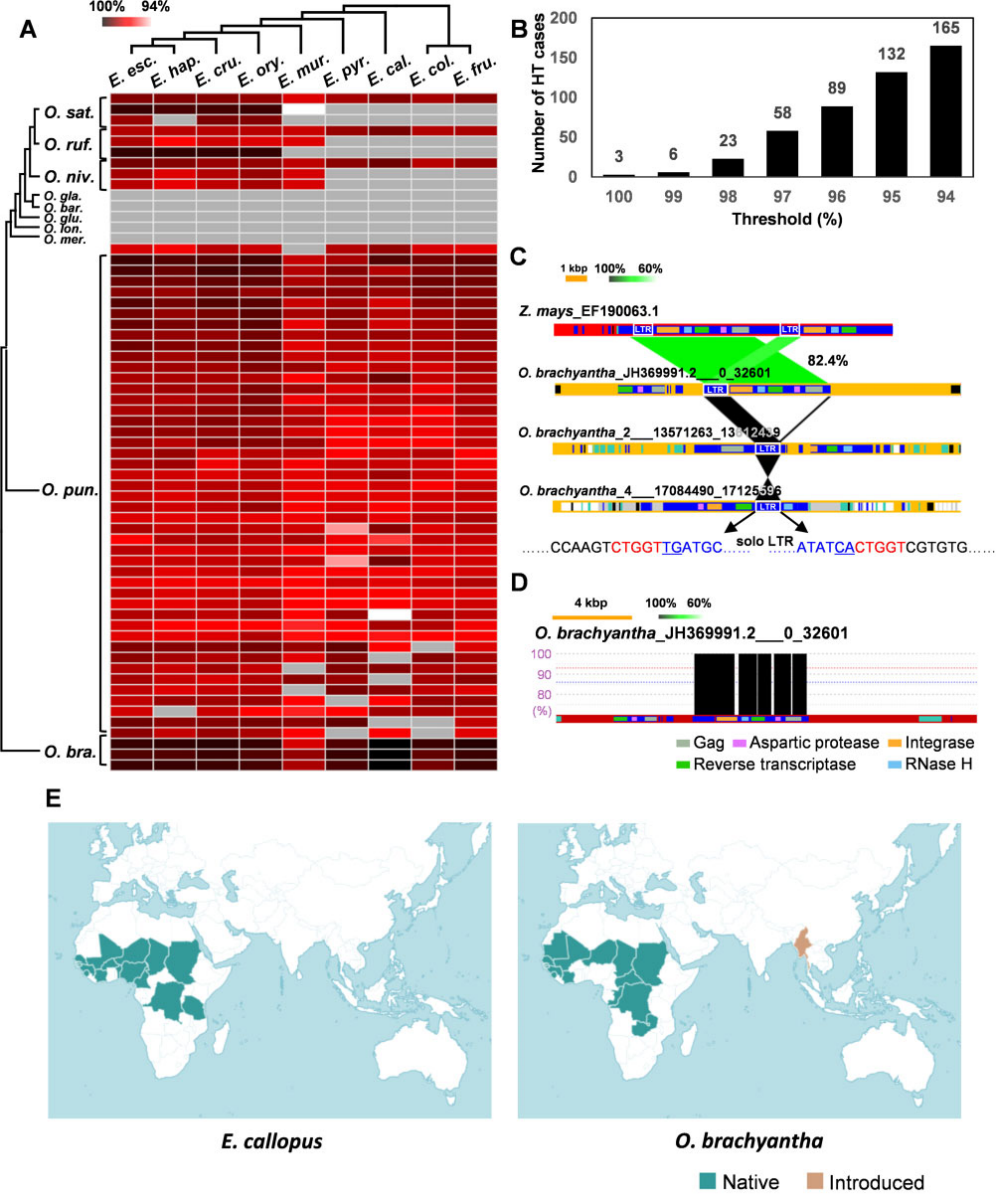
Summary of HTs detected between nine *Echinochloa* and ten *Oryza* species. (*A*) The heat map shows, for each HT shown as a separate row, the average identity of mapped reads between each pair of *Echinochloa* and *Oryza* species, sorted according to their phylogenetic tree shown on the left and top. Gray cells indicate no mapping. Abbreviations of *Echinochloa* species: *E. esc*., *E. esculenta*; *E. ory*., *E. oryzoides*; *E. cru*., *E. crus-galli*; *E. hap*., *E. haploclada*; *E. mur*., *E. muricata*; *E. pyr*., *E. pyramidalis*; *E. cal*., *E. callopus*; *E. col*., *E. colona*; and *E. fru*., *E. frumentacea*. (*B*) The number of HT cases identified is indicated for different sequence identity thresholds. (*C*) The genomic regions containing TEs that were horizontally transferred between *E. colona* and *O. brachyantha* are compared with the region of maize (*Zea mays*) containing a homolog of the transferred TE. Similar regions are connected, with colors indicating the degree of identity. LTR sequences are presented in the white box and the terminal sequences of the solo LTR are presented at the bottom of the figure. (*D*) For one LTR-retrotransposon, the reads from *E. callopus* are mapped onto the *O. brachyantha* genome, as shown with vertical black bars. The annotated LTR-retrotransposon and its domains are colored as in figure 2*D*. (*E*) Countries where the species *E. callopus* and *O. brachyantha* have been reported are colored.

The pooled reads of all *Echinochloa* sample sequences were mapped simultaneously to each of the *Oryza* genomes. Using a 97% identity threshold, a total of 58 densely mapped regions were detected (figs. 3 and 4*A* and supplementary fig. S8, Supplementary Material online). As expected, the number of predicted HT events varies with the threshold, with 3 cases at 100% and up to 165 at a 94% threshold (fig. 4*B* and supplementary fig. S8, Supplementary Material online). At high homology levels, all of the apparent HT events were of TEs (see below). While a lower threshold might result in more false positives, the broad distribution of these degrees of high similarity suggests that HTs have occurred frequently during the diversification of the *Echinochloa* and *Oryza* genera.

The pairwise identity between the different *Oryza* and *Echinochloa* species was reported for each of the 58 HTs detected with a 97% threshold (fig. 4*A*). Most fragments show differences between *Oryza* and *Echinochloa* sequences (<100% identity, fig. 4*A*), indicating that the exact donor was not sampled or mutations accumulated since the transfers. HTs detected in *O. sativa*, *O. rufipogon*, and *O. punctata* were more similar to sequences from the closely related *Echinochloa esculenta*, *Echinochloa crus-galli*, *Echinochloa oryzoides*, and *E. haploclada* (fig. 4*A*), indicating that the HT occurred from this lineage.

Interestingly, three regions that contained horizontally transferred sequences had a 100% identity between *Echinochloa callopus* and *Oryza brachyantha* (fig. 4*A*, *C*, and *D*). A comparison of the HT sequence with a maize ortholog indicated that the sequences from *O. brachyantha* represent three truncated versions of the same LTR-retrotransposon, likely derived from a single HT. One of the sequences corresponds to a full unit of an LTR-retrotransposon missing one LTR, another one to a fragment of the LTR sequence, and the third to a solo LTR (fig. 4*C*). The putative donor and recipient of this HT have overlapping distributions in Central and West Africa (fig. 4*E*, Kew science database; http://powo.science.kew.org/, last accessed May 12, 2021), offering opportunities for a very recent transfer. These patterns suggest that *E. callopus* and *O. brachyantha* are the direct donor and recipient, respectively, of the HT of this LTR-retrotransposon.

**Fig. 5.**
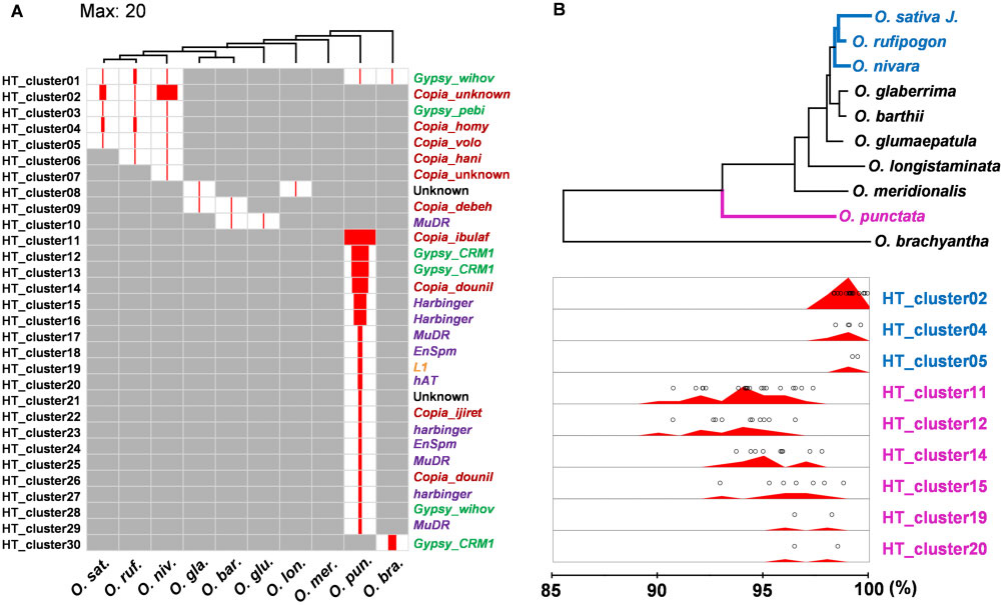
Types and relative activity histories of horizontally transferred TEs between *Echinochloa* and *Oryza*. (*A*) For each *Oryza* species, sorted according to their phylogenetic tree shown at the top, the number of TEs in a genome derived from its horizontal transfer is indicated by the size of the red rectangle (up to 20 TEs). The names of the HT clusters are shown on the left and those of classified TE families on the right. (*B*) Below the phylogenetic tree of *Oryza*, the relative activity history is shown for TEs in nine HT clusters. In the activity history, the dots indicate branching points of horizontally transferred TEs calculated by the UPGMA algorithm. The density of the points is depicted by the red graph in the panel.

The type of DNA and their relative activity history were investigated for the 165 HTs identified with the 94% threshold (supplementary fig. S8, Supplementary Material online). These include 39 *Gypsy* and 70 *Copia* LTR-retrotransposons, 49 DNA transposons, three LINE retrotransposons, and four unknown TEs (supplementary tables S4 and S5, Supplementary Material online). Because many of the 165 detected elements might result from amplification after HT events, all the detected TEs were clustered based on sequence similarity. A total of 30 clusters that contain at least one horizontally transferred sequence (HT clusters) were generated from 147 horizontally transferred TEs, and the 18 other horizontally transferred TEs were identified as singletons (fig. 5*A*, supplementary tables S4 and S5, Supplementary Material online). Because each cluster must have resulted from at least one HT, we calculate that there were a minimum of 48 HT events; one for each of the 30 HT clusters and one for each of the 18 singletons.

Among *Oryza* species, the largest number of HT events were detected in *O. punctata*. A total of 27 out of 48 HT events (19 HT clusters numbered 11–29 in figure 5*A* and eight out of the 18 singletons in supplementary table S5, Supplementary Material online) were unique to the *O. punctata* genome. Unlike other *Oryza* species, most HTs detected in *O. punctata* involved DNA transposons (16 out of 27 HTs; eight classified as *Harbinger* and four as *MuDR*) and non-LTR retrotransposons (one out of 27 HTs). Multiple clusters of horizontally transferred sequences detected in *O. puncata* were classified as the same TE family, but the clusters did not closely align to each other, confirming that they are only distantly related.

The relative activity history of the horizontally transferred TEs could be estimated from nine HT clusters based on the UPGMA algorithm (fig. 5*B*). HT clusters 02, 04, and 05 were found in *O. sativa*, *O. rufipogon*, and *O. nivara* (fig. 5*B*, marked with blue) and they were amplified in each species after the HT event, leading to numerous copies in some species (fig. 5*A*). HT clusters 11, 12, 14, 15, 19, and 20 (fig. 5*B*, marked with pink), all detected solely in *O. punctata*, amplified at different times and remained active for long periods (fig. 5*B*).

## Discussion

### The Frequency of HTs of TEs into Plant Genomes

The dynamic activity of TEs provides plasticity to plant genomes. A TE family can increase its population size through its amplification activity, and it also can become extinct by deletion or degeneration in a plant genome (Kaplan et al. 1985). Because it is an irreversible phenomenon, the repeated extinction of TE families would gradually reduce TE diversity. In our study, dense sampling of a group of donors and a group of recipients allowed direct assessments of the dynamics of HT of TEs. We identified a minimum of 27 independent HT events (nineteen from the HT clusters and eight from the singletons) between the *Echinochloa* genus and the *O. punctata* lineage (fig. 5*A* and supplementary table S5, Supplementary Material online), by far the most active exchange history detected. Interestingly, these HTs involved diverse types of TEs, such as *Gypsy* and *Copia* LTR-retrotransposons, the DNA transposons *Harbinger*, *MuDR*, *EnSpm*, and *hAT*, and the *L1* non-LTR retrotransposon family. Moreover, many (perhaps all) of these TEs were amplified in the host genome after the HT events (fig. 5*B* and supplementary fig. S6, Supplementary Material online). Therefore, we provide direct evidence that HT of TEs can play an important role in maintaining or increasing the diversity of the TE families against the extinction of TE families.

Because of lineage sorting of initially hemizygous TE transpositions, one always expects to find only a tiny minority of the TE insertions generated over evolutionary time in any single haplotype. Hence, the discoveries in this analysis only provide a minimum estimate of the true frequencies of events. Moreover, HT of TEs or other types of sequences might be expected to be most frequent between closely related plants, because of basic genetic compatibility, but these were excluded from our study because of an inability to distinguish between horizontally transferred sequences that are highly homologous and sequences that are highly homologous merely by vertical descent from common recent ancestors. Finally, horizontally transferred sequences that did not amplify, were from very ancient HT events, or were selected against would also be underrepresented in our analysis. Hence, it appears that HT is very common between some plant lineages, even those that have diverged from a common ancestor for >50 My, as is the case for the panicoid grasses and the genus *Oryza*.

### Possible Mechanisms of the HT of TEs

Despite repeated reports of HT among plants, the underlying mechanism remains unknown. In the case of TEs, several scenarios are possible. For instance, an HT could involve a large fragment of DNA containing TEs that are subsequently amplified (Dunning et al. 2019). Or the TEs could move on their own, perhaps as an LTR-retrotransposon that occasionally acts as a retrovirus. How plant tissues interact to allow this transfer to occur is another issue, with tissue wounding and insect-mediation or interspecies root cell–cell interactions all as possibilities.

No evidence of HT of a large DNA fragment was found in this study, despite 100% similarities suggesting recent transfers (fig. 4*A*). The numerous HTs detected in *O. punctata* involved LTR-retrotransposons, DNA transposons, and non-LTR-retrotransposons, indicating that multiple types of TEs can be exchanged. This great variety in TE types that underwent HT demonstrates that the movements do not rely on any specialized function specific to one type of TE.

Massive HTs of TEs were detected after analyzing 195 insect genomes (Peccoud et al. 2017), and we suggest that insects might also move TEs among plants. Plant TEs can be activated with wound stress caused by physical damages from insects (Wessler 1996; Takeda et al. 1998), and they could therefore be transferred temporarily to the insect, which could place them into the wounds of the next plant it attacks. For example, aphids are known to act as a vector of viruses among plants (Ng and Perry 2004). It is known that the grafting of two sexually incompatible plants can transfer the chloroplast genome and cause HT (Stegemann et al. 2012). With a similar mechanism, DNA fragments transferred by insects might integrate into the genome of a gametophytic or meristematic cell, resulting in HT. Because the *Echinochloa* genus includes species that are well-known as rice paddy weeds (Bhagirath and David 2009), insect-mediated, or root–root interaction-based HTs could occur in this environment, perhaps explaining their preponderance in our analyses.

Our nontargeted investigation identified no HT of standard nuclear protein-encoding genes. With the exception of LINEs and SINEs, all TEs have a DNA intermediate during their replication. Because many of the HTs that we identified consisted of an intact unit of a TE that was able to subsequently proliferate, the transfer likely involved the DNA form of the intermediate. By contrast, genes are not routinely mobile, but rather are transcribed from chromosomes in the form of RNA, which is less stable and lacks an intrinsic integration property. DNA transposons were more frequently transferred among insects than retrotransposons (Peccoud et al. 2017), and 16 of the 27 HT events identified in *O. punctata* involved DNA transposons. The relatively high number of DNA transposons transferred into *O. punctata* suggests that DNA-type TEs have been particularly active in this species and/or in the donor lineages that have contributed to its genome. We conclude that the extrachromosomal stability of the TEs coupled with their propensity for self-replication likely accounts for the numerous successful HTs identified here.

## Conclusions

One of the biggest bottlenecks in studying HTs among angiosperms is the lack of abundant sequence resources (Wallau et al. 2018). In this study, we show that low-depth sample sequence analyses can solve the genome sequence shortage by generating data that can be used to detect HTs for large numbers of species. We first scanned the genomes of 115 angiosperms with sequences belonging to 19 panicoid grasses. Despite the large number of species, putative HTs were initially detected only to *O. sativa*. These results suggest that HT happens frequently only between some groups of plants. Previous studies have reported HT between other distantly related groups of grasses (Mahelka et al. 2017; Hibdige et al. 2020), demonstrating that the phenomenon is widespread at least among some grasses. The small number of HTs identified in our first scan may thus reflect computational limitations. First, the number of HTs detected depends on the similarity threshold (fig. 4*B*). Second, relying on high similarities means that only recent HT can be detected. Large-scale surveys with diverse species are consequently required, and we have shown that increasing either the number of potential recipients or donors increases the number of HTs detected. Because sample sequences, such as those used in this study, are becoming available for large numbers of species, we predict that our method will allow large-scale systematic detections of HTs in the future across hundreds or thousands of sample-sequenced lineages.

## Materials and Methods

### Low-Depth Sample Sequencing

A total of 25 panicoid species were selected for analyses, most being grown in a comparative experiment by Atkinson et al. (2016) (supplementary table S1, Supplementary Material online). Genomic DNAs were isolated from leaf tissue using Qiagen spin columns (DNeasy Plant Mini Kit; Qiagen). Illumina NextSeq was used for sequencing. The sequences generated were targeted to be ∼151 bp single-end reads. The sequences used were at least 100 bp in length after quality-trimming.

### LTR-Retrotransposon-Targeted Search for HT

To look into possible HT of LTR-retrotransposons from the 19 studied panicoid species, we compared the panicoid RTs that we discovered to 115 publicly available plant genome sequences. These 115 plant genome sequences are listed and referenced in supplementary table S2, Supplementary Material online, and were chosen because they covered a great breadth of angiosperm diversity and/or because they were considered reference or otherwise highly assembled genomes. Reference sequences from other panicoid genomes (e.g., maize, sorghum, foxtail millet, pearl millet, proso millet) were not used in the analysis because their genes and TEs are closely related to those in the studied panicoids by shared vertical descent. Hence, the closest relatives to the panicoids of these 115 species were grasses in the Pooid and Oryzoid subfamilies of the Poaceae, which last shared a common ancestor with the panicoids >50 Ma (Christin et al. 2014).

To estimate the degree of divergence between some of the reference genomes and the 19 panicoids, we used the CDS of all of their nuclear genes. The CDS were compared with the panicoid sample sequences by BLAST (single HSP, score: >60, and *e*-value: <*e*-8). In each case, the best nonoverlapping hits among the mapped panicoid sample sequences were considered. The hits corresponding to possible paralogs would be impossible to discern from orthologs for these short and unassembled sample reads, so they were not removed. Histograms were drawn showing the identity of the compared sequences, and the peak point was considered as an indication of the approximate speciation point.

All of the RT sequence reads were isolated from the 19 panicoid sample sequences with HMMER (default options) (Mistry et al. 2013) using customized HMM profiles for the short-read sequences (supplementary additional files 1 and 2, Supplementary Material online). The isolated RT sequences were clustered based on sequence similarity with the RepeatExplorer pipeline (default options) (Novák et al. 2013). As a result, a total of 368 RT clusters was generated. Each of the RTs was compared with the 115 plant genome sequences by BLAST (single HSP, score: >60, and *e*-value: <*e*-8). The top three homologies with >85% identity and at least 140 bp matched length were considered. Using their position information, the sequence contig of the target genome containing the match was isolated with 10 kb flanking regions on each side. The isolated 20 kb-length sequence contigs were compared again to all of the 19 panicoid sample sequences by BLAST (single HSP, score: >60, and *e*-value: <*e*-8) to find all high homologies. From the BLAST results, the best hits in each position across the whole sequence contig were isolated with the threshold of a minimum of 100 bp matched length and a maximum of 75 bp overlap with other homologies. If there were a minimum of 10 hits within a LTR-retrotransposon, and their average identity was >97.0%, the sequence was considered as a horizontally transferred LTR-retrotransposon. The HT of panicoid LTR-retrotransposons into the ten *Oryza* species was investigated by the same method. The horizontally transferred LTR-retrotransposons were classified with the maize repeat database (supplementary additional file 3, Supplementary Material online).

Homology/annotation of the five domains of LTR-retrotransposons to the target genome sequence contigs was carried out with HMMER 3.0 (default options). The HMM profiles for the five standard LTR-retrotransposon domains were downloaded from GyDB. The position information of the annotated domains was used for identifying the candidate LTR-retrotransposons.

Homologous copies of horizontally transferred LTR-retrotransposons in the ten *Oryza* species were identified by comparing the RT sequences of the horizontally transferred LTR-retrotransposons of panicoids to all the *Oryza* genome sequences by BLAST (single HSP, score: >60, and *e*-value: <*e*-8). From the BLAST results, a matched length longer than half of the query RT sequence and with identity >85% was considered a homologous copy of the horizontally transferred LTR-retrotransposon. The identity of these homologous copies was used for drawing the identity histograms. All of the analyses and visualizations of the results were carried out with in-house python scripts.

### Nontargeted Search for HT

The sample sequences of the nine *Echinochloa* species were mapped to the ten *Oryza* genome sequences with bowtie 1.2.0 (default options) (Langmead 2010). To use a 94% threshold for the initial search, we generated 50 bp short-read sequences and mapped them by allowing a maximum of three mismatches. With this threshold, if the length of mapped regions in the target *Oryza* genomes was >500 bp and the interval between mapped sequences was <150 bp, we isolated the mapped region with 20 kb flanking regions on both sides. The 150 bp-long sample sequences of all the *Echinochloa* species were mapped again to the isolated target sequence contigs with BLAST (single HSP, score: >60, and *e*-value: <*e*-8). The target sequences mapped with the *Echinochloa* sample sequences by >97% identity and >500 bp mapped region were considered as candidate contigs containing horizontally transferred fragments. The candidate contigs were filtered by removing the contigs if the mapped region corresponded to organelle genome sequences, ribosomal sequences, simple repeat sequences, or highly conserved genes. After filtering, the horizontally transferred fragments were clustered with RepeatExplorer (default options). The clustered and nonclustered elements were classified using the maize repeat database. In the case of non-LTR retroelements, their identities were further confirmed by finding RT domains with the non-LTR retroelement domain search tool, RT1class1 (http://www.girinst.org/RTphylogeny/RTclass1, last accessed May 12, 2021) (supplementary additional file 4, Supplementary Material online).

### Phylogenetic Analysis of Panicoids

The long single copy part of the maize chloroplast sequence (∼82 kb) (Maier et al. 1995) was used as the reference. Reads from each of the 25 panicoid sample sequences mapping to this reference were identified and assembled into species-specific contigs by Velvet 1.2.10 with default options (Zerbino and Birney 2008). The assembled chloroplast sequences were corrected again by mapping reads, and a > 90% consensus was extracted in Geneious (Kearse et al. 2012). The chloroplast sequences were aligned using MAFFT v. 7.392 (Katoh and Standley 2013) with six other chloroplast sequences (*Phragmites australis, Aristida rufescens, Chloris virgata, O. sativa, Brachypodium distachyon*, and *Bambusa multiplex*) obtained from GenBank, with the three last species used to root the tree. Trimal v. 1.4.rev6 (Capella-Gutiérrez et al. 2009) was used to remove all sites with ambiguous or missing data. The 56,388 bp alignment was used to infer a maximum-likelihood phylogenetic tree with RAxML v. 8.2.12 (Stamatakis 2014). Using the GTRCAT model, the best tree out of ten runs was identified, and support values were then estimated with 100 bootstrap pseudoreplicates. The inferred relationships mirrored those based on coalescence analyses of nuclear genes (Dunning et al. 2019). A similar approach was used to infer a tree for nine *Echinochloa* species, except that the plastid genome sequence from *Setaria italica* was used as the outgroup.

### Phylogenetic Tree for HT Analysis

RTs in the *Oryza* genomes were identified through BLAST searches (single HSP, score: >60, and *e*-value: <*e*-8, aligned length > 100 bp), using the panicoid RT sequences as the query from the clusters containing the horizontally transferred sequences. All RTs with at least one hit from the panicoids were retrieved from the *Oryza* genomes, and aligned to infer a phylogenetic tree. The alignment was conducted with MUSCLE as implemented in MEGA7.0 software (Kumar et al. 2016), and the phylogenetic tree was constructed by the Neighbor-Joining method (*p*-distance and pairwise deletion).

### Analysis of Intrafamily TE Divergence to Determine Transposition History

The UPGMA algorithm (Legendre and Legendre 1998) was used to estimate the relative activity history of TEs based on pairwise distances. For this, the pairwise sequence identity of the TE sequences was calculated in each cluster by all-to-all BLAST analyses (single HSP, score: >60, and *e*-value: <*e*-8). Based on the UPGMA algorithm, the phylogenetic relatedness and its node values were calculated from the pairwise distances of the RT sequences. The software used to calculate the relative activity history of TEs is found in supplementary additional file 5, Supplementary Material online. The distances at all the nodes were used for drawing identity histograms to estimate the relative activity history of TEs. The histograms were drawn by counting the number of nodes in each of the one percent intervals. Calculation of the distances at each node and visualization of the results were performed with two in-house python scripts, one to calculate the distances at each branching point and the other to visualize the results.

### Phylogenetic Tree of Ten *Oryza* Species

To construct a phylogenetic tree of the ten *Oryza* species, orthologous CDSs of all nuclear genes were identified as the reciprocal best hits from BLAST searches (single HSP, score: >150, and *e*-value: <*e*-8). Each of the orthologous CDSs was translated into the amino acid sequence, and the amino acid sequences were aligned with prank (Löytynoja and Goldman 2010). The unaligned regions were trimmed, and the remaining sequences were converted back into CDS. The third positions of codons of all sequences were concatenated, and a phylogenetic tree was inferred with RAxML, as described above. The inferred relationships mirrored those inferred in previous studies (Zhu and Ge 2005; Gao et al. 2019).

## Supplementary Material

Supplementary data are available at *Molecular Biology and Evolution* online.

## Supplementary Material

msab133_Supplementary_Data
