# Supplementary material for: Sample Sequence Analysis Uncovers Recurrent Horizontal Transfers of Transposable Elements among Grasses
Source: Mol Biol Evol. 2021 May 8;38(9):3664–75. doi: 10.1093/molbev/msab133 (PMC8382918; doi:10.1093/molbev/msab133)
Supplement: msab133_Supplementary_Data [file msab133_supplementary_data.zip › Additional_File_4_L1_RTclass1_result.html]

1614864178
The 
Oryzapunctata317790280-17791703-1 
reverse transcriptase belongs to the 

clade
  
  
                                                                                                                                                      Families                                Species          Clades
  
  
                                                                                                                                              +------ R2-1\_SM                                 planaria          R2       
                                                                                                                                       +390.0-|                                               
                                                                                                                                +157.0-|      +------ R2\_DM                                   fly               R2       
                                                                                                                                |      |                                               
                                                                                                                         +-94.0-|      +------------- PERERE-9                                planaria          R2       
                                                                                                                         |      |                                               
                                                                                                                  +303.0-|      +-------------------- R2\_AM                                   collembola        R2       
                                                                                                                  |      |                                               
                                                                                                                  |      |                    +------ R2-1\_PM                                 lamprey           R2       
                                                                                                           +662.0-|      +--------------373.0-|                                               
                                                                                                           |      |                           +------ R2Ci-B                                  tunicate          R2       
                                                                                                           |      |                                               
                                                                                                           |      +---------------------------------- R2\_PS                                   crustacea         R2       
                                                                                                    +949.0-|                                               
                                                                                                    |      |                                  +------ R2Dr                                    fish              R2       
                                                                                                    |      |                           +-1000-|                                               
                                                                                                    |      |                    +992.0-|      +------ R2-2\_PM                                 lamprey           R2       
                                                                                                    |      |                    |      |                                               
                                                                                                    |      +--------------632.0-|      +------------- R2\_LP                                   chelicerata       R2       
                                                                                                    |                           |                                               
                                                                                                    |                           +-------------------- R2-1\_TSP                                nematoda          R2       
                                                                                                    |                                               
                                                                                             +638.0-|                                         +------ R2-1a\_Cis                               tunicate          NeSL       
                                                                                             |      |                                  +-1000-|                                               
                                                                                             |      |                           +539.0-|      +------ YURECi                                  tunicate          NeSL       
                                                                                             |      |                           |      |                                               
                                                                                             |      |             +-------779.0-|      +------------- NeSL-1                                  nematoda          NeSL       
                                                                                             |      |             |             |                                               
                                                                                             |      |             |             |             +------ R2I-1\_PI                                protozoa          NeSL       
                                                                                             |      |             |             +--------1000-|                                               
                                                                                             |      |             |                           +------ R2I-2\_PI                                protozoa          NeSL       
                                                                                             |      |             |                                               
                                                                                             |      +-------791.0-|                           +------ LIN9\_SM                                 planaria          NeSL       
                                                                                             |                    |                    +771.0-|                                               
                                                                                             |                    |             +421.0-|      +------ R5-1\_SM                                 planaria          NeSL       
                                                                                             |                    |             |      |                                               
                                                                                      +577.0-|                    |      +888.0-|      +------------- R5-2\_SM                                 planaria          NeSL       
                                                                                      |      |                    |      |      |                                               
                                                                                      |      |                    +682.0-|      +-------------------- R5                                      planaria          NeSL       
                                                                                      |      |                           |                                               
                                                                                      |      |                           +--------------------------- NeSL-1\_TV                               protozoa          NeSL       
                                                                                      |      |                                               
                                                                                      |      |                                                +------ HERO-1\_BF                               amphioxus         Hero       
                                                                                      |      |                                         +985.0-|                                               
                                                                                      |      |                                  +931.0-|      +------ HEROTn                                  fish              Hero       
                                                                                      |      |                                  |      |                                               
                                                                               +251.0-|      |                           +998.0-|      +------------- HERO-1\_SP                               urchin            Hero       
                                                                               |      |      |                           |      |                                               
                                                                               |      |      |                    +610.0-|      +-------------------- HERO-1\_PP                               slime\_mold        Hero       
                                                                               |      |      |                    |      |                                               
                                                                               |      |      +--------------849.0-|      +--------------------------- HERO-1\_HR                               leech             Hero       
                                                                               |      |                           |                                               
                                                                               |      |                           +---------------------------------- HERO-3\_BF                               amphiopxus        Hero       
                                                                               |      |                                               
                                                                               |      |                                                +------------- RandI-4                                 green\_algae       RandI       
                                                                               |      |                                         +698.0-|                                               
                                                                               |      |                                         |      |      +------ RandI-1                                 green\_algae       RandI       
                                                                               |      +------------------------------------1000-|      +967.0-|                                               
                                                                               |                                                |             +------ RandI-1\_VC                              green\_algae       RandI       
  +----------------------------------------------------------------------181.0-|                                                |                                               
  |                                                                            |                                                +-------------------- RandI-6                                 green\_algae       RandI       
  |                                                                            |                                               
  |                                                                            |                                                       +------------- DongAG                                  mosquito          R4       
  |                                                                            |                                                +979.0-|                                               
  |                                                                            |                                                |      |      +------ R4-1\_AC                                 lizard            R4       
  |                                                                            |                                         +984.0-|      +-1000-|                                               
  |                                                                            |                                         |      |             +------ Rex6                                    fish              R4       
  |                                                                            |                                         |      |                                               
  |                                                                            |                                  +-1000-|      +-------------------- R4\_AL                                   nematoda          R4       
  |                                                                            |                                  |      |                                               
  |                                                                            |                                  |      |                    +------ R4-1\_ED                                 protozoa          R4       
  |                                                                            |                                  |      |             +984.0-|                                               
  |                                                                            |                                  |      +--------1000-|      +------ EhRLE2                                  protozoa          R4       
  |                                                                            +----------------------------242.0-|                    |                                               
  |                                                                                                               |                    +------------- EhRLE3                                  protozoa          R4       
  |                                                                                                               |                                               
  |                                                                                                               |                           +------ Proto1-1\_NG                             protozoa          Proto1       
  |                                                                                                               |                    +-1000-|                                               
  |                                                                                                               +---------------1000-|      +------ Proto1-4\_NG                             protozoa          Proto1       
  |                                                                                                                                    |                                               
  |                                                                                                                                    +------------- Proto1-6\_NG                             protozoa          Proto1       
  |                                               
  |                                                                                                                                           +------ CR1-65\_HM                               cnidaria          Daphne       
  |                                                                                                                                    +841.0-|                                               
  |                                                                                                                                    |      +------ CR1-7\_HM                                cnidaria          Daphne       
  |                                                                                                                             +321.0-|                                               
  |                                                                                                                             |      |      +------ CR1-14\_NV                               cnidaria          Daphne       
  |                                                                                                                      +201.0-|      +885.0-|                                               
  |                                                                                                                      |      |             +------ CR1-23\_BF                               amphioxus         Daphne       
  |                                                                                                                      |      |                                               
  |                                                                                                               +421.0-|      +-------------------- Crack-1\_CS1                             annelida          Daphne       
  |                                                                                                               |      |                                               
  |                                                                                                               |      |                    +------ Crack-1\_NV                              cnidaria          Daphne       
  |                                                                                                        +330.0-|      +--------------351.0-|                                               
  |                                                                                                        |      |                           +------ CR1-21\_SP                               urchin            Daphne       
  |                                                                                                 +566.0-|      |                                               
  |                                                                                                 |      |      +---------------------------------- L2-4\_Cis                                tunicate          Daphne       
  |                                                                                                 |      |                                               
  |                                                                                                 |      +----------------------------------------- CR1-12\_SP                               urchin            Daphne       
  |                                                                                                 |                                               
  |                                                                                          +331.0-|                                  +------------- Crack-3\_CP                              mosquito          Daphne       
  |                                                                                          |      |                           +-1000-|                                               
  |                                                                                          |      |                           |      |      +------ Crack-2\_CP                              mosquito          Daphne       
  |                                                                                          |      |                    +766.0-|      +-1000-|                                               
  |                                                                                          |      |                    |      |             +------ Crack-1\_CP                              mosquito          Daphne       
  |                                                                                          |      +--------------289.0-|      |                                               
  |                                                                                          |                           |      +-------------------- Crack-4\_CP                              mosquito          Daphne       
  |                                                                                   +441.0-|                           |                                               
  |                                                                                   |      |                           +--------------------------- L2-2\_Cis                                tunicate          Daphne       
  |                                                                                   |      |                                               
  |                                                                                   |      |                                                +------ Daphne-1\_TCa                            beetle            Daphne       
  |                                                                                   |      |                                         +433.0-|                                               
  |                                                                            +676.0-|      |                                  +388.0-|      +------ Daphne-1\_BM                             silkworm          Daphne       
  |                                                                            |      |      |                                  |      |                                               
  |                                                                            |      |      +----------------------------503.0-|      +------------- Sake\_BM                                 silkworm          Daphne       
  |                                                                            |      |                                         |                                               
  |                                                                            |      |                                         +-------------------- Crack-1\_IC                              tick              Daphne       
  |                                                                            |      |                                               
  |                                                                     +211.0-|      +-------------------------------------------------------------- Daphne\_DS                               crustacea         Daphne       
  |                                                                     |      |                                               
  |                                                                     |      |                                                       +------------- Crack-24\_BF                             amphioxus         Crack       
  |                                                                     |      |                                                +706.0-|                                               
  |                                                                     |      |                                                |      |      +------ Crack-7\_BF                              amphioxus         Crack       
  |                                                                     |      |                                                |      +973.0-|                                               
  |                                                                     |      +------------------------------------------246.0-|             +------ Crack-1\_SP                              urchin            Crack       
  |                                                                     |                                                       |                                               
  |                                                                     |                                                       |      +------------- L2B-1\_HM                                cnidaria          L2B       
  |                                                              +260.0-|                                                       +993.0-|                                               
  |                                                              |      |                                                              |      +------ L2B-1\_CP                                mosquito          L2B       
  |                                                              |      |                                                              +999.0-|                                               
  |                                                              |      |                                                                     +------ CR1-1\_AG                                mosquito          L2B       
  |                                                              |      |                                               
  |                                                              |      |                                                                     +------ Kiri-1\_AAe                              mosquito          Kiri       
  |                                                              |      |                                                              +912.0-|                                               
  |                                                              |      |                                                       +-1000-|      +------ Kiri-8\_CQ                               mosquito          Kiri       
  |                                                              |      |                                                       |      |                                               
  |                                                       +316.0-|      +-------------------------------------------------507.0-|      +------------- Kiri-1\_CQ                               mosquito          Kiri       
  |                                                       |      |                                                              |                                               
  |                                                       |      |                                                              +-------------------- Crack-1\_PM                              lamprey           Crack       
  |                                                       |      |                                               
  |                                                       |      |                                                                            +------ L2A                                     mammals           L2       
  |                                                       |      |                                                                     +974.0-|                                               
  |                                                       |      |                                                +---------------1000-|      +------ CR1-L2-1\_XT                             frog              L2       
  |                                                       |      |                                                |                    |                                               
  |                                                       |      |                                                |                    +------------- CR1-2\_DR                                fish              L2       
  |                                                       |      |                                                |                                               
  |                                                       |      +-------------------------------------------1000-|                    +------------- CR1-16\_NV                               cnidaria          L2       
  |                                                       |                                                       |      +-------914.0-|                                               
  |                                                       |                                                       |      |             |      +------ CR1-17\_NV                               cnidaria          L2       
  |                                                       |                                                       |      |             +555.0-|                                               
  |                                                +835.0-|                                                       |      |                    +------ CR1-1\_NV                                cnidaria          L2       
  |                                                |      |                                                       +633.0-|                                               
  |                                                |      |                                                              |                    +------ L2-3\_GA                                 fish              L2       
  |                                                |      |                                                              |             +691.0-|                                               
  |                                                |      |                                                              |      +505.0-|      +------ CR1-1\_DR                                fish              L2       
  |                                                |      |                                                              |      |      |                                               
  |                                                |      |                                                              +785.0-|      +------------- L2-5\_XT                                 frog              L2       
  |                                                |      |                                                                     |                                               
  |                                                |      |                                                                     |      +------------- CR1-3\_Lme                               latimeria         L2       
  |                                                |      |                                                                     +361.0-|                                               
  |                                                |      |                                                                            |      +------ L2-1\_ACar                               lizard            L2       
  |                                                |      |                                                                            +784.0-|                                               
  |                                                |      |                                                                                   +------ L2-2\_ACar                               lizard            L2       
  |                                                |      |                                               
  |                                                |      |                                                                                   +------ CR1-34\_HM                               cnidaria          L2A       
  |                                                |      +-----------------------------------------------------------------------------934.0-|                                               
  |                                                |                                                                                          +------ L2-24\_NV                                cnidaria          L2A       
  |                                                |                                               
  |                                                |                                                                                          +------ CR1-1\_BM                                silkworm          CR1       
  |                                                |                                                                                   +732.0-|                                               
  |                                                |                                                                                   |      +------ DMCR1A                                  fly               CR1       
  |                                                |                                                                            +467.0-|                                               
  |                                                |                                                                            |      |      +------ CR1-2\_IS                                tick              CR1       
  |                                                |                                                                            |      +999.0-|                                               
  |                                                |                                  +-----------------------------------971.0-|             +------ CR1-1\_IS                                tick              CR1       
  |                                                |                                  |                                         |                                               
  |                                                |                                  |                                         |             +------ T1                                      mosquito          CR1       
  |                                                |                                  |                                         +--------1000-|                                               
  |                                                |                                  |                                                       +------ CR1-2\_AG                                mosquito          CR1       
  |                                                |                                  |                                               
  |                                         +812.0-|                                  |                                                +------------- CR1-2\_HRo                               annelida          CR1       
  |                                         |      |                                  |                                  +--------1000-|                                               
  |                                         |      |                                  |                                  |             |      +------ PlatCR1                                 turtle            CR1       
  |                                         |      |                                  |                                  |             +771.0-|                                               
  |                                         |      |                                  |                                  |                    +------ CR1\_1a\_XT                               frog              CR1       
  |                                         |      |                                  |                           +254.0-|                                               
  |                                         |      |                                  |                           |      |                    +------ CR1-32\_BF                               amphioxus         CR1       
  |                                         |      |                                  |                           |      |             +751.0-|                                               
  |                                         |      |                                  |                           |      |      +451.0-|      +------ CR1-24\_SP                               urchin            CR1       
  |                                         |      |                           +681.0-|                           |      |      |      |                                               
  |                                         |      |                           |      |                           |      +927.0-|      +------------- CR1-21\_BF                               amphioxus         CR1       
  |                                         |      |                           |      |                    +665.0-|             |                                               
  |                                         |      |                           |      |                    |      |             +-------------------- CR1-25\_SP                               urchin            CR1       
  |                                         |      |                           |      |                    |      |                                               
  |                                         |      |                           |      |                    |      |                    +------------- CR1-7\_HRo                               annelida          CR1       
  |                                         |      |                           |      |                    |      |             +984.0-|                                               
  |                                         |      |                           |      |             +883.0-|      |             |      |      +------ CR1-23\_HM                               cnidaria          CR1       
  |                                         |      |                           |      |             |      |      +-------602.0-|      +555.0-|                                               
  |                                         |      |                           |      |             |      |                    |             +------ CR1-36\_HM                               cnidaria          CR1       
  |                                         |      |                           |      |             |      |                    |                                               
  |                                         |      |                           |      |             |      |                    +-------------------- CR1-19\_HM                               cnidaria          CR1       
  |                                         |      |                    +510.0-|      |      +514.0-|      |                                               
  |                                         |      |                    |      |      |      |      |      +----------------------------------------- Perere-5                                planaria          CR1       
  |                                         |      |                    |      |      |      |      |                                               
  |                                         |      |                    |      |      |      |      |                                         +------ CR1-3\_NV                                cnidaria          CR1       
  |                                         |      |                    |      |      +840.0-|      |                                  +453.0-|                                               
  |                                         |      |                    |      |             |      +----------------------------983.0-|      +------ CR1-2\_NV                                cnidaria          CR1       
  |                                  +900.0-|      |                    |      |             |                                         |                                               
  |                                  |      |      |                    |      |             |                                         +------------- CR1-5\_NV                                cnidaria          CR1       
  |                                  |      |      |                    |      |             |                                               
  |                                  |      |      |                    |      |             +------------------------------------------------------- CR1-4\_NV                                cnidaria          CR1       
  |                                  |      |      +--------------866.0-|      |                                               
  |                                  |      |                           |      |                                                              +------ LINE2B\_CE                               nematoda          CR1       
  |                                  |      |                           |      +--------------------------------------------------------998.0-|                                               
  |                                  |      |                           |                                                                     +------ LINE2C\_CB                               nematoda          CR1       
  |                                  |      |                           |                                               
  |                                  |      |                           |                                                                     +------ CR1-1\_HM                                cnidaria          CR1       
  |                                  |      |                           |                                                              +449.0-|                                               
  |                                  |      |                           |                                                              |      +------ ZENON\_BM                                silkworm          CR1       
  |                                  |      |                           |                                                       +535.0-|                                               
  |                                  |      |                           |                                                       |      |      +------ CR1-9\_BF                                amphioxus         CR1       
  |                                  |      |                           |                                                       |      +668.0-|                                               
  |                                  |      |                           +-------------------------------------------------747.0-|             +------ CR1-36\_BF                               amphioxus         CR1       
  |                                  |      |                                                                                   |                                               
  |                                  |      |                                                                                   |             +------ CR1-26\_BF                               amphioxus         CR1       
  |                                  |      |                                                                                   |      +727.0-|                                               
  |                                  |      |                                                                                   +684.0-|      +------ CR1-6\_BF                                amphioxus         CR1       
  |                                  |      |                                                                                          |                                               
  |                                  |      |                                                                                          +------------- CR1-1\_LG                                mollusc           CR1       
  |                                  |      |                                               
  |                                  |      |                                                                                          +------------- REX1-5\_XT                               frog              Rex1       
  |                                  |      |                                                                                   +-1000-|                                               
  |                                  |      |                                                                                   |      |      +------ REX1-4\_XT                               frog              Rex1       
  |                                  |      |                                                                                   |      +-1000-|                                               
  |                                  |      +-----------------------------------------------------------------------------999.0-|             +------ REX1-1\_DR                               fish              Rex1       
  |                                  |                                                                                          |                                               
  |                                  |                                                                                          |      +------------- CR1-10\_NV                               cnidaria          Rex1       
  |                                  |                                                                                          +-1000-|                                               
  |                                  |                                                                                                 |      +------ CR1-11\_NV                               cnidaria          Rex1       
  |                                  |                                                                                                 +-1000-|                                               
  |                                  |                                                                                                        +------ CR1-9\_NV                                cnidaria          Rex1       
  |                                  |                                               
  |                                  |                                                                                                        +------ Ingi-1\_BF                               amphioxus         Ingi       
  |                                  |                                                                                                 +526.0-|                                               
  |                                  |                                                                                          +971.0-|      +------ Ingi-2\_BF                               amphioxus         Ingi       
  |                                  |                                                                                          |      |                                               
  |                                  |                                                                                          |      +------------- Ingi-1\_Pp                               insects           Ingi       
  |                                  |                                                                                   +-1000-|                                               
  |                                  |                                                                                   |      |             +------ Ingi-1\_Tcas                             insects           Ingi       
  |                                  |                                                                                   |      |      +611.0-|                                               
  |                                  |                                                                                   |      +706.0-|      +------ Ingi-1\_AAl                              hedgehog          Ingi       
  |                                  |                                         +-----------------------------------547.0-|             |                                               
  |                                  |                                         |                                         |             +------------- Ingi-3\_SPur                             urchin            Ingi       
  |                                  |                                         |                                         |                                               
  |                                  |                                         |                                         |                    +------ Ingi-1\_Rpro                             insects           Ingi       
  |                                  |                                         |                                         |             +612.0-|                                               
  |                                  |                                         |                                         +-------744.0-|      +------ I-2\_AC                                  mollusc           Ingi       
  |                           +285.0-|                                         |                                                       |                                               
  |                           |      |                                         |                                                       +------------- I-1\_AC                                  mollusc           Ingi       
  |                           |      |                                         |                                               
  |                           |      |                                         |                                                              +------ I-5\_DR                                  fish              Nimb       
  |                           |      |                                         |                                                       +-1000-|                                               
  |                           |      |                                         |                                                +535.0-|      +------ I-1\_DR                                  fish              Nimb       
  |                           |      |                                         |                                                |      |                                               
  |                           |      |                                         |                                         +895.0-|      +------------- Nimb-1\_LG                               mollusc           Nimb       
  |                           |      |                                         |                                         |      |                                               
  |                           |      |                                         |                                  +682.0-|      +-------------------- I-1\_SP                                  urchin            Nimb       
  |                           |      |                                         |                                  |      |                                               
  |                           |      |                                         |                                  |      +--------------------------- I-1\_CI                                  tunicate          Nimb       
  |                           |      |                                  +258.0-|                                  |                                               
  |                           |      |                                  |      |                                  |                           +------ I-4\_AC                                  mollusc           Nimb       
  |                           |      |                                  |      |                           +801.0-|                    +-1000-|                                               
  |                           |      |                                  |      |                           |      |                    |      +------ I-3\_AC                                  mollusc           Nimb       
  |                           |      |                                  |      |                           |      |             +-1000-|                                               
  |                           |      |                                  |      |                           |      |             |      |      +------ I-3\_DR                                  fish              Nimb       
  |                           |      |                                  |      |                           |      |             |      +996.0-|                                               
  |                           |      |                                  |      |                    +328.0-|      +-------983.0-|             +------ nimbus                                  mollusc           Nimb       
  |                           |      |                                  |      |                    |      |                    |                                               
  |                           |      |                                  |      |                    |      |                    |             +------ I-1\_AA                                  mosquito          Nimb       
  |                           |      |                                  |      |                    |      |                    +-------642.0-|                                               
  |                           |      |                                  |      |             +256.0-|      |                                  +------ I-1\_BM                                  silkworm          Nimb       
  |                           |      |                                  |      |             |      |      |                                               
  |                           |      |                                  |      |             |      |      +----------------------------------------- I-1\_DP                                  crustacean        I       
  |                           |      |                                  |      |             |      |                                               
  |                           |      |                                  |      |      +197.0-|      |                                         +------ I-2\_BM                                  silkworm          I       
  |                           |      |                                  |      |      |      |      +-----------------------------------754.0-|                                               
  |                           |      |                                  |      |      |      |                                                +------ Loner                                   mosquito          I       
  |                           |      |                           +140.0-|      |      |      |                                               
  |                           |      |                           |      |      +373.0-|      |                                                +------ IVK\_DM                                  fly               I       
  |                           |      |                           |      |             |      +------------------------------------------990.0-|                                               
  |                           |      |                           |      |             |                                                       +------ I\_DM                                    fly               I       
  |                           |      |                           |      |             |                                               
  |                           |      |                           |      |             +-------------------------------------------------------------- Mosqul\_Aa2                              mosquito          I       
  |                           |      |                           |      |                                               
  |                           |      |                           |      |                                                                     +------ Jockey                                  fly               Jockey       
  |                           |      |                           |      |                                                              +888.0-|                                               
  |                           |      |                           |      |                                                       +-1000-|      +------ G5\_DM                                   fly               Jockey       
  |                           |      |                           |      |                                                       |      |                                               
  |                    +699.0-|      |                           |      |                                                +645.0-|      +------------- FW                                      fly               Jockey       
  |                    |      |      |                    +239.0-|      |                                                |      |                                               
  |                    |      |      |                    |      |      |                                                |      |             +------ LDT1                                    moth              Jockey       
  |                    |      |      |                    |      |      |                                         +659.0-|      +--------1000-|                                               
  |                    |      |      |                    |      |      |                                         |      |                    +------ BMC1                                    silkworm          Jockey       
  |                    |      |      |                    |      |      |                                  +420.0-|      |                                               
  |                    |      |      |                    |      |      |                                  |      |      +--------------------------- LINE-1\_AA                               mosquito          Jockey       
  |                    |      |      |                    |      |      +----------------------------695.0-|      |                                               
  |                    |      |      |                    |      |                                         |      +---------------------------------- Syrinx\_DS                               crustacea         Jockey       
  |                    |      |      |                    |      |                                         |                                               
  |                    |      |      +--------------374.0-|      |                                         +----------------------------------------- Hebe                                    rotifer           Jockey       
  |                    |      |                           |      |                                               
  |                    |      |                           |      |                                                                            +------ Outcast-2\_BF                            amphioxus         Outcast       
  |                    |      |                           |      |                                                                     +-1000-|                                               
  |                    |      |                           |      +---------------------------------------------------------------822.0-|      +------ Outcast-1\_BF                            amphioxus         Outcast       
  |                    |      |                           |                                                                            |                                               
  |                    |      |                           |                                                                            +------------- Outcast                                 mosquito          Outcast       
  |                    |      |                           |                                               
  |                    |      |                           |                                                                            +------------- L1\_TC                                   protozoa          Ingi       
  |                    |      |                           +----------------------------------------------------------------------601.0-|                                               
  |                    |      |                                                                                                        |      +------ Ingi                                    protozoa          Ingi       
  |                    |      |                                                                                                        +-1000-|                                               
  |                    |      |                                                                                                               +------ Ingi2                                   protozoa          Ingi       
  |                    |      |                                               
  |                    |      |                                                                                                        +------------- R1\_DM                                   fly               R1       
  |                    |      |                                                                                                 +647.0-|                                               
  |                    |      |                                                                                                 |      |      +------ TRAS1                                   silkworm          R1       
  |                    |      |                                                                                                 |      +867.0-|                                               
  |                    |      |                                                                                   +-------991.0-|             +------ R1                                      silkworm          R1       
  |                    |      |                                                                                   |             |                                               
  |                    |      |                                                                                   |             |             +------ DMRT1A                                  fly               R1       
  |                    |      |                                                                                   |             +-------993.0-|                                               
  |                    |      |                                                                                   |                           +------ RTAg4                                   mosquito          R1       
  |                    |      |                                                                                   |                                               
  |                    |      |                                                                            +377.0-|             +-------------------- Tad1-1\_ACa                              fungus            Tad1       
  |                    |      |                                                                            |      |             |                                               
  |                    |      |                                                                            |      |      +622.0-|             +------ Tad1                                    fungus            Tad1       
  |                    |      |                                                                            |      |      |      |      +966.0-|                                               
  |                    |      |                                                                            |      |      |      |      |      +------ Tad1-3\_ACa                              fungus            Tad1       
  |                    |      |                                                                            |      |      |      +925.0-|                                               
  |                    |      |                                                                            |      +947.0-|             |      +------ Tad1-2\_ACa                              fungus            Tad1       
  |                    |      +----------------------------------------------------------------------616.0-|             |             +643.0-|                                               
  |                    |                                                                                   |             |                    +------ I-6\_AO                                  fungus            Tad1       
  |                    |                                                                                   |             |                                               
  |                    |                                                                                   |             |                    +------ MGR583                                  fungus            Tad1       
  |                    |                                                                                   |             +--------------996.0-|                                               
  |                    |                                                                                   |                                  +------ I-1\_AN                                  fungus            Tad1       
  |                    |                                                                                   |                                               
  |                    |                                                                                   |                           +------------- Baggins1\_Cis                            tunicate          Loa       
  |                    |                                                                                   +----------------------1000-|                                               
  |                    |                                                                                                               |      +------ Baggins-2\_NVi                           insect            Loa       
  |                    |                                                                                                               +714.0-|                                               
  |                    |                                                                                                                      +------ LOA                                     fly               Loa       
  |                    |                                               
  |                    |                                                                                                                      +------ RTEX-1\_CR                               green\_algae       RTEX       
  |                    |                                                                                                               +-1000-|                                               
  |                    |                                                                                                               |      +------ RTEX-4\_VC                               green\_algae       RTEX       
  |                    |                                                                                                        +693.0-|                                               
  |                    |                                                                                                        |      |      +------ RTEX-3\_VC                               green\_algae       RTEX       
  |                    |                                                                     +-----------------------------1000-|      +948.0-|                                               
  |                    |                                                                     |                                  |             +------ RTEX-1\_VC                               green\_algae       RTEX       
  |                    |                                                                     |                                  |                                               
  |                    |                                                                     |                                  +-------------------- RTEX-2\_VC                               green\_algae       RTEX       
  |                    |                                                                     |                                               
  |                    |                                                                     |                                                +------ RTEX-2\_NV                               cnidaria          RTEX       
  |                    |                                                                     |                                         +-1000-|                                               
  |                    |                                                                     |                    +---------------1000-|      +------ RTEX-3\_NV                               cnidaria          RTEX       
  |                    |                                                                     |                    |                    |                                               
  |                    |                                                                     |                    |                    +------------- RTEX-1\_NV                               cnidaria          RTEX       
  |                    |                                                              +772.0-|                    |                                               
  |             +610.0-|                                                              |      |                    |                           +------ RTEX-4\_BF                               amphioxus         RTEX       
  |             |      |                                                              |      |             +432.0-|                    +546.0-|                                               
  |             |      |                                                              |      |             |      |             +957.0-|      +------ RTEX-6\_BF                               amphioxus         RTEX       
  |             |      |                                                              |      |             |      |             |      |                                               
  |             |      |                                                              |      |             |      |      +451.0-|      +------------- RTEX-2\_BF                               amphioxus         RTEX       
  |             |      |                                                              |      |             |      |      |      |                                               
  |             |      |                                                              |      |      +995.0-|      |      |      |             +------ RTEX-5\_BF                               amphioxus         RTEX       
  |             |      |                                                              |      |      |      |      +675.0-|      +-------939.0-|                                               
  |             |      |                                                              |      |      |      |             |                    +------ RTEX-3\_BF                               amphioxus         RTEX       
  |             |      |                                                              |      |      |      |             |                                               
  |             |      |                                                              |      +967.0-|      |             +--------------------------- RTEX-4\_NV                               cnidaria          RTEX       
  |             |      |                                                              |             |      |                                               
  |             |      |                                                              |             |      +----------------------------------------- RTEX-1\_SK                               hemichordata      RTEX       
  |             |      |                                                              |             |                                               
  |             |      |                                                              |             +------------------------------------------------ RTEX-1\_BF                               amphioxus         RTEX       
  |             |      |                                                              |                                               
  |             |      |                                                              |                                                       +------ RTE-2\_BF                                amphioxus         RTE       
  |             |      |                                                              |                                                +879.0-|                                               
  |             |      |                                                              |                                         +228.0-|      +------ RTE                                     cow               RTE       
  |             |      |                                                              |                                         |      |                                               
  |             |      |                                                              |                           +-------190.0-|      +------------- Perere-3                                planaria          RTE       
  |             |      |                                                       +921.0-|                           |             |                                               
  |             |      |                                                       |      |                           |             |             +------ RTE-15\_BF                               amphioxus         RTE       
  |             |      |                                                       |      |                           |             +--------1000-|                                               
  |             |      |                                                       |      |                           |                           +------ RTE-14\_BF                               amphioxus         RTE       
  |             |      |                                                       |      |                           |                                               
  |             |      |                                                       |      |                           |                           +------ RTE-1\_DR                                fish              RTE       
  |             |      |                                                       |      |                    +-67.0-|                    +-1000-|                                               
  |             |      |                                                       |      |                    |      |                    |      +------ RTE-4\_NV                                cnidaria          RTE       
  |             |      |                                                       |      |                    |      |             +490.0-|                                               
  |             |      |                                                       |      |                    |      |             |      |      +------ RTE-3\_NV                                cnidaria          RTE       
  |             |      |                                                       |      |                    |      |      +-1000-|      +909.0-|                                               
  |             |      |                                                       |      |                    |      |      |      |             +------ RTE-1\_NV                                cnidaria          RTE       
  |             |      |                                                       |      |                    |      |      |      |                                               
  |             |      |                                                       |      |             +292.0-|      +-82.0-|      +-------------------- RTE-5\_NV                                cnidaria          RTE       
  |             |      |                                                       |      |             |      |             |                                               
  |             |      |                                                       |      |             |      |             |                    +------ Expander1\_Cis                           tunicate          RTE       
  |             |      |                                                +361.0-|      |             |      |             +--------------507.0-|                                               
  |             |      |                                                |      |      |             |      |                                  +------ RTE-1\_AG                                mosquito          RTE       
  |             |      |                                                |      |      |      +824.0-|      |                                               
  |             |      |                                                |      |      |      |      |      |                                  +------ SR2                                     planaria          RTE       
  |             |      |                                                |      |      |      |      |      +-----------------------------1000-|                                               
  |             |      |                                                |      |      |      |      |                                         +------ RTE\_SJ                                  planaria          RTE       
  |             |      |                                                |      |      |      |      |                                               
  |             |      |                                                |      |      +666.0-|      |                                  +------------- Expander                                fish              RTE       
  |             |      |                                                |      |             |      +----------------------------380.0-|                                               
  |             |      |                                                |      |             |                                         |      +------ RTE-1\_BF                                amphioxus         RTE       
  |             |      |                                                |      |             |                                         +511.0-|                                               
  |             |      |                                                |      |             |                                                +------ RTE1\_ZM                                 plant             RTE       
  |             |      |                                                |      |             |                                               
  |             |      |                                                |      |             +------------------------------------------------------- RTE-1                                   nematoda          RTE       
  |             |      |                                                |      |                                               
  |             |      |                                                |      |                                                              +------ RTETP-1\_FC                              diatom            RTETP       
  |             |      |                                                |      |                                                       +985.0-|                                               
  |             |      |                                         +369.0-|      +--------------------------------------------------1000-|      +------ RTE-1\_TP                                diatom            RTETP       
  |             |      |                                         |      |                                                              |                                               
  |             |      |                                         |      |                                                              +------------- RTETP-1\_PT                              diatom            RTETP       
  |             |      |                                         |      |                                               
  |             |      |                                         |      |                                                              +------------- Proto2-8\_CS1                            annelida          Proto2       
  |             |      |                                         |      |                                                       +427.0-|                                               
  |             |      |                                         |      |                                                       |      |      +------ Proto2-1\_SK                             hemichordata      Proto2       
  |             |      |                                         |      |                                         +--------1000-|      +569.0-|                                               
  |             |      |                                         |      |                                         |             |             +------ Proto2-7\_CS1                            annelida          Proto2       
  |             |      |                                         |      |                                         |             |                                               
  |             |      |                                         |      |                                         |             +-------------------- Proto2-1\_BF                             amphioxus         Proto2       
  |             |      |                                         |      |                                  +990.0-|                                               
  |             |      |                                         |      |                                  |      |      +--------------------------- Proto2-3\_CS1                            annelida          Proto2       
  |             |      |                                         |      |                                  |      |      |                                               
  |             |      |                                         |      |                                  |      |      |             +------------- Proto2-5\_CS1                            annelida          Proto2       
  |             |      |                                         |      |                                  |      +-1000-|      +508.0-|                                               
  |             |      +-----------------------------------389.0-|      |                                  |             |      |      |      +------ Proto2-2\_CS1                            annelida          Proto2       
  |             |                                                |      |                                  |             |      |      +892.0-|                                               
  |             |                                                |      +----------------------------988.0-|             +350.0-|             +------ Proto2-6\_CS1                            annelida          Proto2       
  |             |                                                |                                         |                    |                                               
  |             |                                                |                                         |                    |             +------ Proto2-1\_CS1                            annelida          Proto2       
  |      +248.0-|                                                |                                         |                    +--------1000-|                                               
  |      |      |                                                |                                         |                                  +------ Proto2-4\_CS1                            annelida          Proto2       
  |      |      |                                                |                                         |                                               
  |      |      |                                                |                                         |                                  +------ Proto2-1\_HM                             cnidaria          Proto2       
  |      |      |                                                |                                         +-----------------------------1000-|                                               
  |      |      |                                                |                                                                            +------ Proto2-2\_HM                             cnidaria          Proto2       
  |      |      |                                                |                                               
  |      |      |                                                |                                                                     +------------- IRTE-7\_OR                               fungus            IRTE       
  |      |      |                                                |                                                              +937.0-|                                               
  |      |      |                                                |                                                              |      |      +------ IRTE-8\_OR                               fungus            IRTE       
  |      |      |                                                |                                                              |      +412.0-|                                               
  |      |      |                                                +---------------------------------------------------------1000-|             +------ IRTE-3\_OR                               fungus            IRTE       
  |      |      |                                                                                                               |                                               
  |      |      |                                                                                                               |             +------ IRTE-4\_OR                               fungus            IRTE       
  |      |      |                                                                                                               +-------789.0-|                                               
  |      |      |                                                                                                                             +------ IRTE-1\_OR                               fungus            IRTE       
  |      |      |                                               
  |      |      |                                                                                                                             +------ L1-1\_XT                                 frog              L1       
  |      |      |                                                                                                 +---------------------997.0-|                                               
  |      |      |                                                                                                 |                           +------ L1-11\_XT                                frog              L1       
  |      |      |                                                                                                 |                                               
  |      |      |                                                                                                 |             +-------------------- L1                                      human             L1       
  |      |      |                                                                                          +745.0-|             |                                               
  |      |      |                                                                                          |      |      +234.0-|             +------ L1-39\_XT                                frog              L1       
  |      |      |                                                                                          |      |      |      |      +660.0-|                                               
  |      |      |                                                                                          |      |      |      +197.0-|      +------ L1-34\_XT                                frog              L1       
  |      |      |                                                                                          |      +755.0-|             |                                               
  |      |      |                                                                                   +978.0-|             |             +------------- L1-38\_XT                                frog              L1       
  |      |      |                                                                                   |      |             |                                               
  |      |      |                                                                                   |      |             |                    +------ L1-1\_DR                                 fish              L1       
  |      |      |                                                                                   |      |             +--------------580.0-|                                               
  |      |      |                                                                                   |      |                                  +------ Swimmer                                 fish              L1       
  |      |      |                                                                                   |      |                                               
  |      |      |                                                                                   |      +----------------------------------------- L1-40\_XT                                frog              L1       
  |      |      |                                                                                   |                                               
  |      |      |                                                                                   |                           +-------------------- L1-56\_XT                                frog              Tx1       
  |      |      |                                                                            +414.0-|                    +987.0-|                                               
  |      |      |                                                                            |      |                    |      |      +------------- L1-55\_XT                                frog              Tx1       
  |      |      |                                                                            |      |                    |      +482.0-|                                               
  |      |      |                                                                            |      |                    |             |      +------ KenoDr1                                 fish              Tx1       
  |      |      |                                                                            |      |                    |             +-1000-|                                               
  |      |      |                                                                            |      |             +888.0-|                    +------ KenoFr1                                 fish              Tx1       
  |      |      |                                                                            |      |             |      |                                               
  |      |      |                                                                            |      |             |      |                    +------ KoshiTn1                                fish              Tx1       
  |      |      |                                                                            |      |             |      |             +997.0-|                                               
  |      |      |                                                                     +428.0-|      |             |      |             |      +------ Tx1\_XT                                  frog              Tx1       
  |      |      |                                                                     |      |      +-------761.0-|      +-------862.0-|                                               
  |      |      |                                                                     |      |                    |                    |      +------ Tx1-2\_NV                                cnidaria          Tx1       
  |      |      |                                                                     |      |                    |                    +999.0-|                                               
  |      |      |                                                                     |      |                    |                           +------ Tx1-1\_NV                                cnidaria          Tx1       
  +124.0-|      |                                                                     |      |                    |                                               
         |      |                                                                     |      |                    +---------------------------------- L1-3\_Cis                                tunicate          Tx1       
         |      |                                                                     |      |                                               
         |      |                                                                     |      |                                                +------ L1-2\_RO                                 fungus            L1       
         |      |                                                                     |      |                                         +696.0-|                                               
         |      |                                                              +164.0-|      +------------------------------------1000-|      +------ L1-1\_RO                                 fungus            L1       
         |      |                                                              |      |                                                |                                               
         |      |                                                              |      |                                                +------------- L1-3\_RO                                 fungus            L1       
         |      |                                                              |      |                                               
         |      |                                                              |      |                                                       +------ SHALINE16\_MT                            plant             L1       
         |      |                                                              |      |                                                +804.0-|                                               
         |      |                                                              |      |                                         +777.0-|      +------ ATLINE1                                 plant             L1       
         |      |                                                              |      |                                         |      |                                               
         |      |                                                       +110.0-|      |                                  +994.0-|      +------------- CIN4E\_ZM                                plant             L1       
         |      |                                                       |      |      |                                  |      |                                               
         |      |                                                       |      |      +----------------------------492.0-|      +-------------------- SHALINE14\_MT                            plant             L1       
         |      |                                                       |      |                                         |                                               
         |      |                                                       |      |                                         +--------------------------- Oryzapunctata3177902                           
         |      |                                                       |      |                                               
         |      |                                                       |      |                                                              +------ L1-1\_VC                                 green\_algae       L1       
         |      |                                                +139.0-|      |                                                       +-1000-|                                               
         |      |                                                |      |      |                                                       |      +------ L1-4\_VC                                 green\_algae       L1       
         |      |                                                |      |      +-------------------------------------------------962.0-|                                               
         |      |                                                |      |                                                              |      +------ Zepp                                    green\_algae       L1       
         |      |                                                |      |                                                              +-1000-|                                               
         |      |                                                |      |                                                                     +------ L1-1\_CR                                 green\_algae       L1       
         |      +------------------------------------------849.0-|      |                                               
         |                                                       |      |                                                                     +------ TDD3                                    protozoa          L1       
         |                                                       |      +---------------------------------------------------------------990.0-|                                               
         |                                                       |                                                                            +------ DRE                                     protozoa          L1       
         |                                                       |                                               
         |                                                       |                                                                            +------ Ylli                                    fungus            L1       
         |                                                       +----------------------------------------------------------------------418.0-|                                               
         |                                                                                                                                    +------ Zorro                                   fungus            L1       
         |                                               
         |                                                                                                                                    +------ Ambal-2\_TP                              diatom            Ambal       
         |                                                                                                                             +-1000-|                                               
         |                                                                                                                      +661.0-|      +------ Ambal-1\_TP                              diatom            Ambal       
         |                                                                                                                      |      |                                               
         |                                                                                   +-----------------------------1000-|      +------------- Ambal-3\_FC                              diatom            Ambal       
         |                                                                                   |                                  |                                               
         |                                                                                   |                                  |             +------ Ambal-2\_FC                              diatom            Ambal       
         |                                                                                   |                                  +-------991.0-|                                               
         |                                                                                   |                                                +------ Ambal-1\_FC                              diatom            Ambal       
         |                                                                                   |                                               
         |                                                                                   |                                         +------------- Cre-1\_FC                                diatom            CRE       
         |                                                                                   |                                  +-1000-|                                               
         |                                                                                   |                                  |      |      +------ Cre-1\_HM                                cnidaria          CRE       
         |                                                                                   |                                  |      +993.0-|                                               
         |                                                                                   |                           +332.0-|             +------ Cre-1\_NV                                cnidaria          CRE       
         |                                                                                   |                           |      |                                               
         +-----------------------------------------------------------------------------438.0-|                           |      |             +------ Cre-2\_BM                                insecta           CRE       
                                                                                             |                    +371.0-|      +--------1000-|                                               
                                                                                             |                    |      |                    +------ Cre-1\_BM                                insecta           CRE       
                                                                                             |                    |      |                                               
                                                                                             |                    |      +--------------------------- Cnl1                                    fungus            CRE       
                                                                                             |             +594.0-|                                               
                                                                                             |             |      |                           +------ CRE1                                    protozoa          CRE       
                                                                                             |             |      |                    +729.0-|                                               
                                                                                             |             |      |                    |      +------ CRE2                                    protozoa          CRE       
                                                                                             |      +472.0-|      +---------------1000-|                                               
                                                                                             |      |      |                           |      +------ SLACS                                   protozoa          CRE       
                                                                                             |      |      |                           +-1000-|                                               
                                                                                             |      |      |                                  +------ CZAR                                    protozoa          CRE       
                                                                                             +563.0-|      |                                               
                                                                                                    |      +----------------------------------------- Cre-1\_MB                                choanoflag.       CRE       
                                                                                                    |                                               
                                                                                                    |                                         +------ GilM                                    protozoa          CRE       
                                                                                                    +-----------------------------------997.0-|                                               
                                                                                                                                              +------ GilD                                    protozoa          CRE       
                                               
                                               
  
Link of : A fragment of your sequence used in the multiple alignment  
  
Link of : Multiple Alignment  
  
Link of : Tree without Bootstrap  
  
Link of : SVG file created by Dendroscope  
  
