# Supplementary material for: Sample Sequence Analysis Uncovers Recurrent Horizontal Transfers of Transposable Elements among Grasses
Source: Mol Biol Evol. 2021 May 8;38(9):3664–75. doi: 10.1093/molbev/msab133 (PMC8382918; doi:10.1093/molbev/msab133)
Supplement: msab133_Supplementary_Data [file msab133_supplementary_data.zip › Supplementary_Figures.pdf]

Sample sequence analysis uncovers recurrent horizontal transfers of transposable elements among grasses

Minkyu Park[1], Pascal-Antoine Christin[2], and Jeffrey L. Bennetzen[1, 3*]

[1] Department of Genetics, University of Georgia, Athens, GA 30602, USA

[2] Department of Animal and Plant Sciences, University of Sheffield, Sheffield S10 TN, UK

[3] State Key Laboratory of Tea Plant Biology and Utilization, Anhui Agricultural University, 230036 Hefei, China

[*] To whom correspondence should be addressed. E-mail: maize@uga.edu

Running title: Recurrent horizontal transfer among grasses

Keywords: genome evolution, horizontal transfer, *Oryza*, panicoid grasses, Poaceae

SUPPLEMENTARY FIGURES

Supplementary FIG. S1. Phylogenetic tree of 19 panicoids and the size of low-depth sample sequences.

A phylogenetic tree of all 19 panicoids used in the LTR-retrotransposon-targeted HT investigation. The species in the same genus are marked with bold names. The size of the low-depth sample sequences of each species is presented by a black bar graph with numbers on the right panel. The estimated genome size of the panicoids is presented by a grey bar graph with the numbers. The 'ND' indicates not determined.

Supplementary FIG. S2. HT of LTR-retrotransposons in each cluster.

The top panel depicts the horizontal transfer of LTR-retrotransposons between panicoid and *Oryza* species. The species pair that has the horizontal transfer event is linked with a red line and marked with red names. The top-left panel exhibits the activity history of LTR-retrotransposons in each panicoid species. The phylogenetic tree of the 19 panicoid species is depicted at the left end. The top-right panel provides an identity histogram between the RT sequence of horizontally transferred LTR-retrotransposons and their homologous copies in each *Oryza* species. The phylogenetic tree of the 11 *Oryza* species is depicted at the right. Copy numbers are shown at the left end of each cell. The bottom panel exhibits the evidence for the HT of LTR-retrotransposon. The histogram at the bottom-left panel represents an estimated speciation point. The peak point is considered as a speciation point and indicated with a vertical dotted red line with the degree of sequence identity indicated at the top. The bottom-right panel shows the identity of the horizontally transferred LTR-retrotransposon between panicoid and *Oryza* species. The rice genome contig is depicted by a horizontal red line and the corresponding hits of individual reads from panicoid sample sequences are depicted by vertical bars. The height and color intensity of the vertical bars are proportional to the degree of identity. The speciation point is depicted by a horizontal dotted red line.

Supplementary FIG. S3. Phylogenetic analysis of HT cases.

RTs in each cluster containing HTs and its homologs in recipient *Oryza* species are used in constructing phylogenetic trees. The RTs of *Echinochloa* species are depicted with empty diamond and *Oryza* species are with filled diamond. The RTs of horizontally transferred LTR-retrotransposons are indicated by empty red arrow.

Supplementary FIG. S4. Comparative analysis of sequence contigs containing

horizontally transferred LTR-retrotransposons.

In the upper left panel, the horizontal red bar indicates the sequence contig containing the horizontally transferred LTR-retrotransposon. Horizontal yellow bars indicate the collinear sequences among *Oryza* species and each species name is marked at the right end of each bar. Vertical bars indicate the position and identity of mapped panicoid sequences. White and grey boxes in each horizontal bar indicate exons and introns of annotated genes. Blue boxes indicate annotated repeat sequences. Domains of LTR-retrotransposons are depicted by grey, pink, orange, green, and light blue boxes for a gag, aspartic protease, integrase, reverse transcriptase, and RNase H, respectively. Similar regions between sequence contigs are depicted by dark green or black shading between bars. The right panel presents dot-plot analysis between the sequence contig harboring the horizontally transferred LTR-retrotransposons and its orthologs. LTR sequences are indicated by red arrows and junctions of the insertion site are indicated by red boxes. The sequences of the junctions are shown in the lower-left panel. ‘TG’ and ‘CA’ sequences at the end of the LTR sequence are marked with red letters and the terminal site duplications are marked with green letters.

Supplementary FIG. S5. Sequence alignments of the junctions of the horizontally transferred LTR-retrotransposon.

Raw reads of *O. sativa* (SRA ID: ERX3148290) are aligned to the junctions of the HT case of CL102 (*O. sat.* – *C. pil.*). The “TG……CA” motif of LTR sequences and terminal site duplications are marked with red and green letters, respectively. The LTR sequence is marked with orange letters.

Supplementary FIG. S6. Comparative analysis of the paralogous copies of the horizontally transferred LTR-retrotransposons in the genome of *O. sativa*.

Four *O. sativa* sequence contigs harboring the horizontally transferred LTR-retrotransposons are compared (horizontal bars). The explanation of the figure is same as that of supplementary fig. S2

Supplementary FIG. S7. Phylogenetic tree of *Echinochloa* and *Oryza* species

Phylogenetic trees of nine *Echinochloa* and ten *Oryza* species used in the non-targeted

investigation of HTs. The numbers in triangles in the Poaceae phylogenetic tree indicate the number of species in each subfamily.

Supplementary FIG. S8. All horizontal transfer cases by non-targeted investigation

Evidence of all 165 HT cases. The horizontal red bars indicate *Oryza* target sequences harboring the HT elements. Vertical bars indicate mapped *Echinochloa* sample sequences. The height and color represent the identity of the mapped sequence. White and grey boxes in each horizontal bar indicate exons and introns of annotated genes. Blue and black boxes indicate annotated LTR-retrotransposons and DNA transposons. Domains of LTR-retrotransposons are depicted by grey, pink, orange, green, and light blue boxes, representing gag, aspartic protease, integrase, reverse transcriptase, and RNase H, respectively.

Supplementary FIG. S1

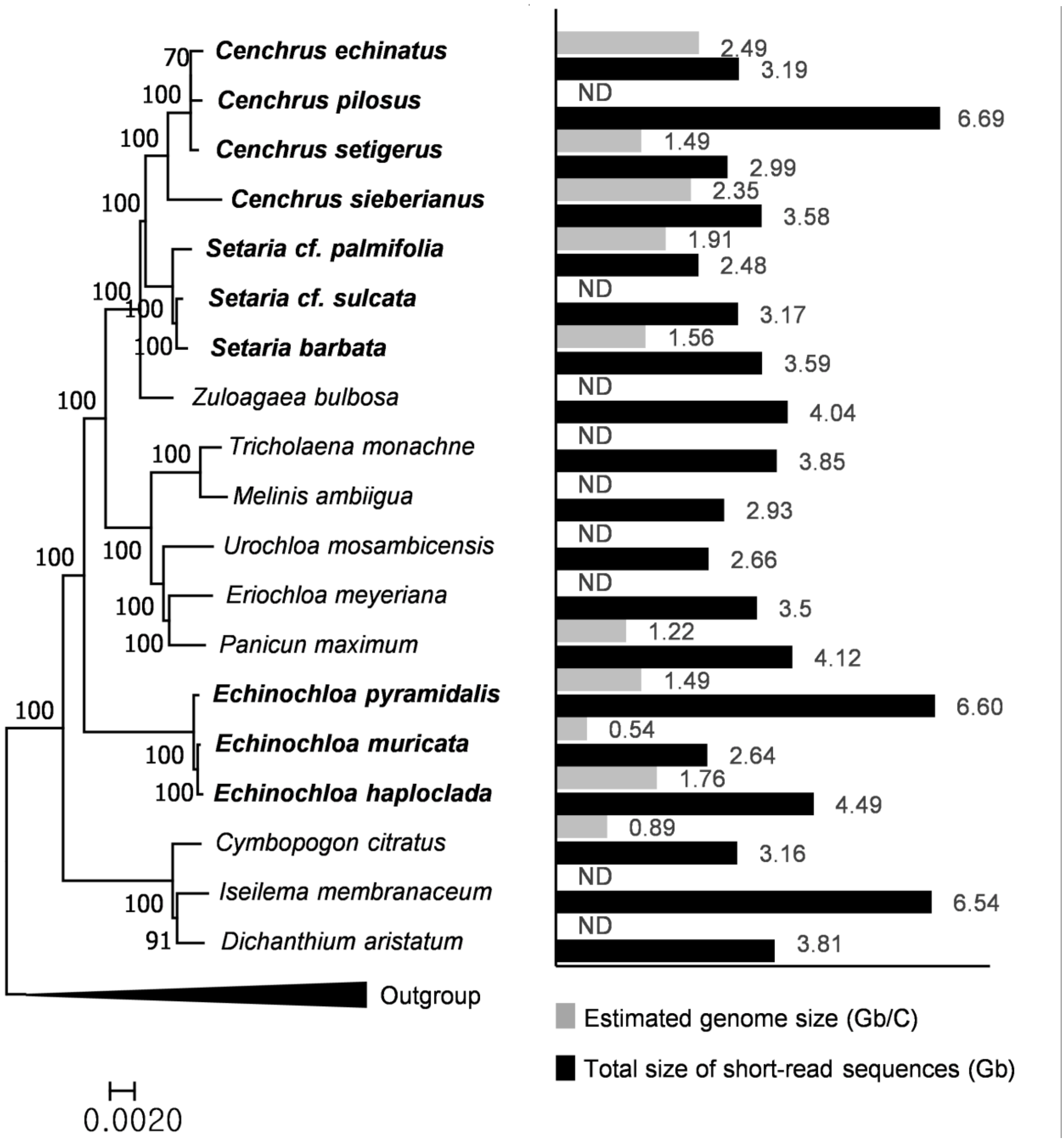

Supplementary FIG. S2

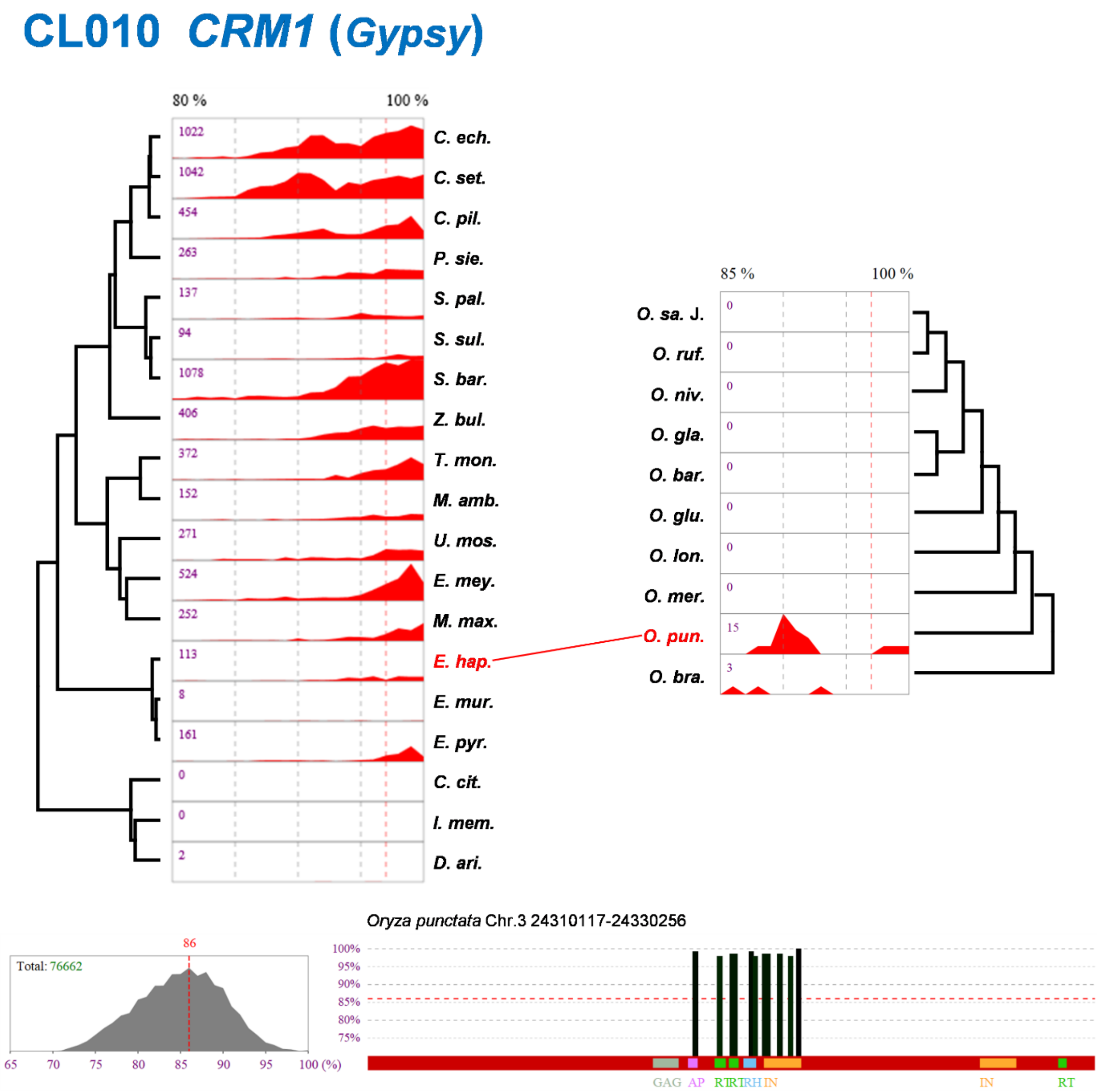

CL010 *CRM1* (*Gypsy*)

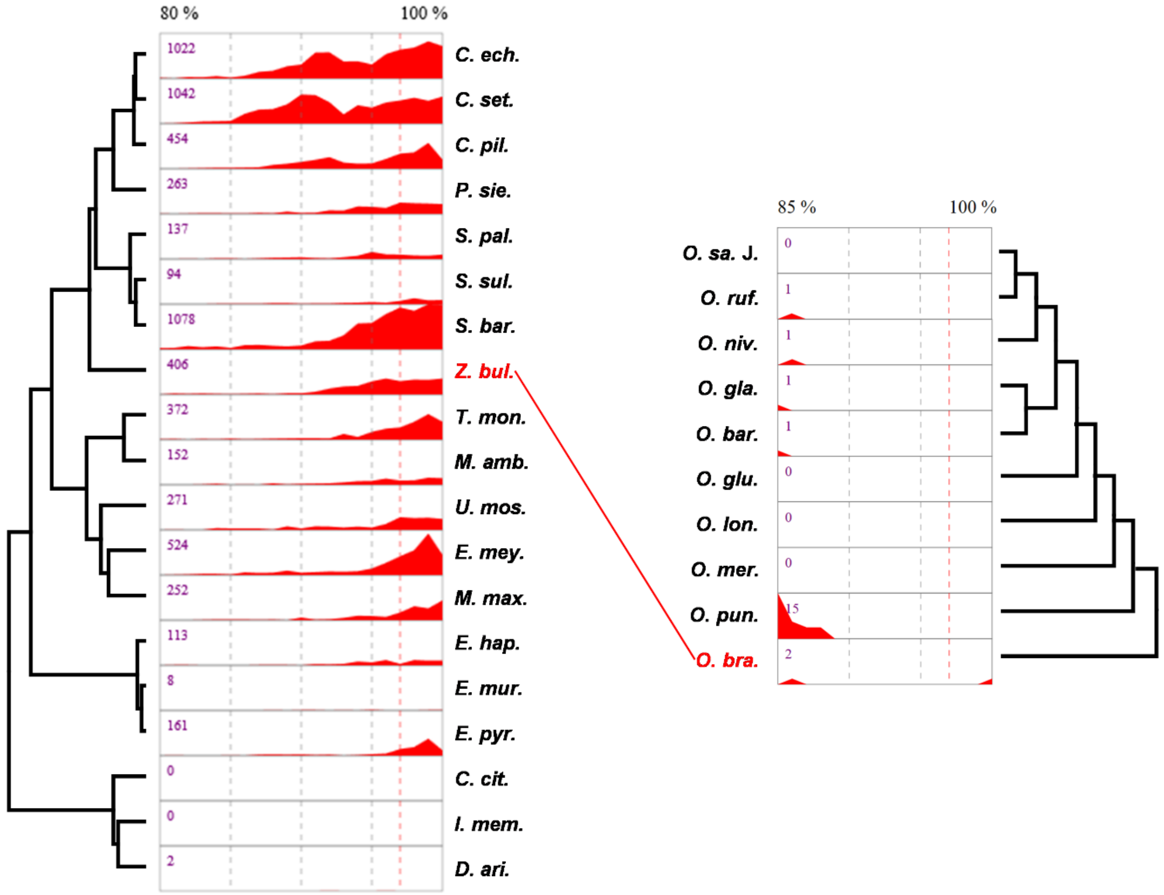

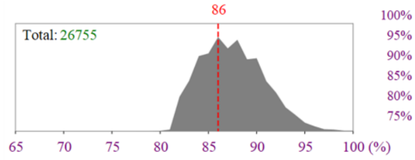

Oryza brachyantha JH592243.1 3480-6416

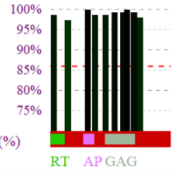

CL025 Unclassified (*Copia*)

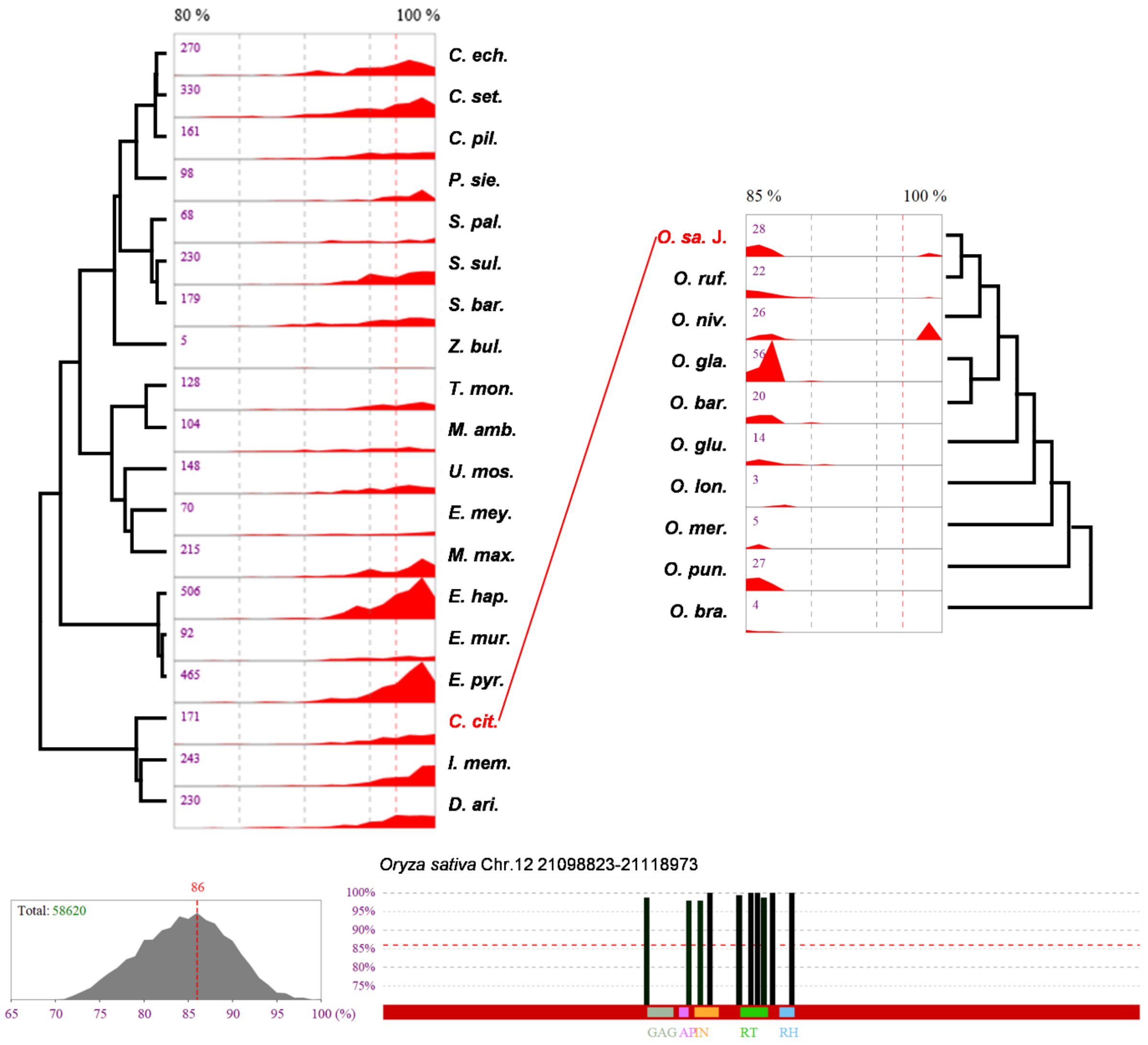

CL025 Unclassified (*Copia*)

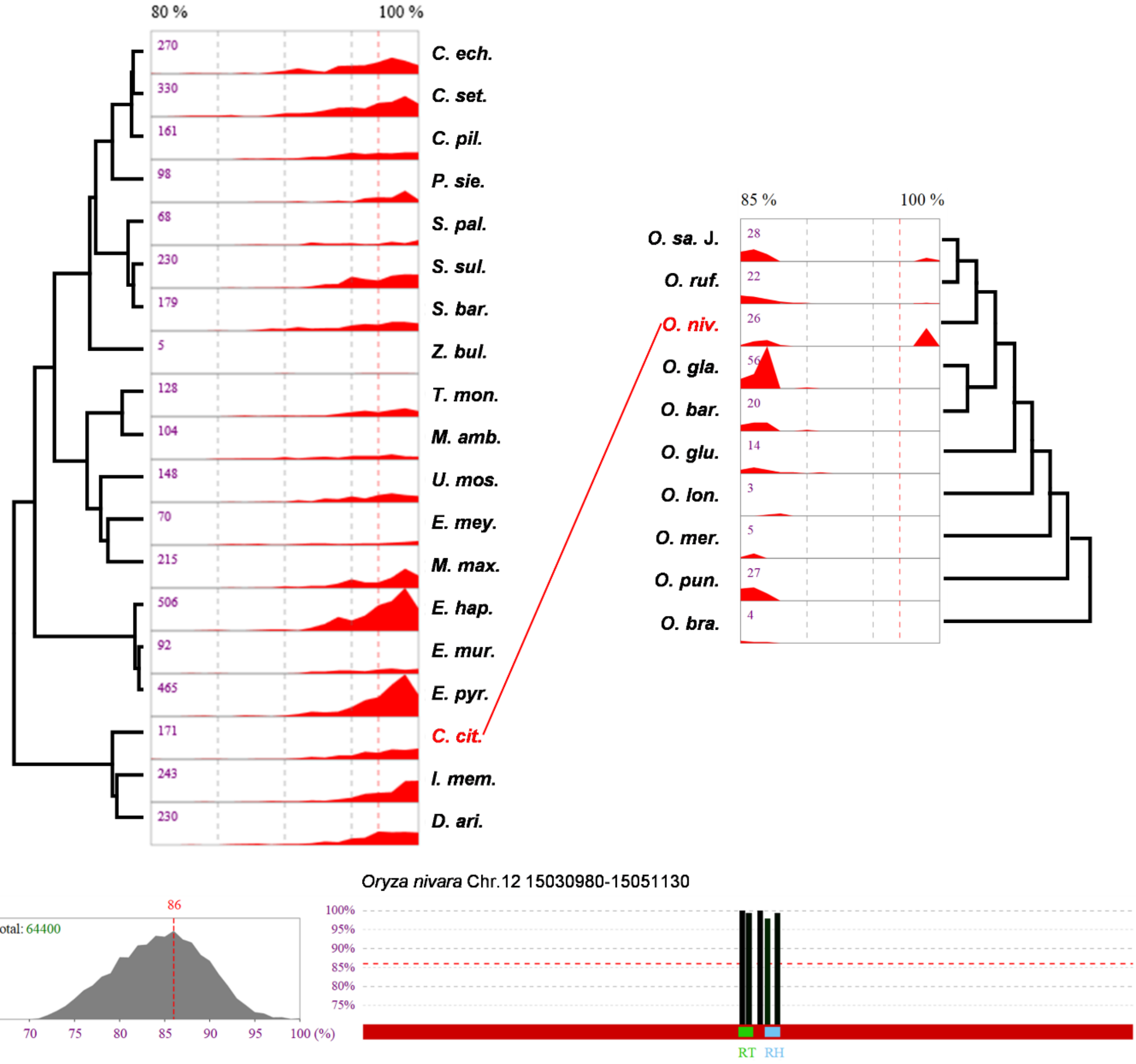

CL102 *Lusi* (*Copia*)

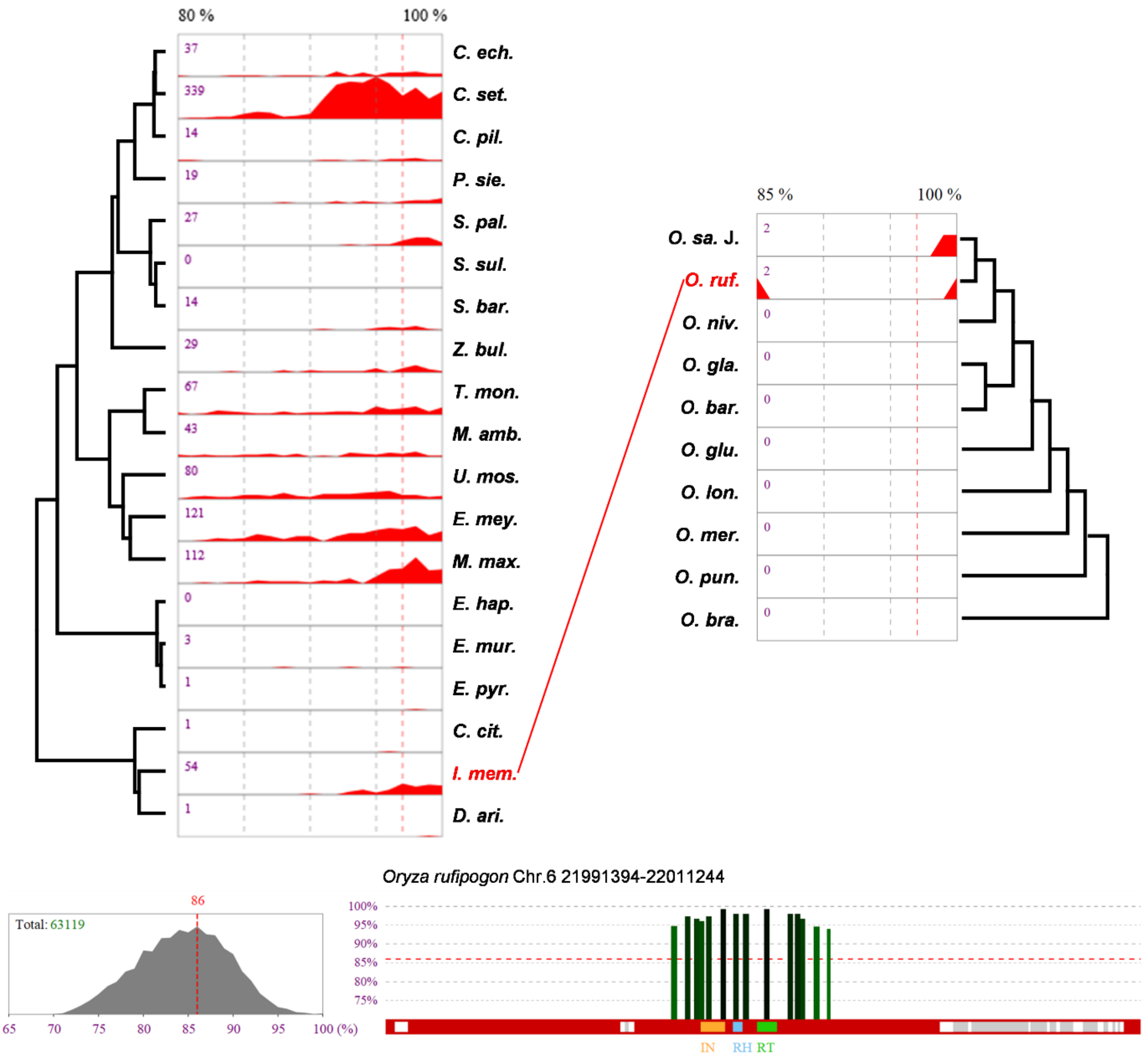

CL102 *Lusi* (*Copia*)

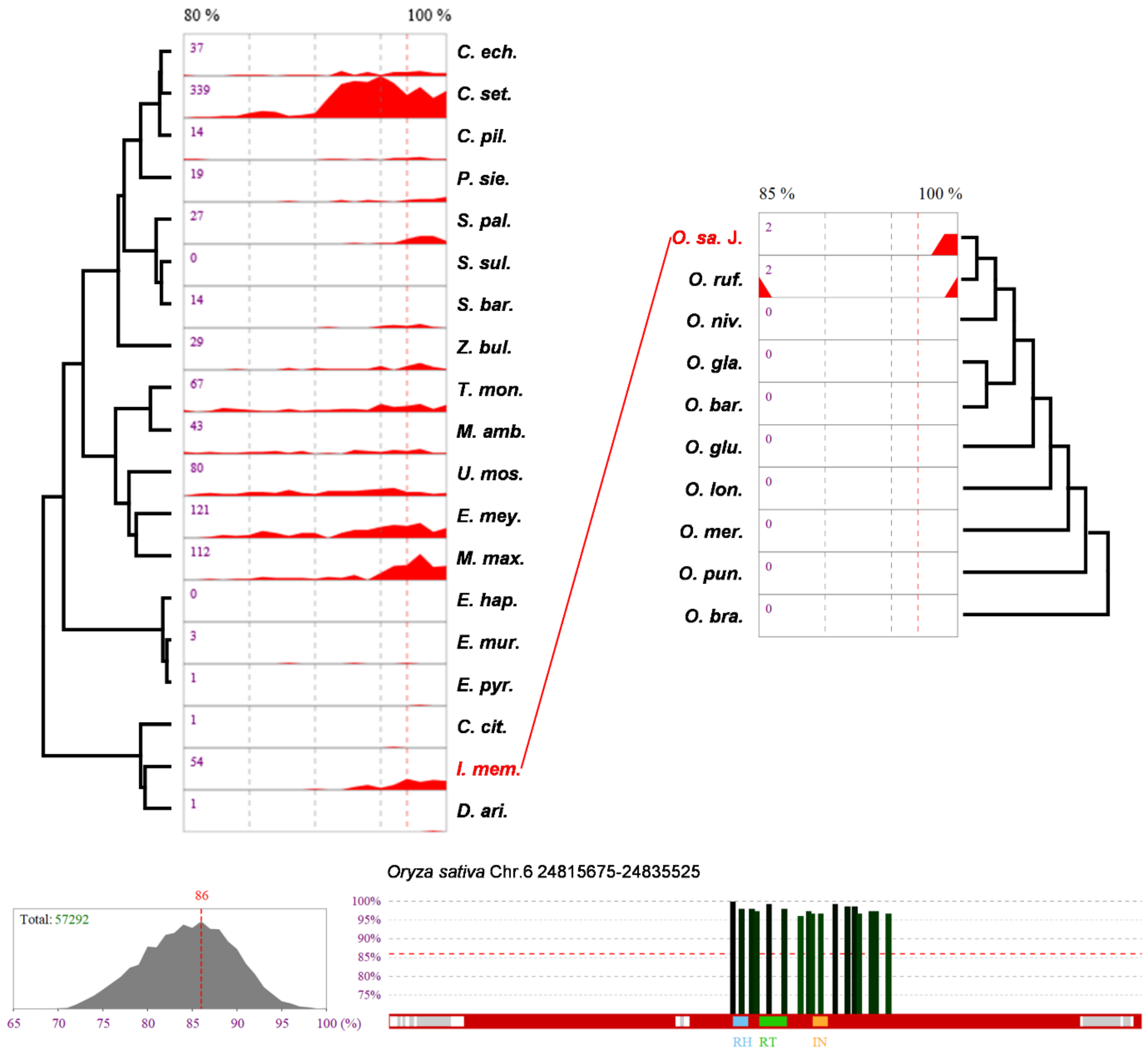

CL102 *Lusi* (*Copia*)

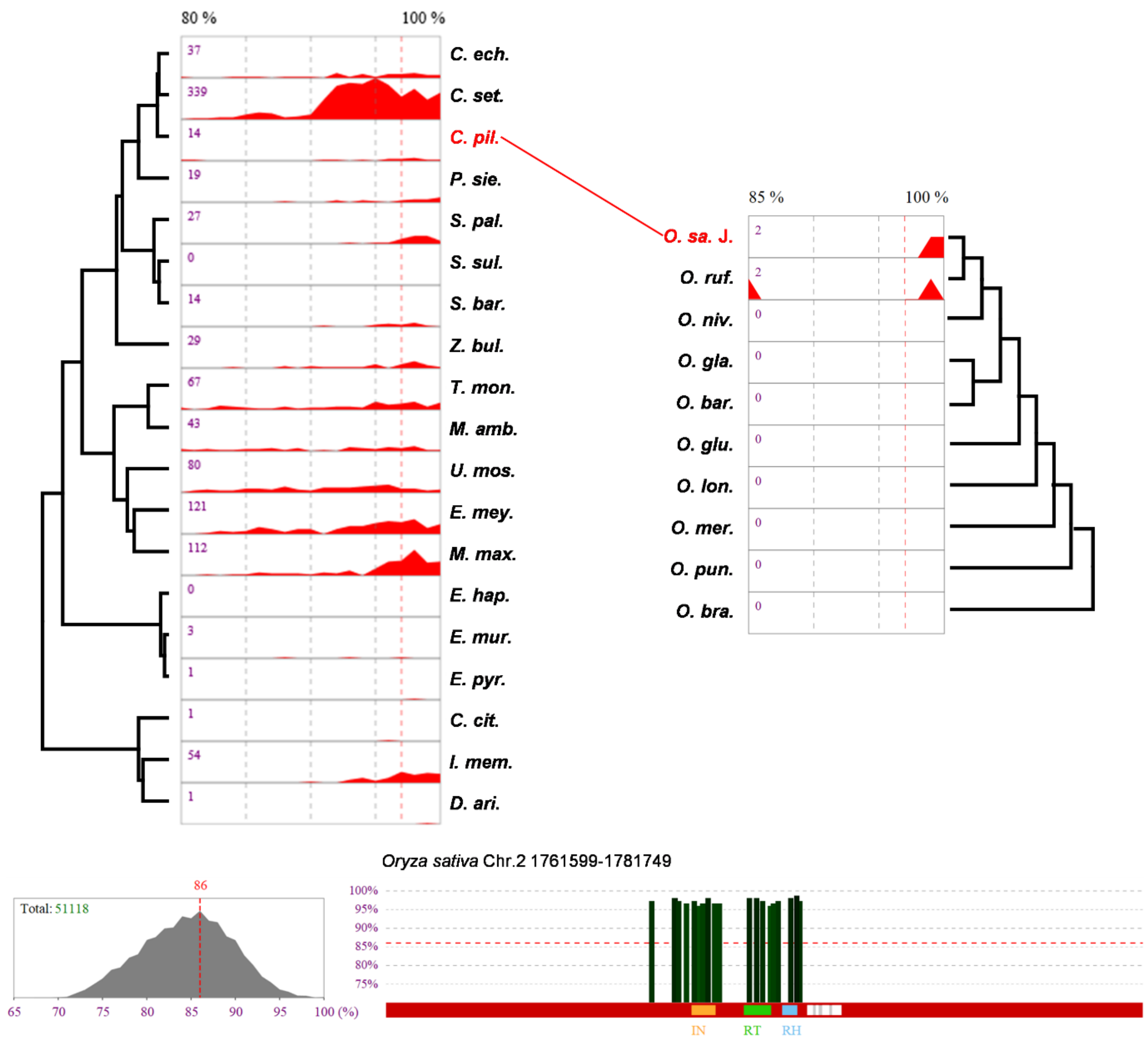

CL112 *Debeh* (*Copia*)

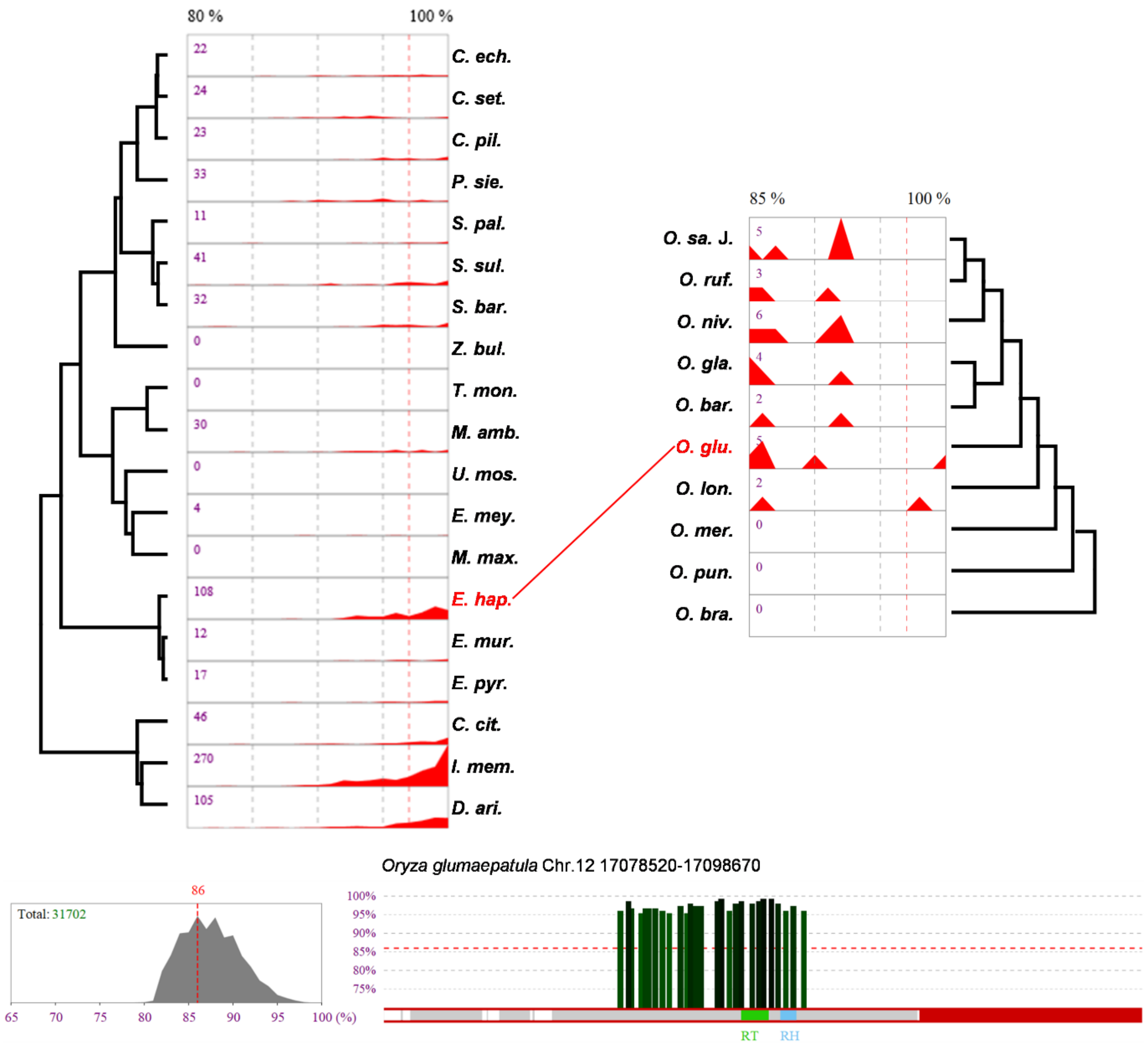

CL129 *Wihov* (*Gypsy*)

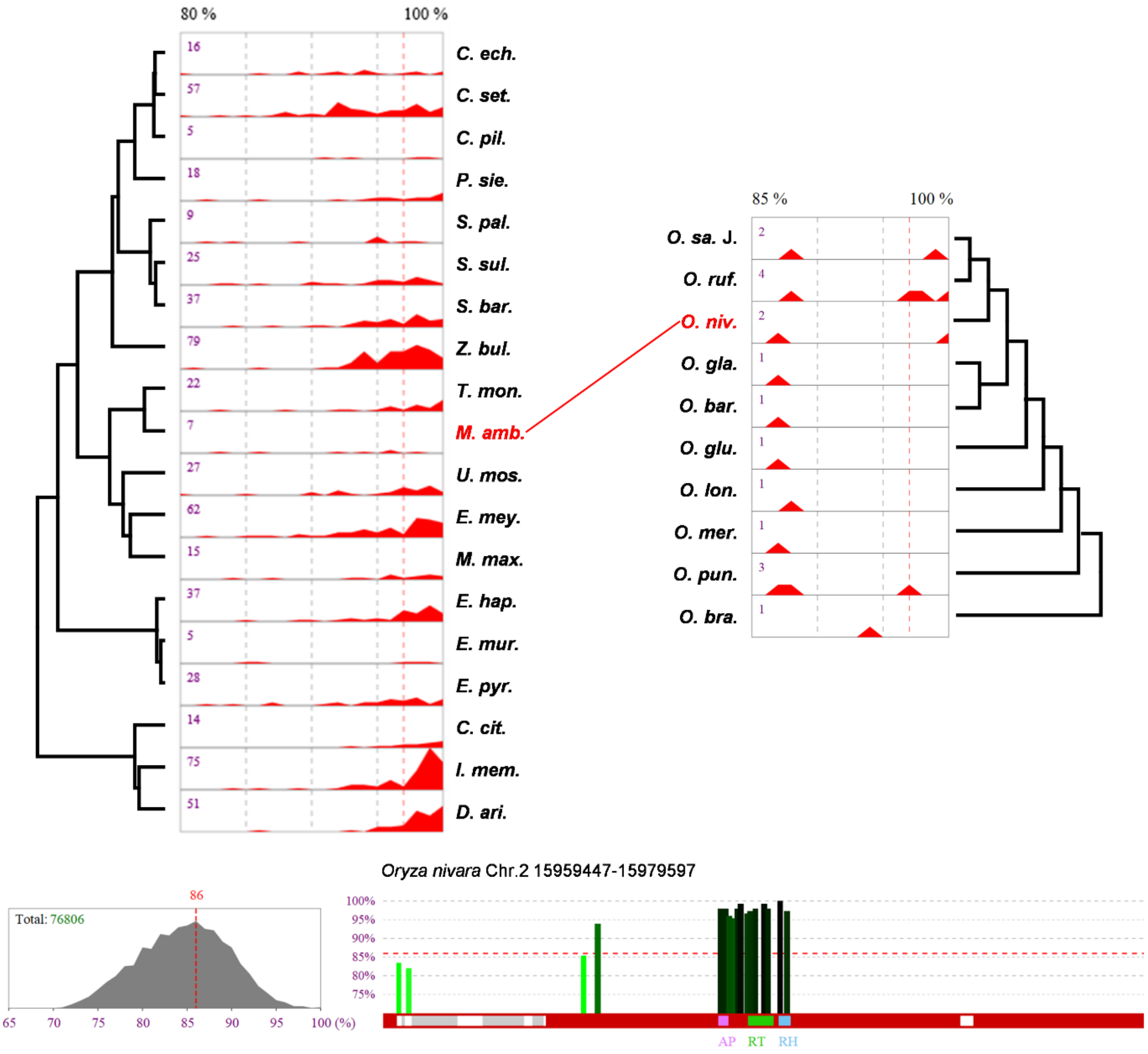

CL129 *Wihov* (*Gypsy*)

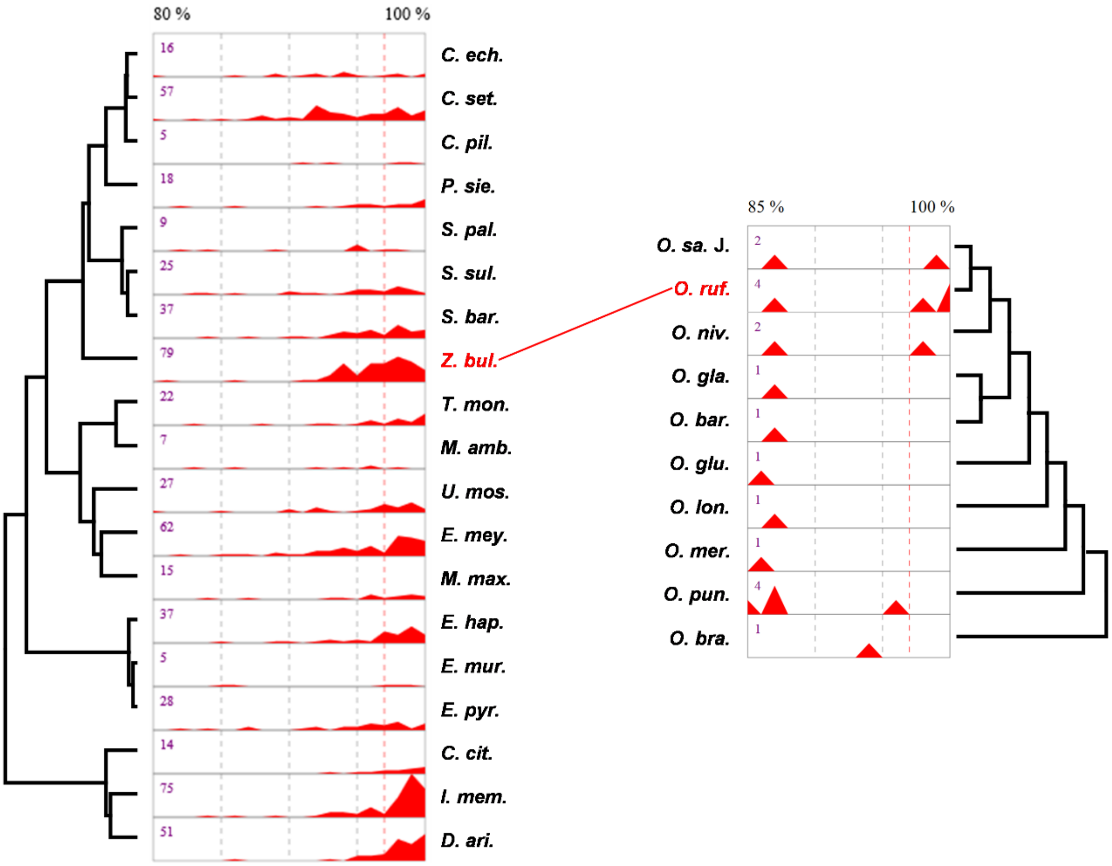

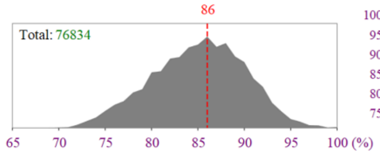

Oryza rufipogon Chr.10 7047133-7067282

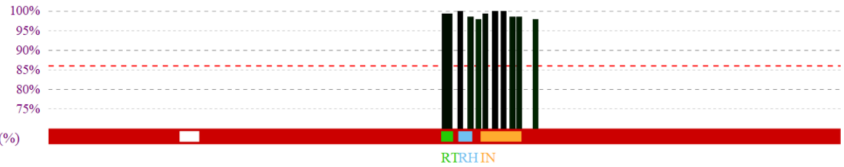

CL129 *Wihov* (*Gypsy*)

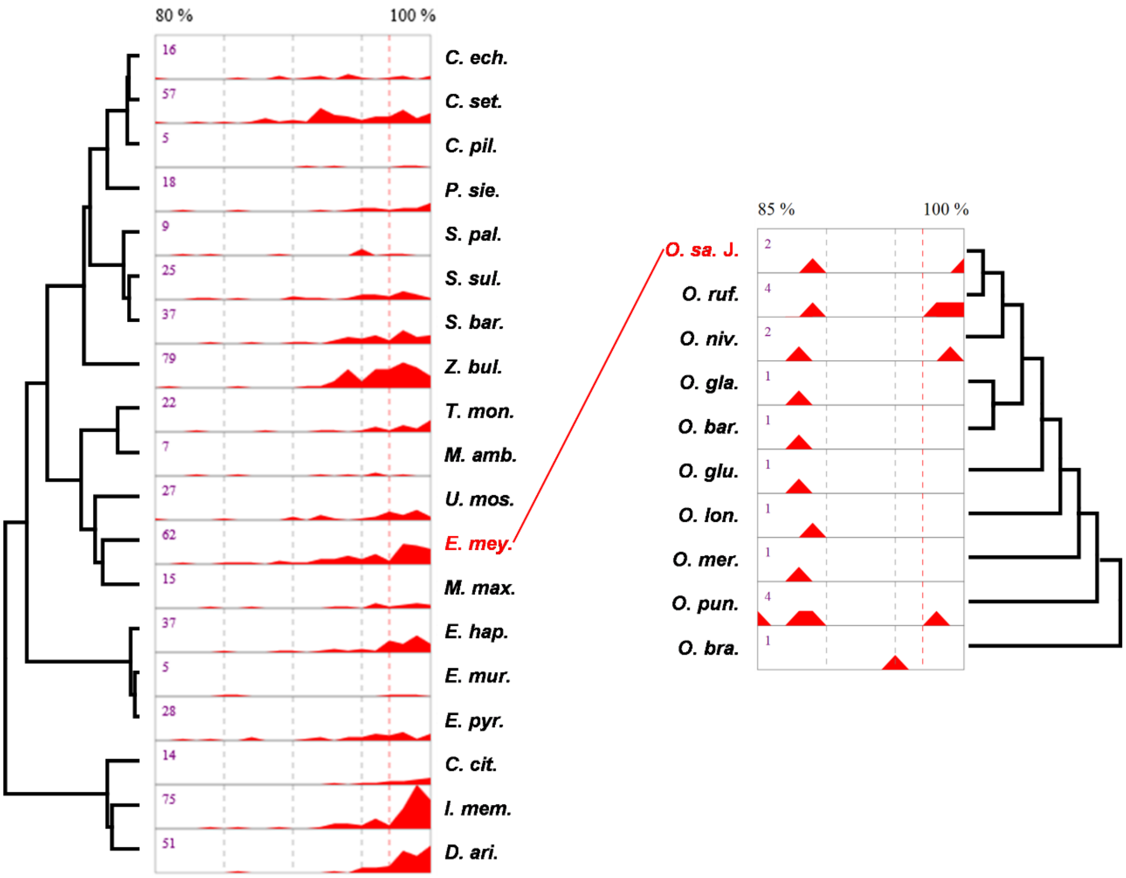

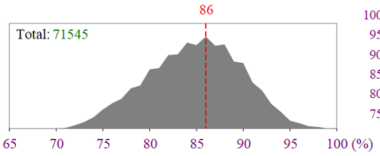

Oryza sativa Chr.6 15062703-15082553

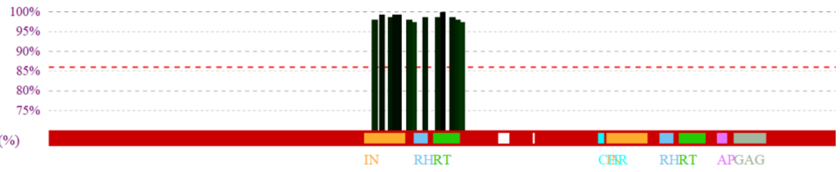

CL148 *Donuil* (*Copia*)

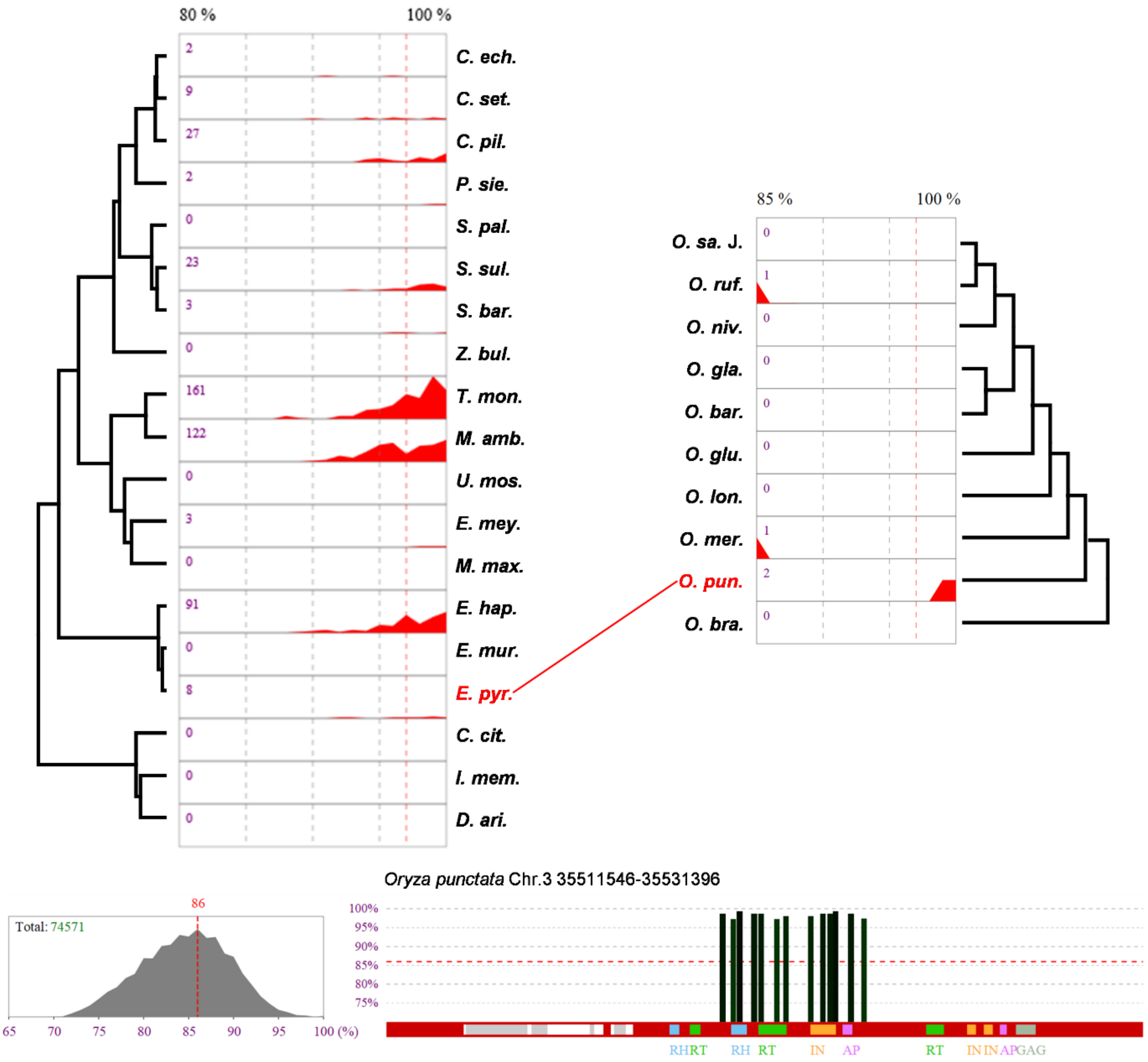

CL148 *Donuil* (*Copia*)

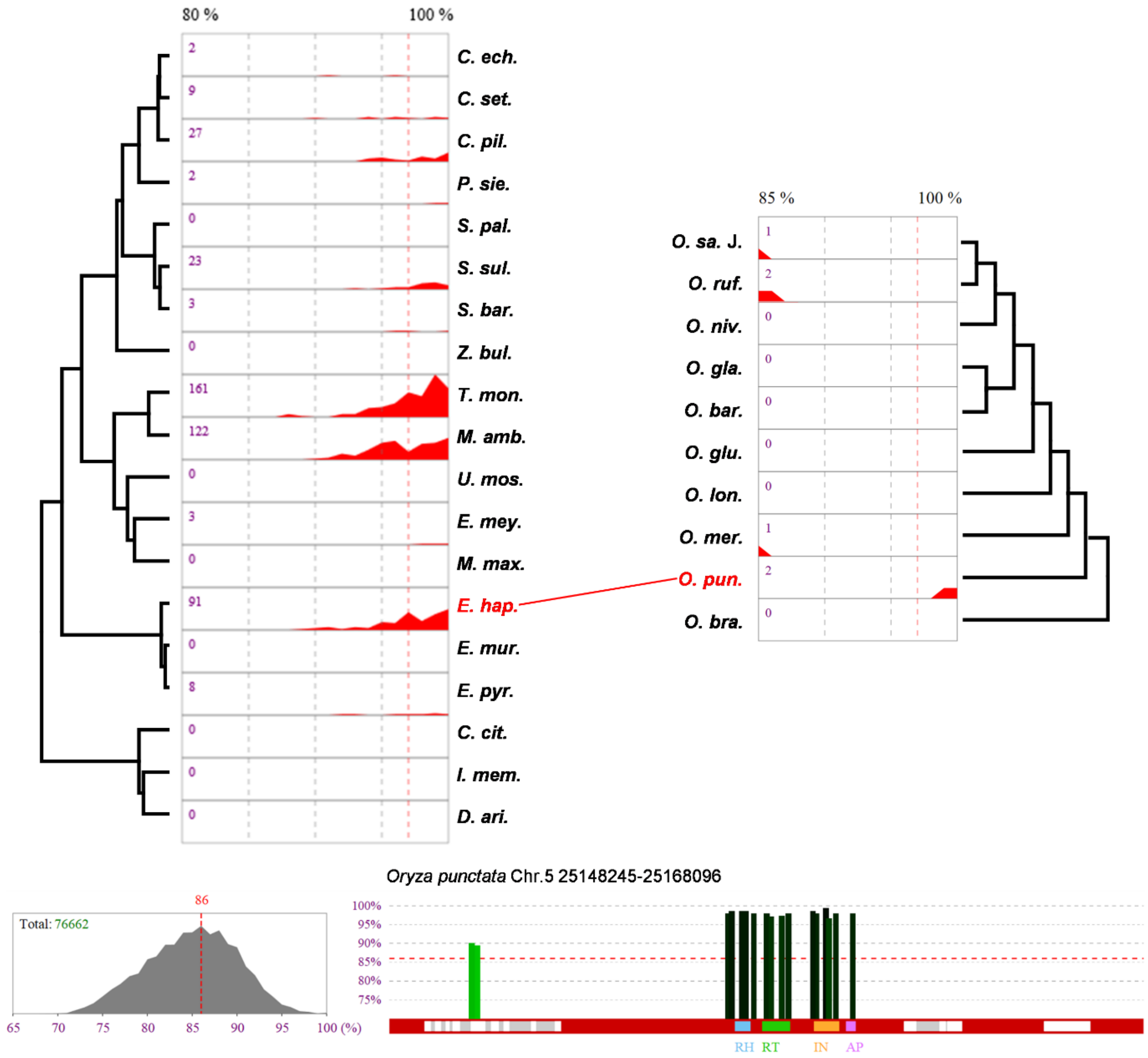

CL212 *Guhis* (*Gypsy*)

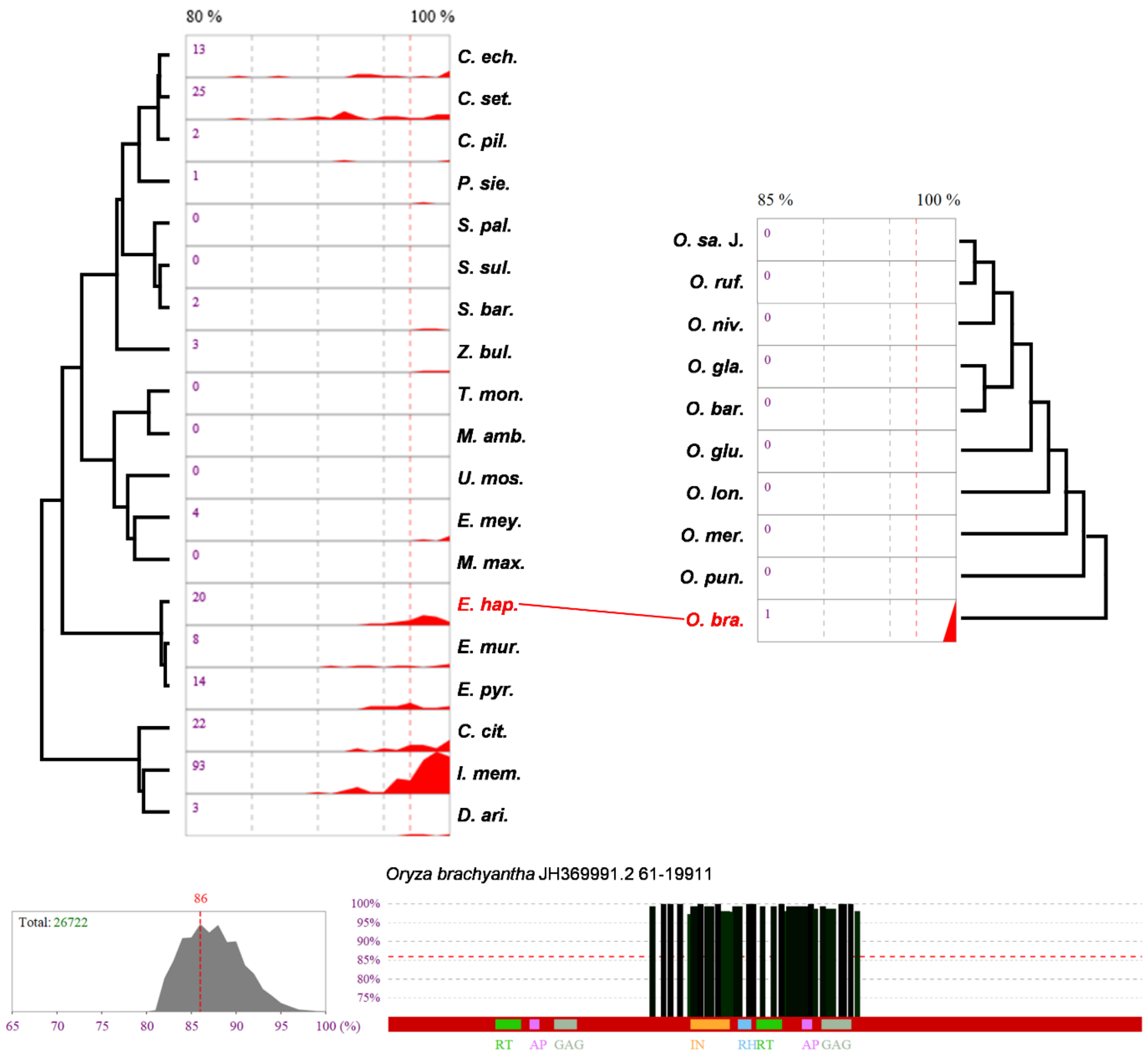

CL329 *Hera* (*Copia*)

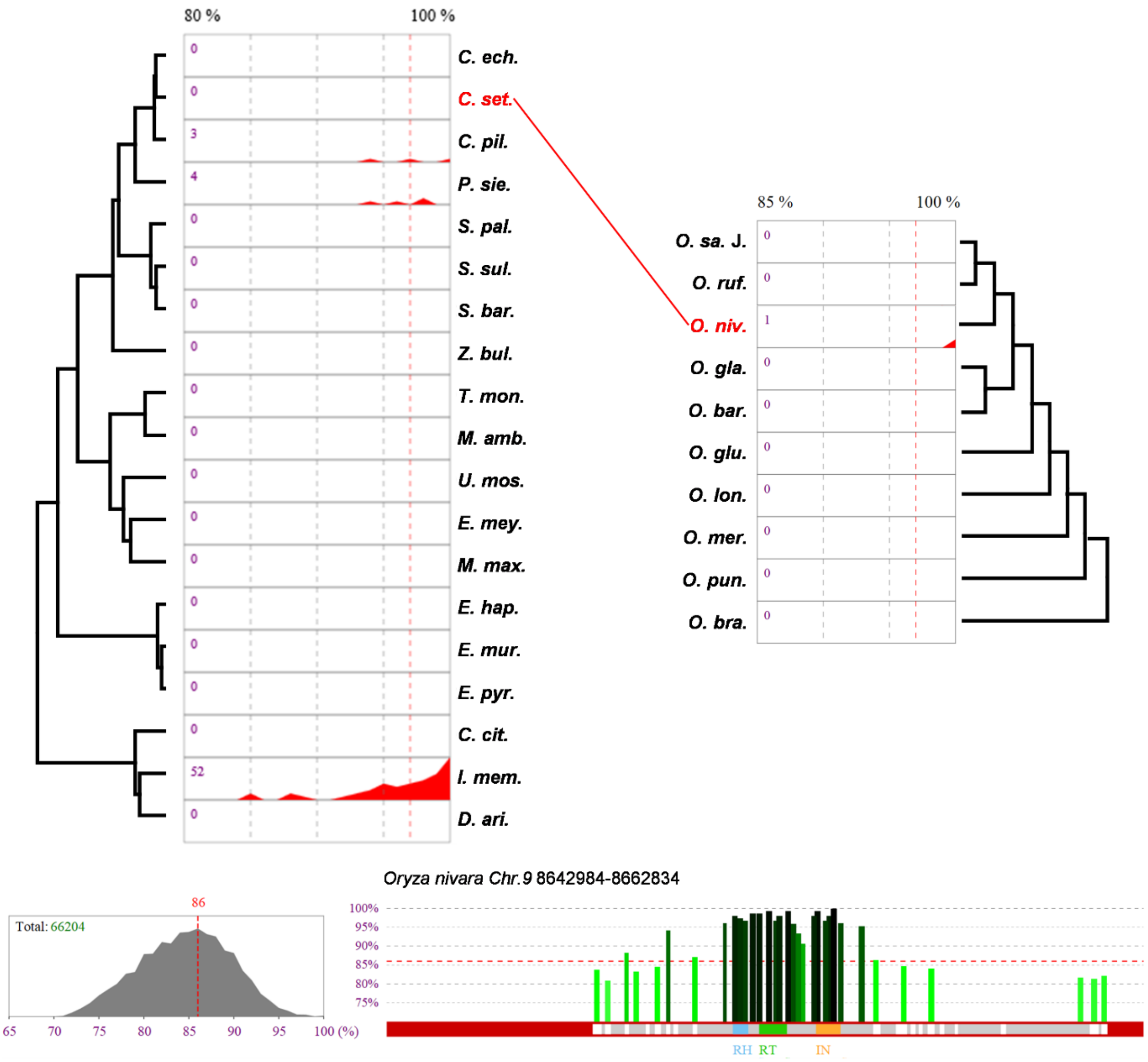

Supplementary FIG. S3

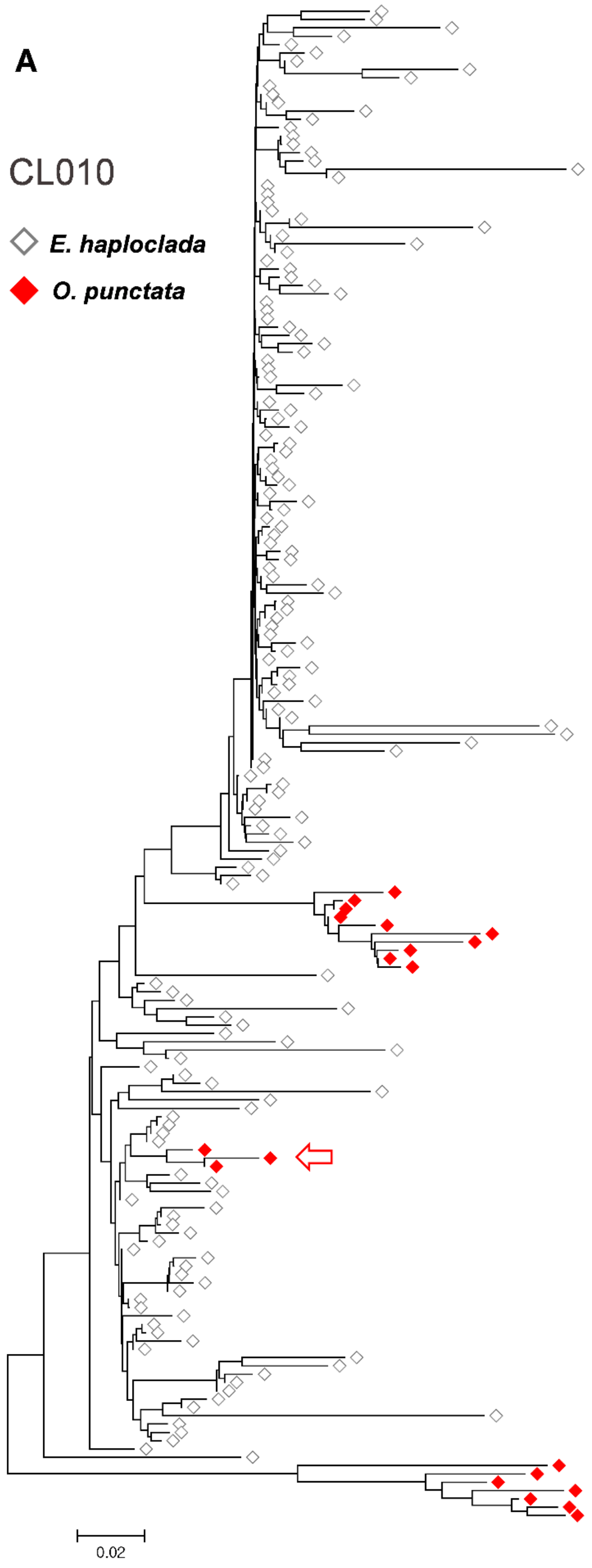

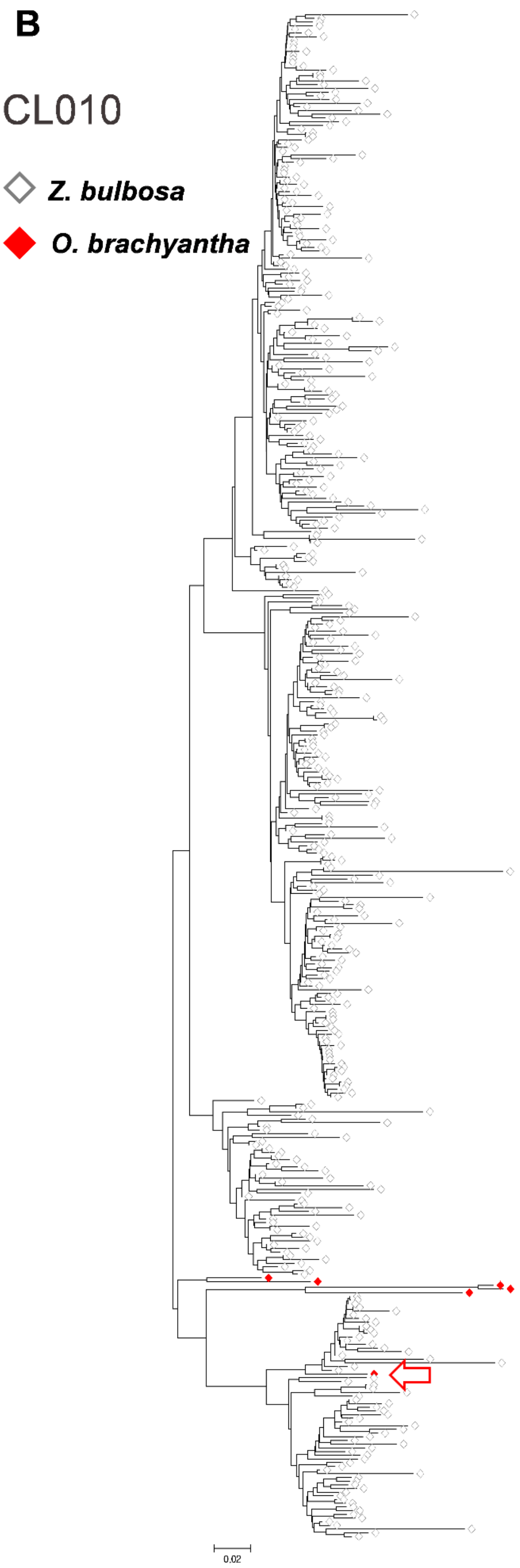

B
CL010
Z. bulbosa
O. brachyantha
0.02

C CL129

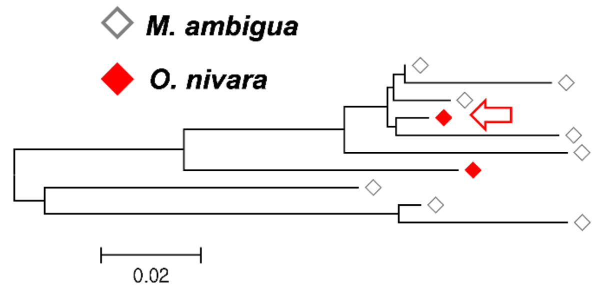

D CL129

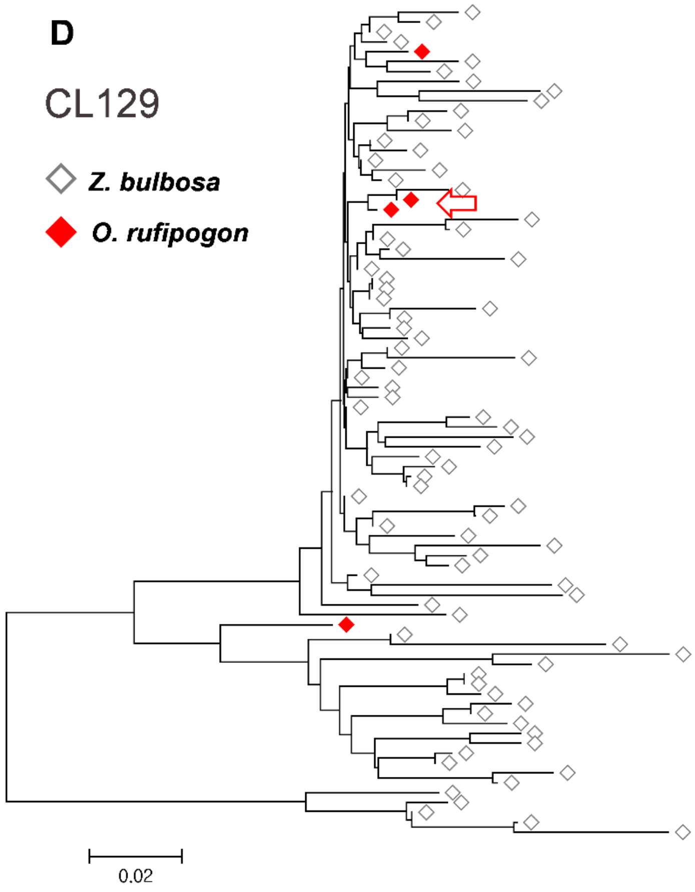

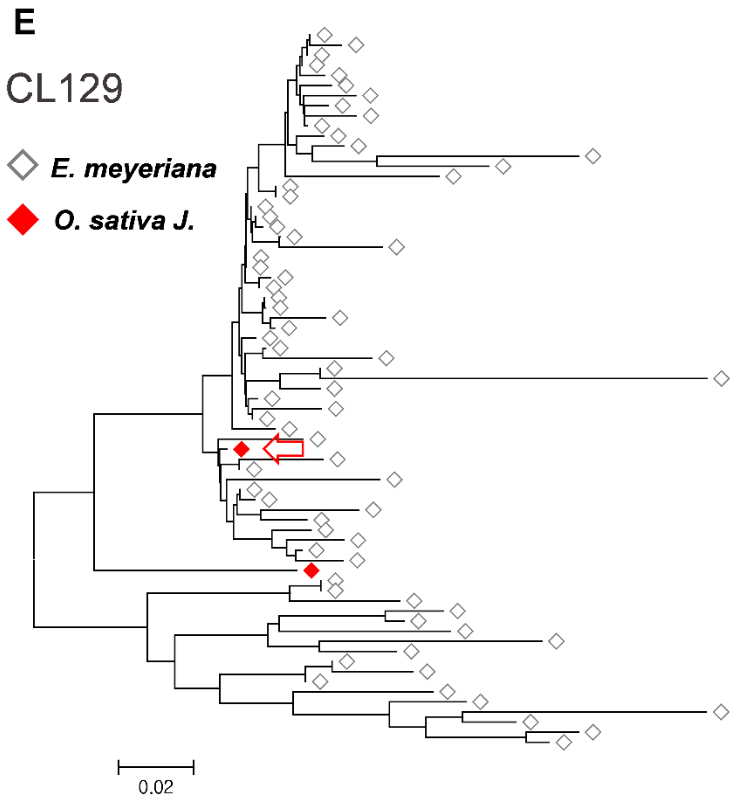
E
CL129
E. meyeriana
O. sativa J.
0.02

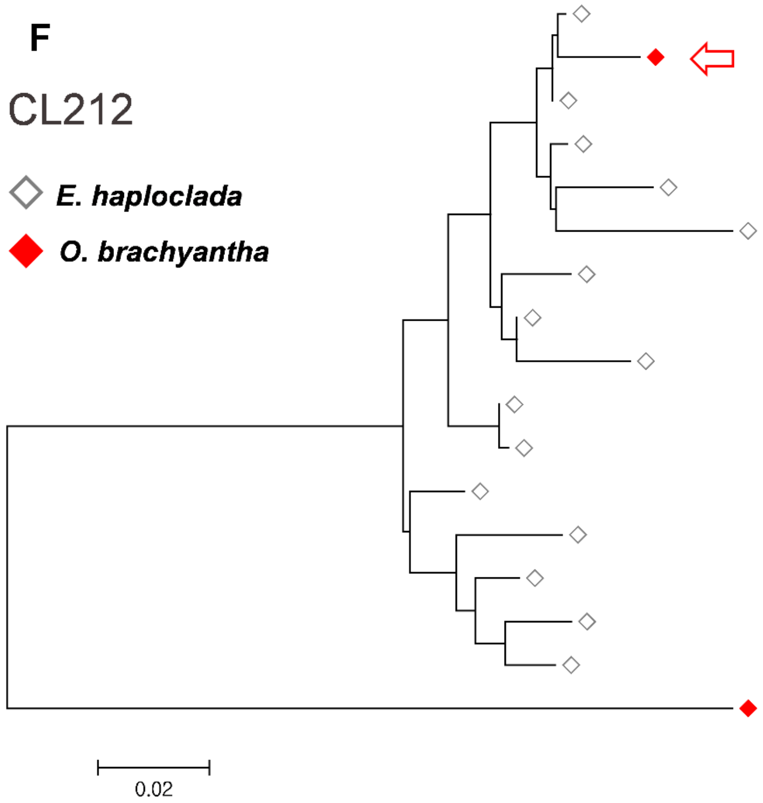
F
CL212
E. haploclada
O. brachyantha
0.02

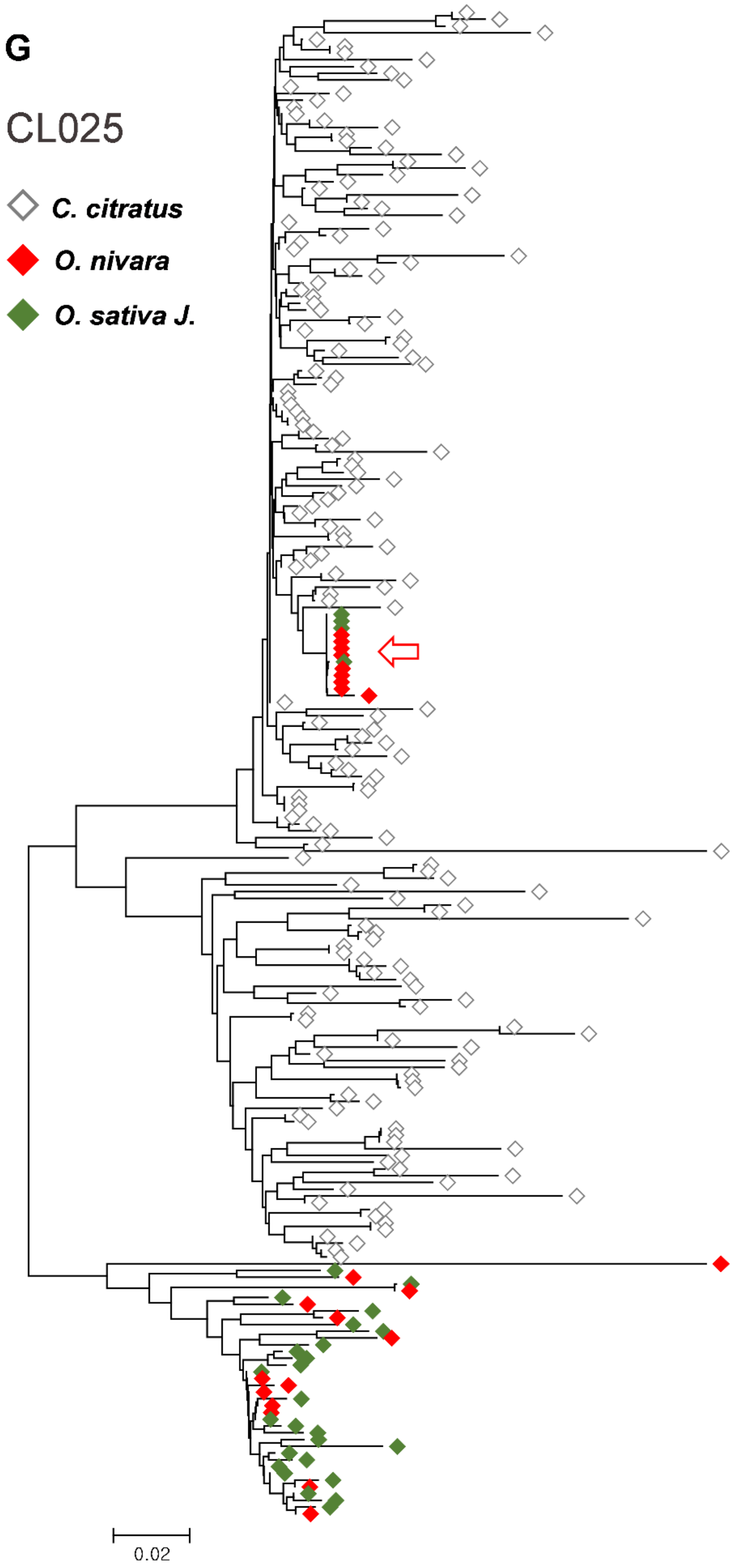

G
CL025
C. citratus
O. nivara
O. sativa J.
0.02

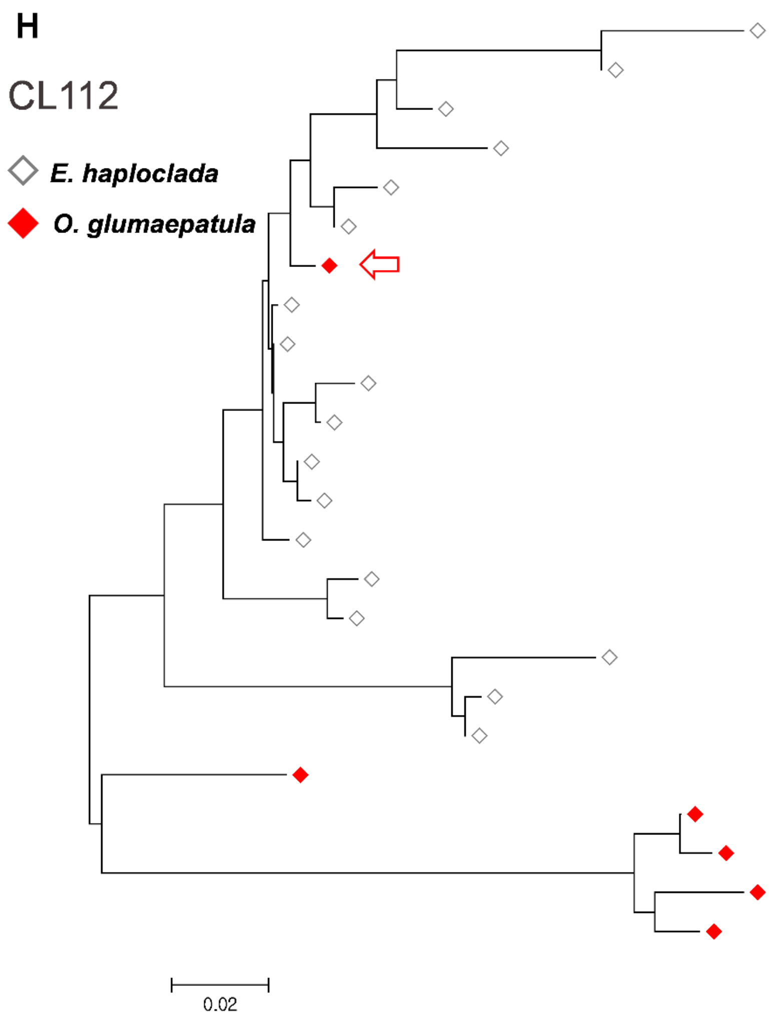
H
CL112
E. haplaclada
O. glumaepatula
0.02

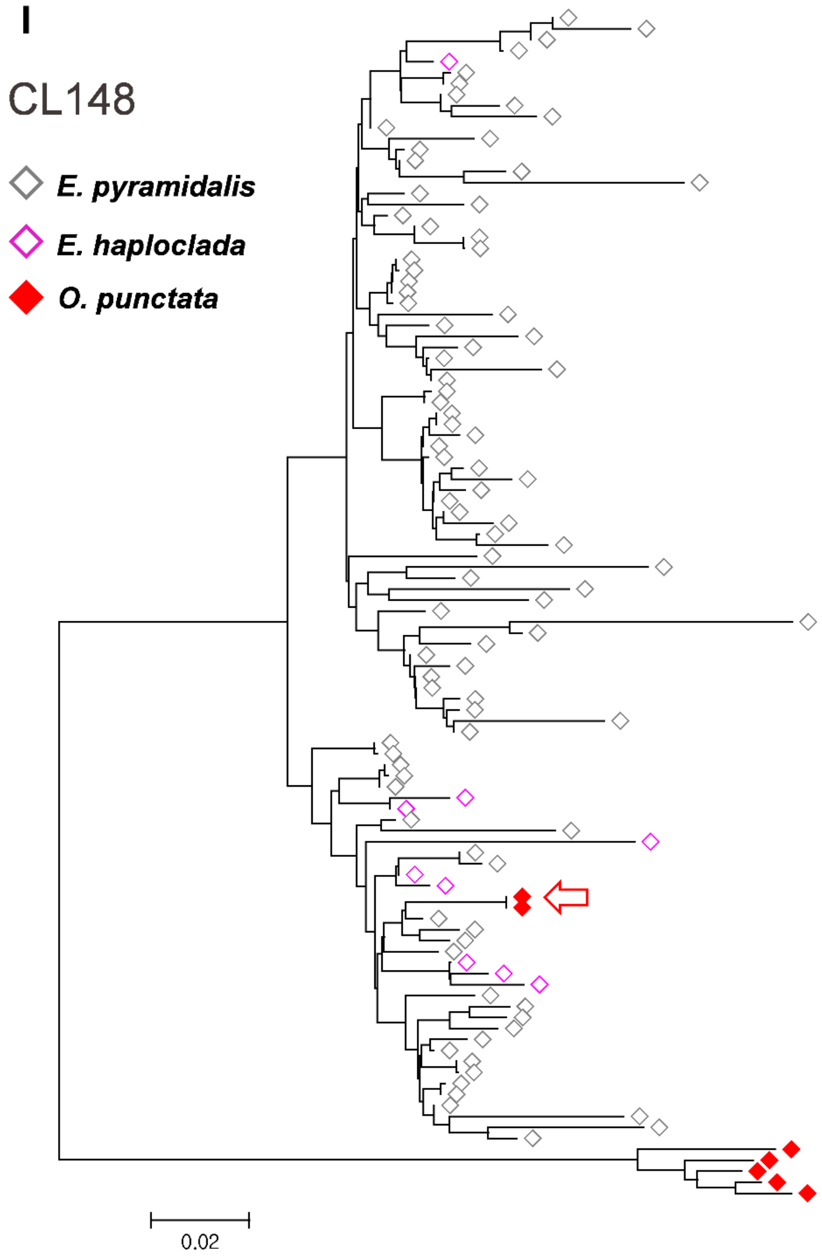
I
CL148
E. pyramidalis
E. haploclada
O. punctata
0.02

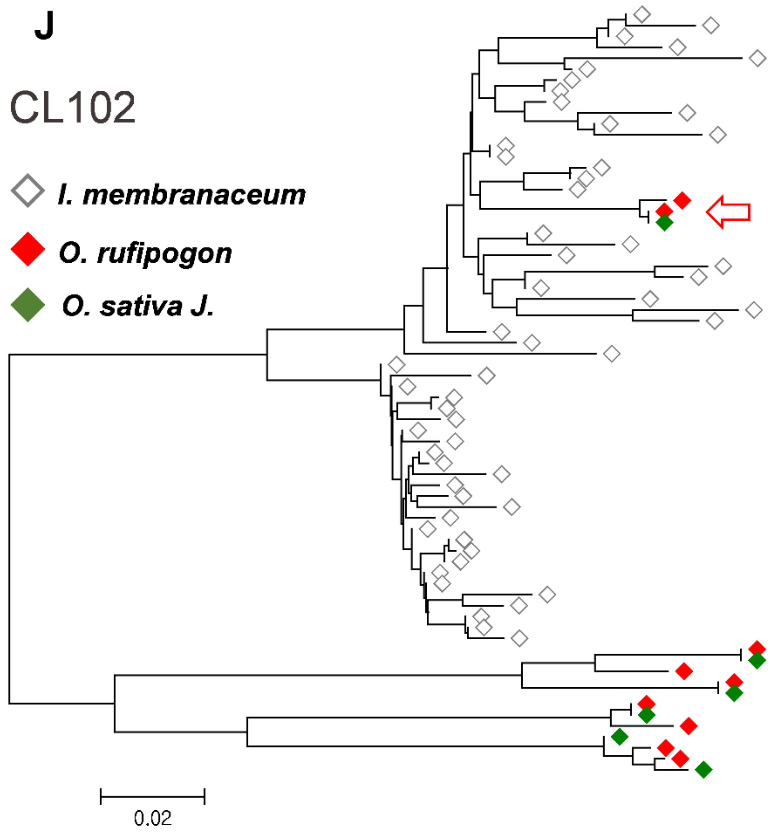
J
CL102
I. membranaceum
O. rufipogon
O. sativa J.
0.02

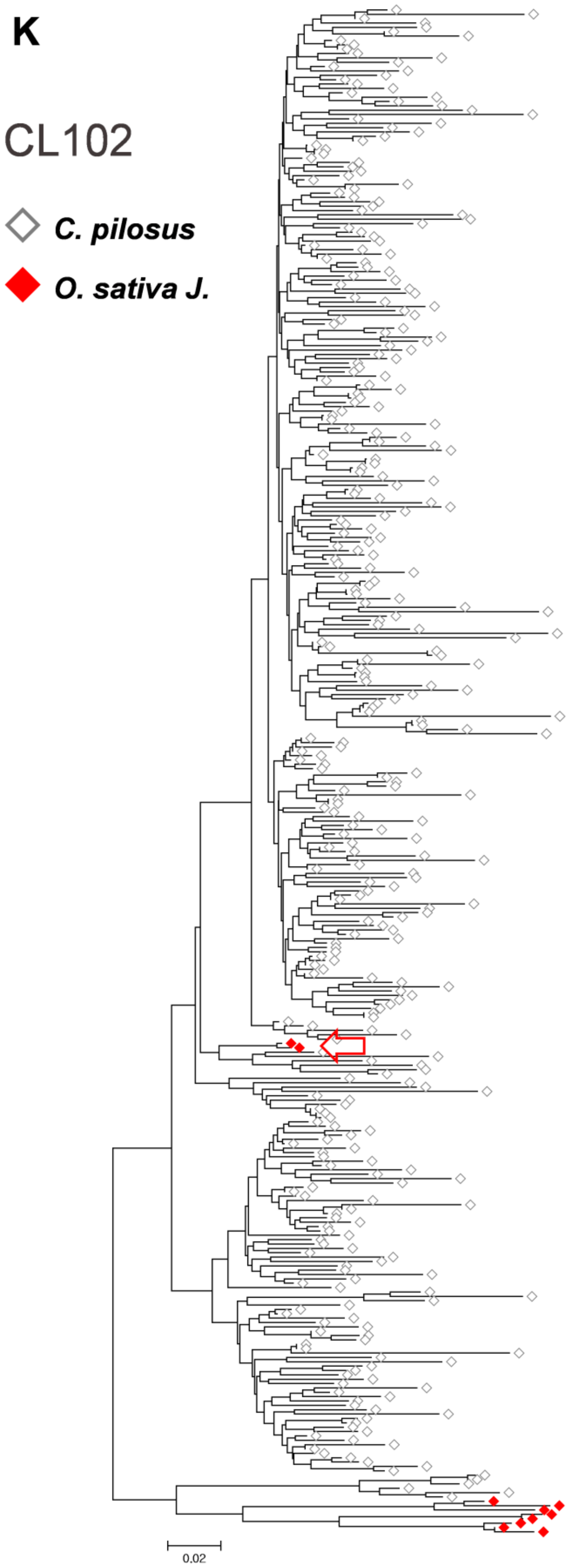
K
CL102
C. pilosus
O. sativa J.
0.02

Supplementary FIG. S4

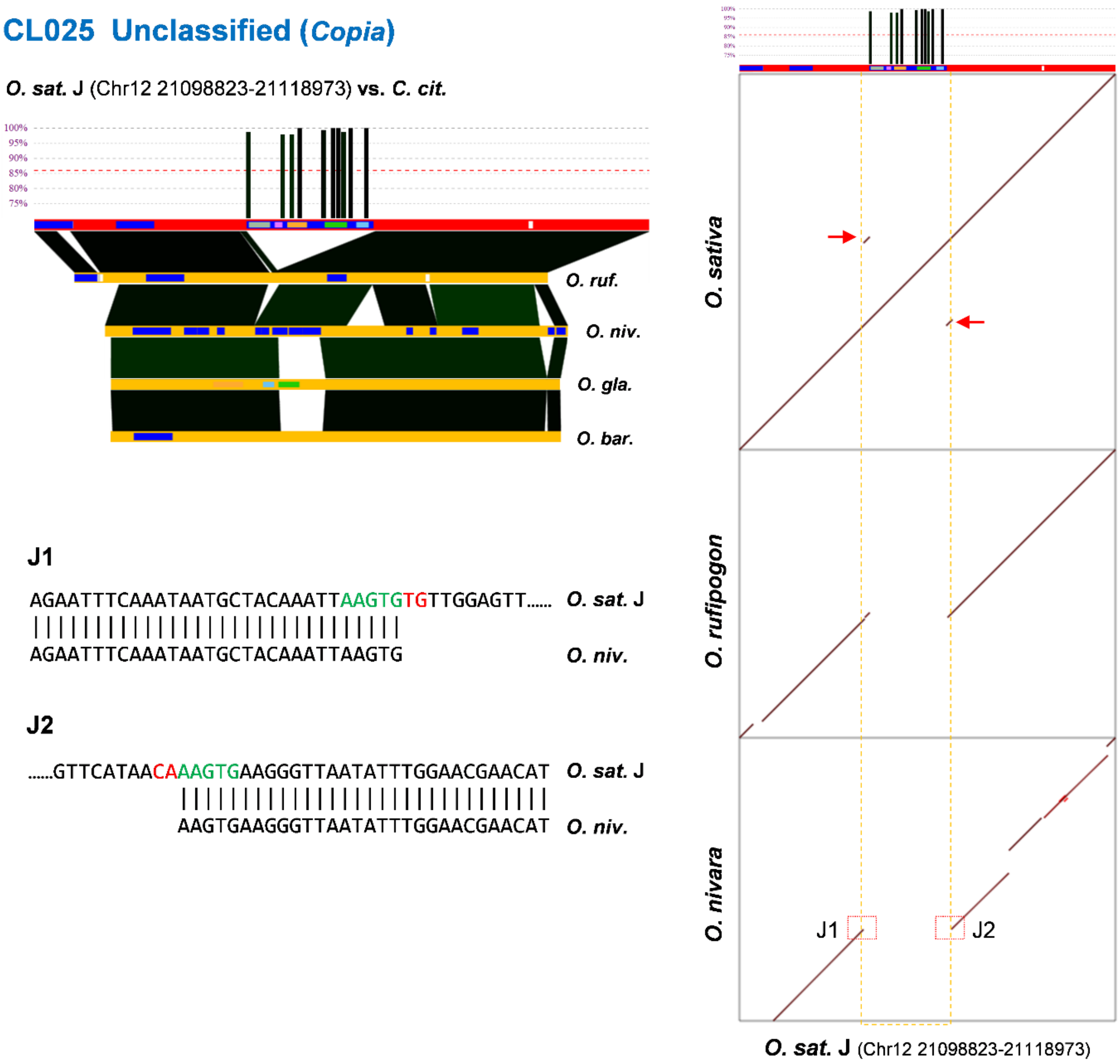

CL102 *Lusi* (*Copia*)

O. sat.* J** (Chr06 24815675-24835525) **vs. *I. mem.

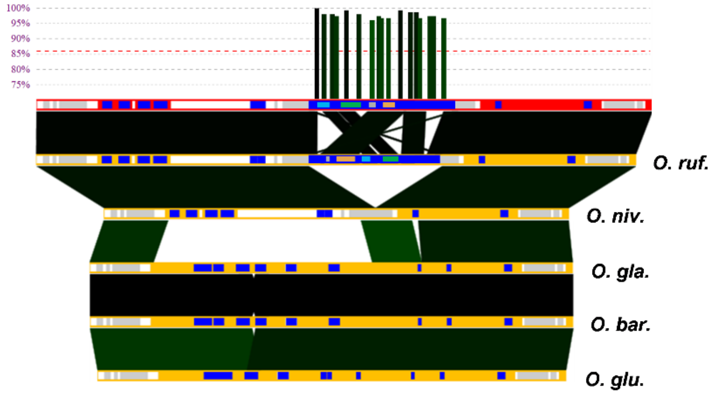

J1

```
CATGATGCTTGATTCAAATATGGGAGATTTTGTAGAGAAT......   O. sat. J
||||||||||||||||||||||||||||||||
CATGATGCTTGATTCAAATATGGGAGATTT                   O. niv.
```

J2

```
......ATTGCCTAACAGATTTAACTATCAAAGAGGATGCACCTCT   O. sat. J
                  ||||||||||||||||||||||||||||||
                  AGATTTAACTATCAAAGAGGATGCACCTCT   O. niv.
```

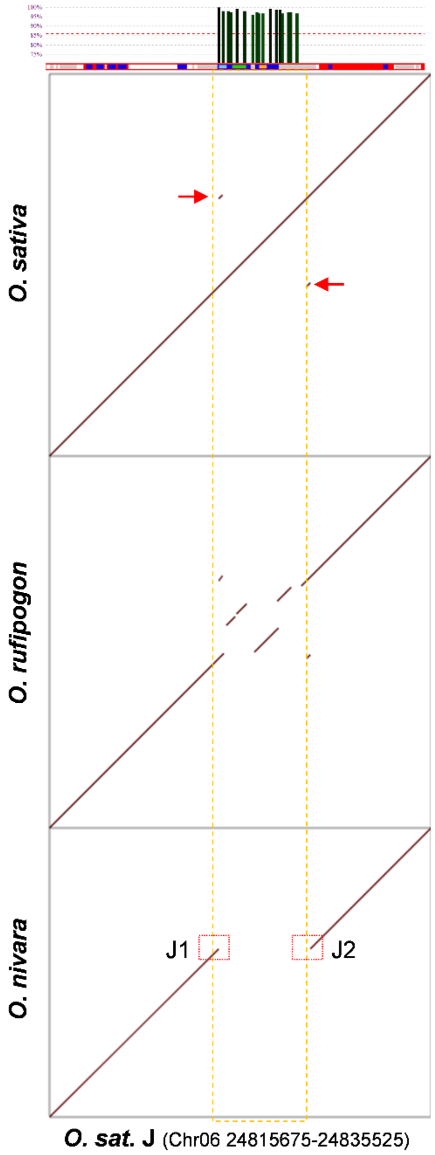

CL102 *Lusi* (*Copia*)

O. sat.* J (Chr02 1761599-1781749) vs. *C. pil.

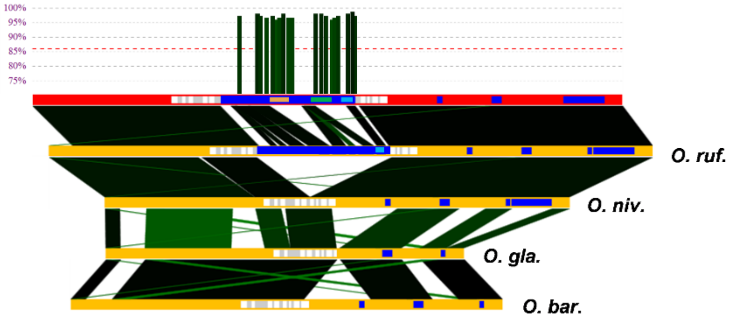

J1

```
ATACGTCTCAAAAGCGTCAGTAGGCAATGGTGTTAGGCAA......   O. sat. J
||||||||||||||||||||||||||||||
ATACGTCTCAAAAGCGTCAGTAGGCAATGG                   O. niv.
```

J2

```
......ATTCTCTACAAATGGGCCCAAGTCTCGGATCTTCTTCATA   O. sat. J
              ||||||||||||||||||||||||||||||
              AATGGGCCCAAGTCTCGGATCTTCTTCATA   O. niv.
```

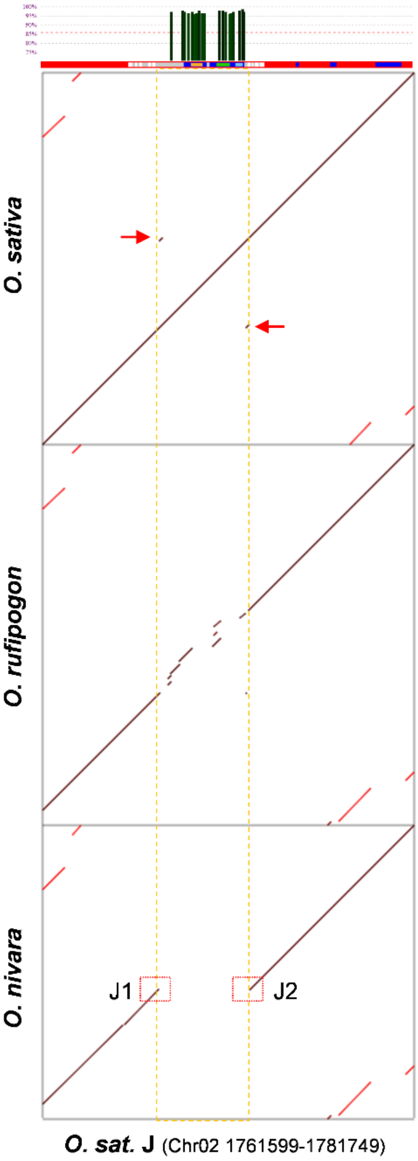

CL129 *Wihov* (*Gypsy*)

O. ruf. (Chr10 7047133-7067282) **vs.** ***Z. bul.***

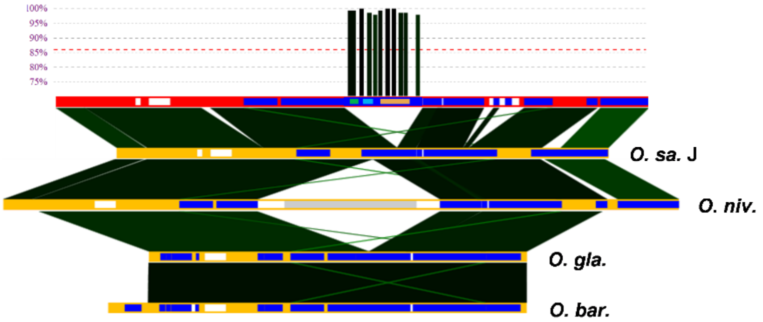

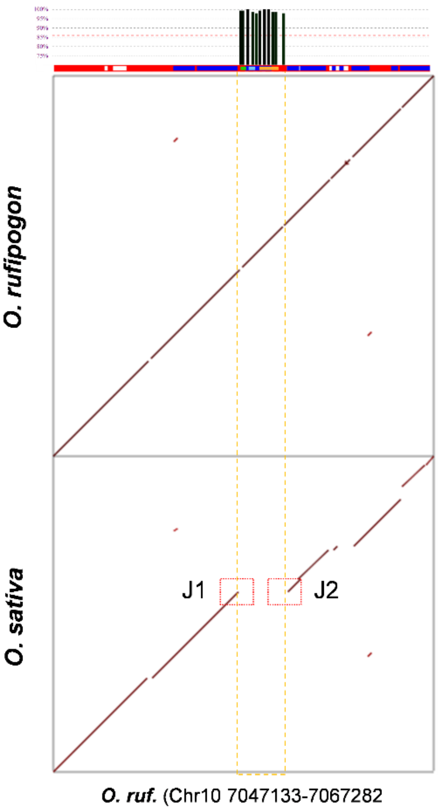

J1

```
TGCACAACTTCTCGCTCCCGCAAACACCTATAATGAAGAC......   O. sat. J
||||||||||||||||||||||||||||||
TGCACAACTTCTCGCTCCCGCAAACACCTA                   O. ruf.
```

J2

```
......GTGCATATCGACCTACATGCATACAAGCAAGAGGAAATGC   O. sat. J
                ||||||||||||||||||||||||||||||
                ACCTACATGCATACAAGCAAGAGGAAATGC   O. ruf.
```

Supplementary FIG. S5

CL102 *Lusi* (*Copia*) *O. sat.* J - *C. pil.*

Junction 1

```
O. sat.  38   ATTTATATGGAAACAAAACTAGGTAGCCATGAAAAATGCAATACGTCTCAAAAGCGTCAG   97
              ||||||||||||||||||||||||||||||||||||||||||||||||||||||||||||
Raw read 150  ATTTATATGGAAACAAAACTAGGTAGCCATGAAAAATGCAATACGTCTCAAAAGCGTCAG   91

         98   TAGGCAATGGTGTTAGGCAATAGAATATGCCGTCTGTGGATATCCCAGCTGCTATAGATA   157
              ||||||||||||||||||||||||||||||||||||||||||||||||||||||||||||
         90   TAGGCAATGGTGTTAGGCAATAGAATATGCCGTCTGTGGATATCCCAGCTGCTATAGATA   31

         158  GCAGATGTAAATCAGGGATTAGCTACTATA   187
              ||||||||||||||||||||||||||||||
         30   GCAGATGTAAATCAGGGATTAGCTACTATA   1
```

Junction 2

```
O. sat.  35   CGGCCCTATATAACAGAAGGCGCCCCCTCCCATTGAGGCGTGGCCAATTGCCCCCTAATC   94
              ||||||||||||||||||||||||||||||||||||||||||||||||||||||||||||
Raw read 1    CGGCCCTATATAACAGAAGGCGCCCCCTCCCATTGAGGCGTGGCCAATTGCCCCCTAATC   60

         95   CAATTCTCTACAAATGGGCCCAAGTCTCGGATCTTCTTCATACACTCTTCTTGCTTAGCT   154
              ||||||||||||||||||||||||||||||||||||||||||||||||||||||||||||
         61   CAATTCTCTACAAATGGGCCCAAGTCTCGGATCTTCTTCATACACTCTTCTTGCTTAGCT   120

         155  AAATGCATGCTCCTGCTGCTCATGAGCTGT   184
              ||||||||||||||||||||||||||||||
         121  AAATGCATGCTCCTGCTGCTCATGAGCTGT   150
```

Supplementary FIG. S6

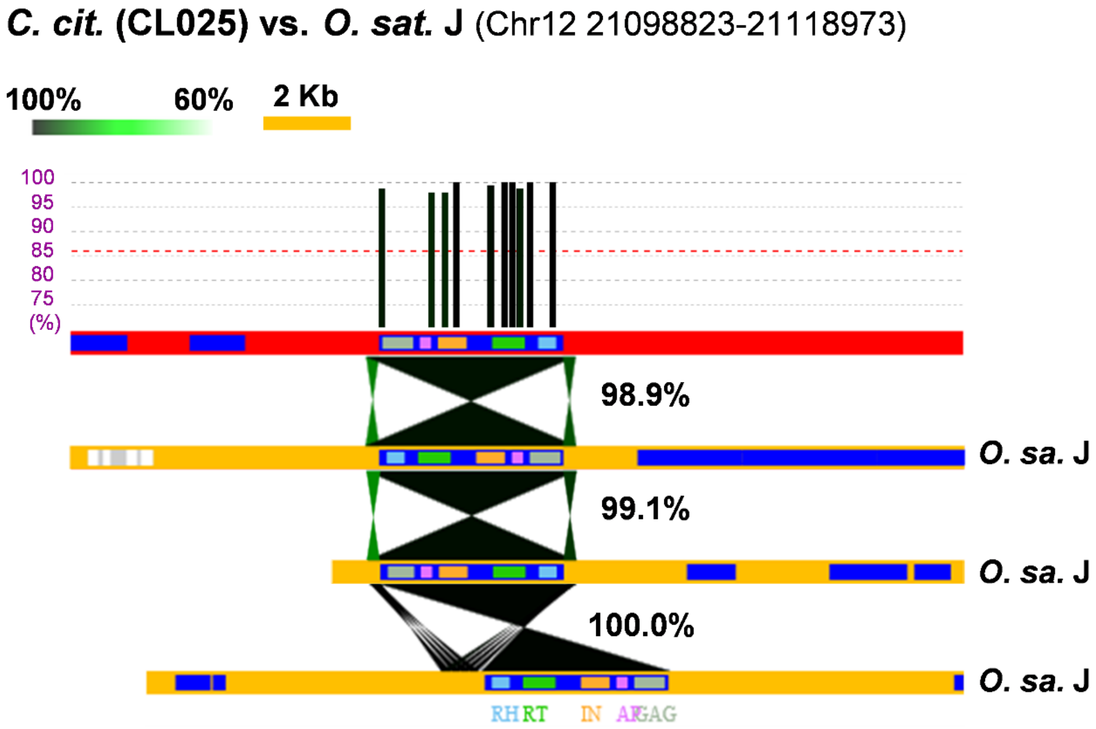

Supplementary FIG. S7

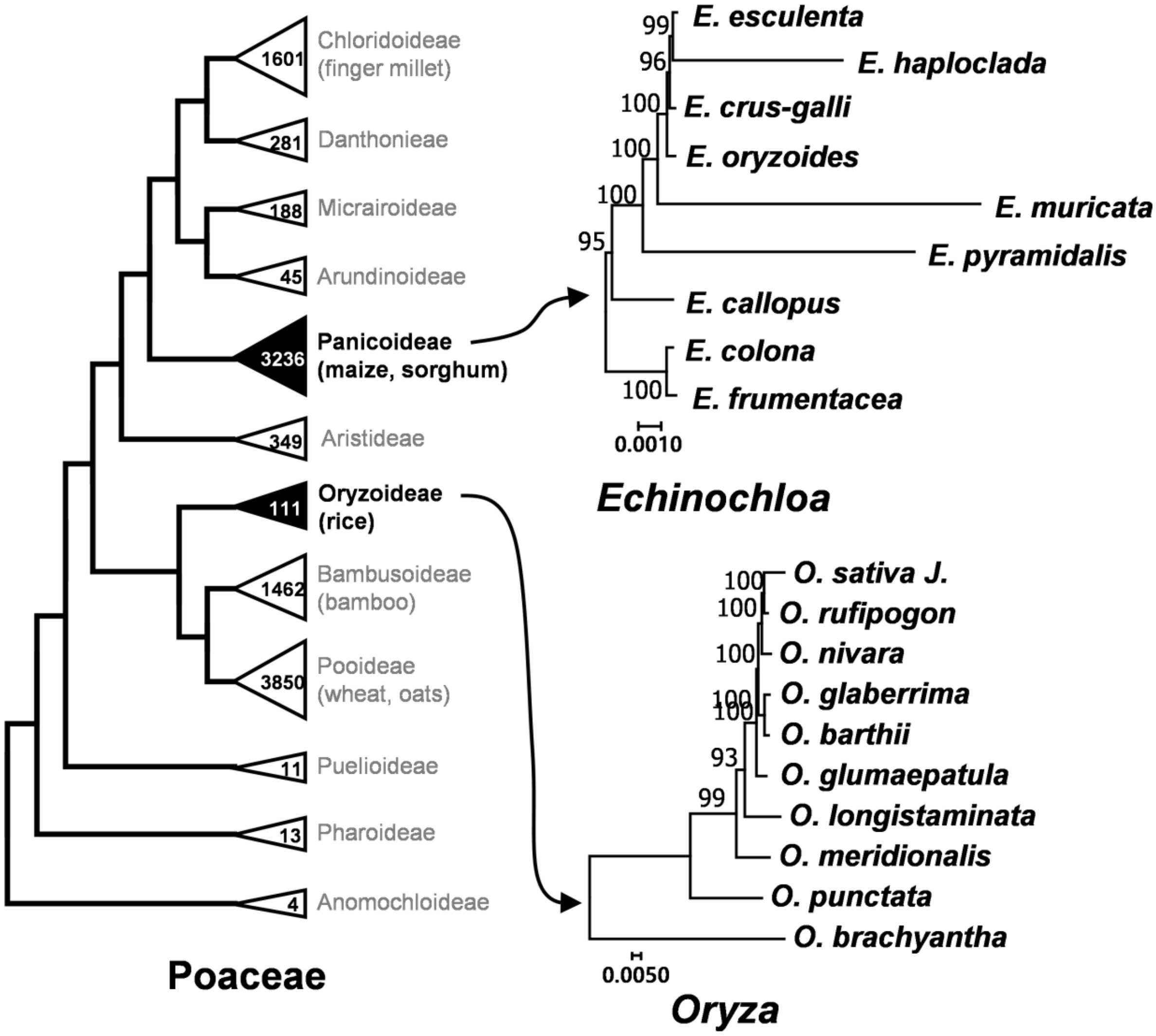

Supplementary FIG. S8

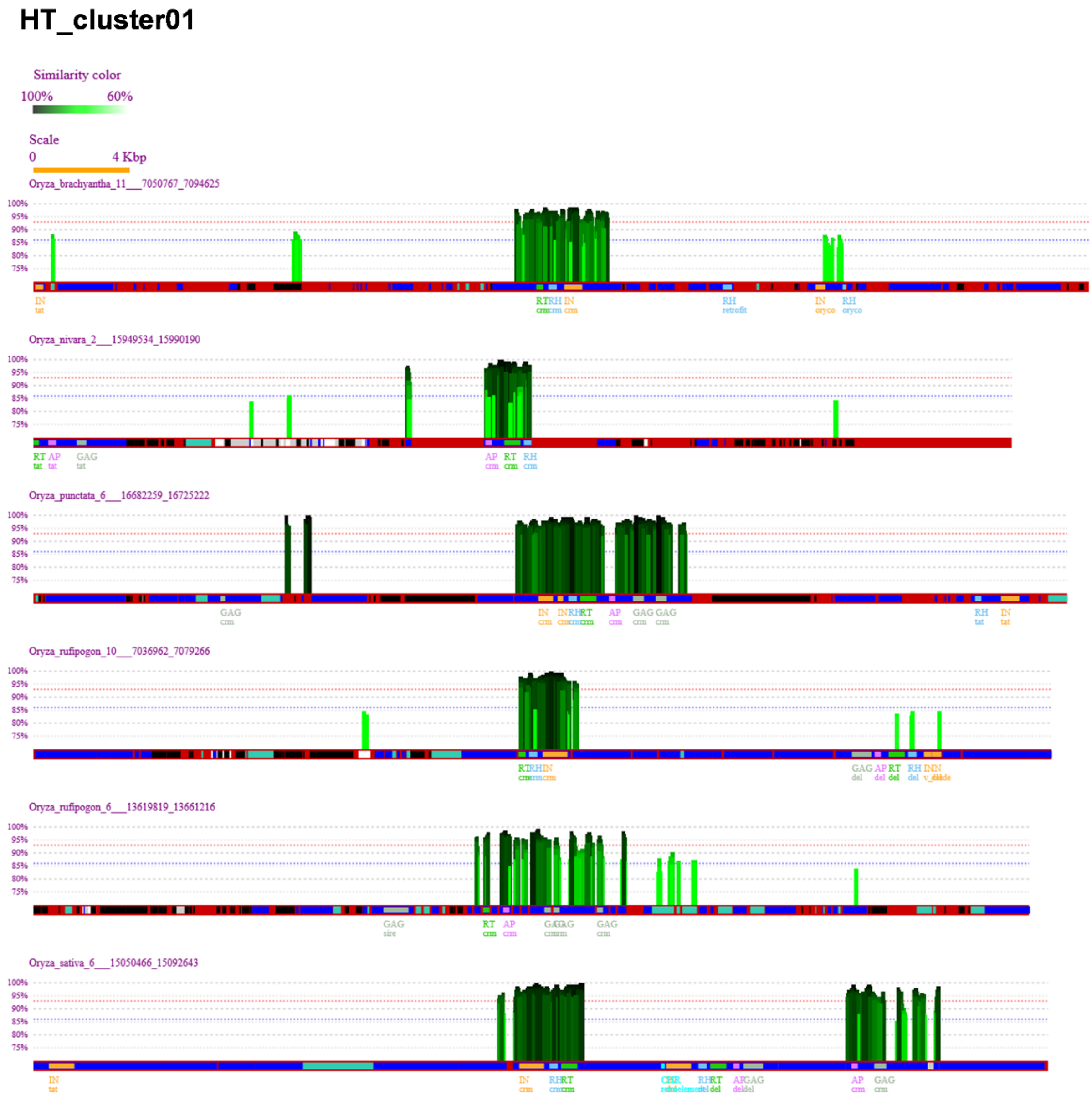

HT_cluster02

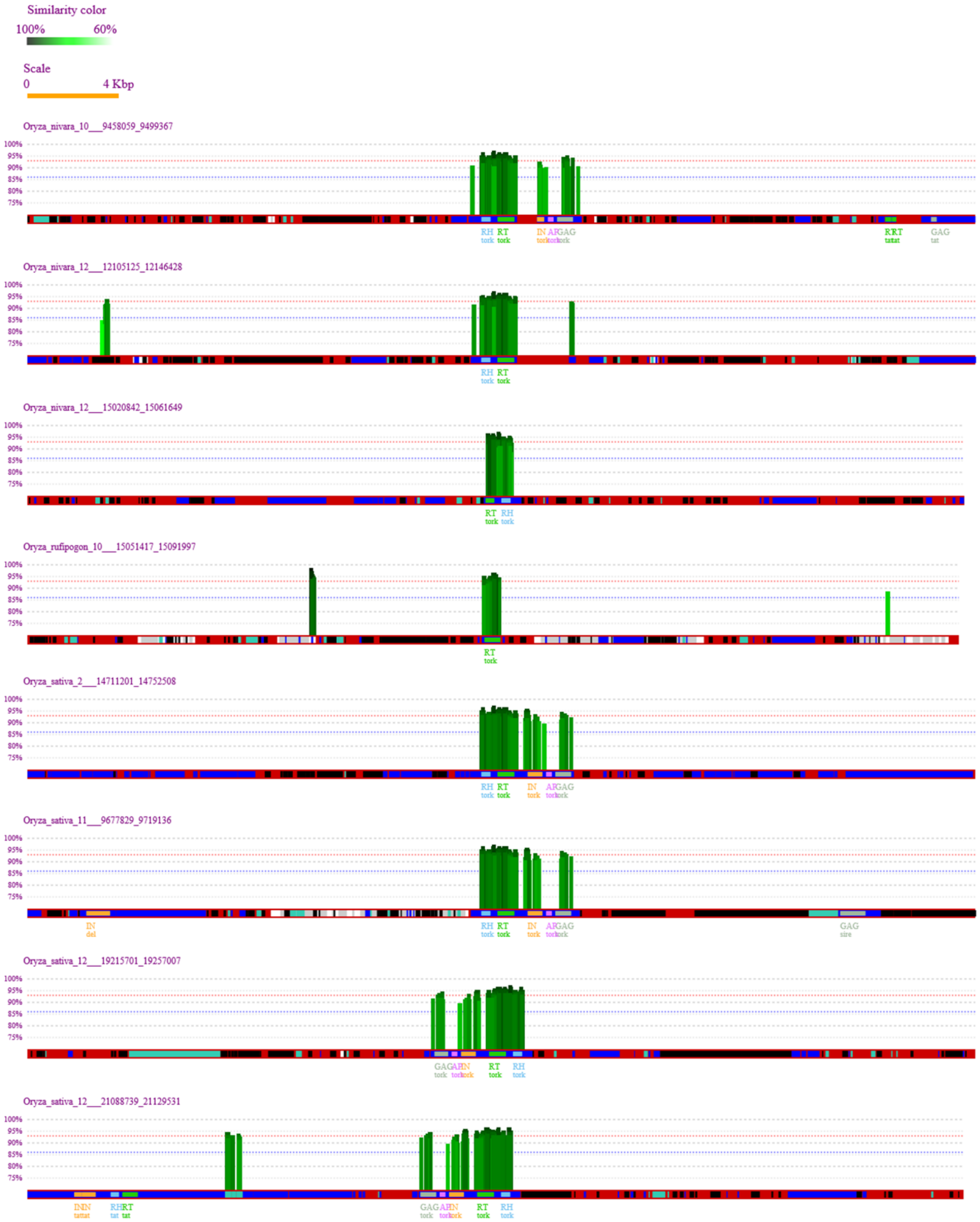

HT_cluster02

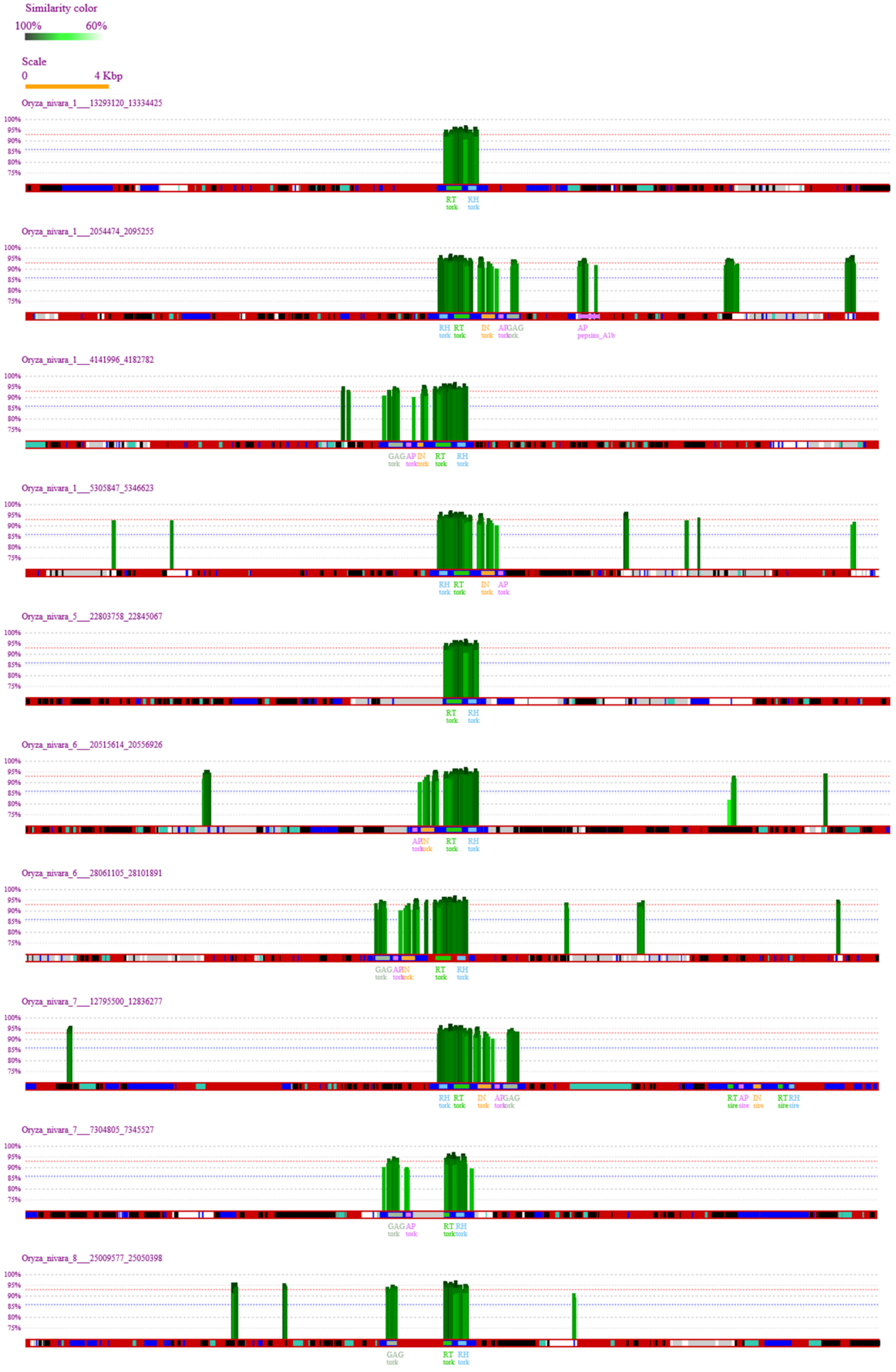

HT_cluster03

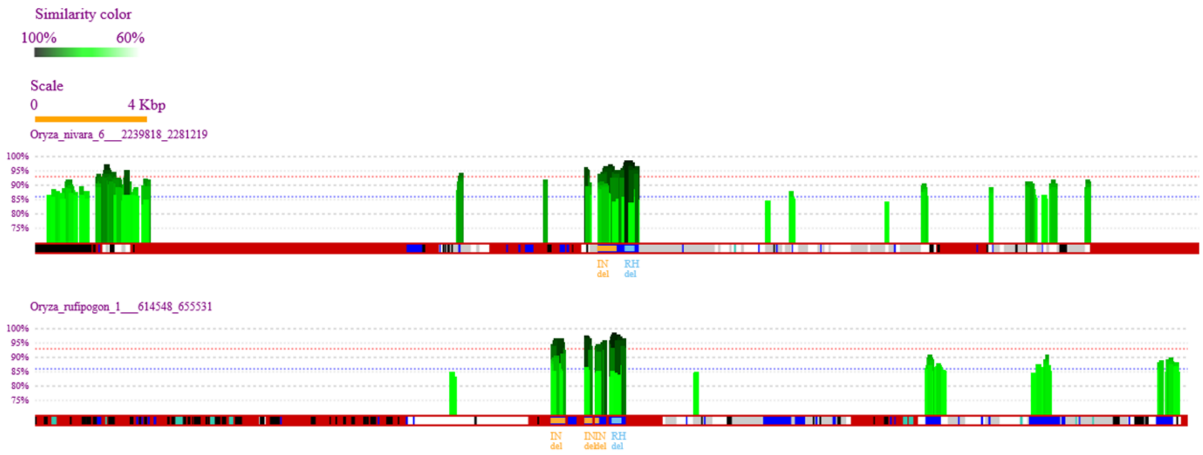

HT_cluster04

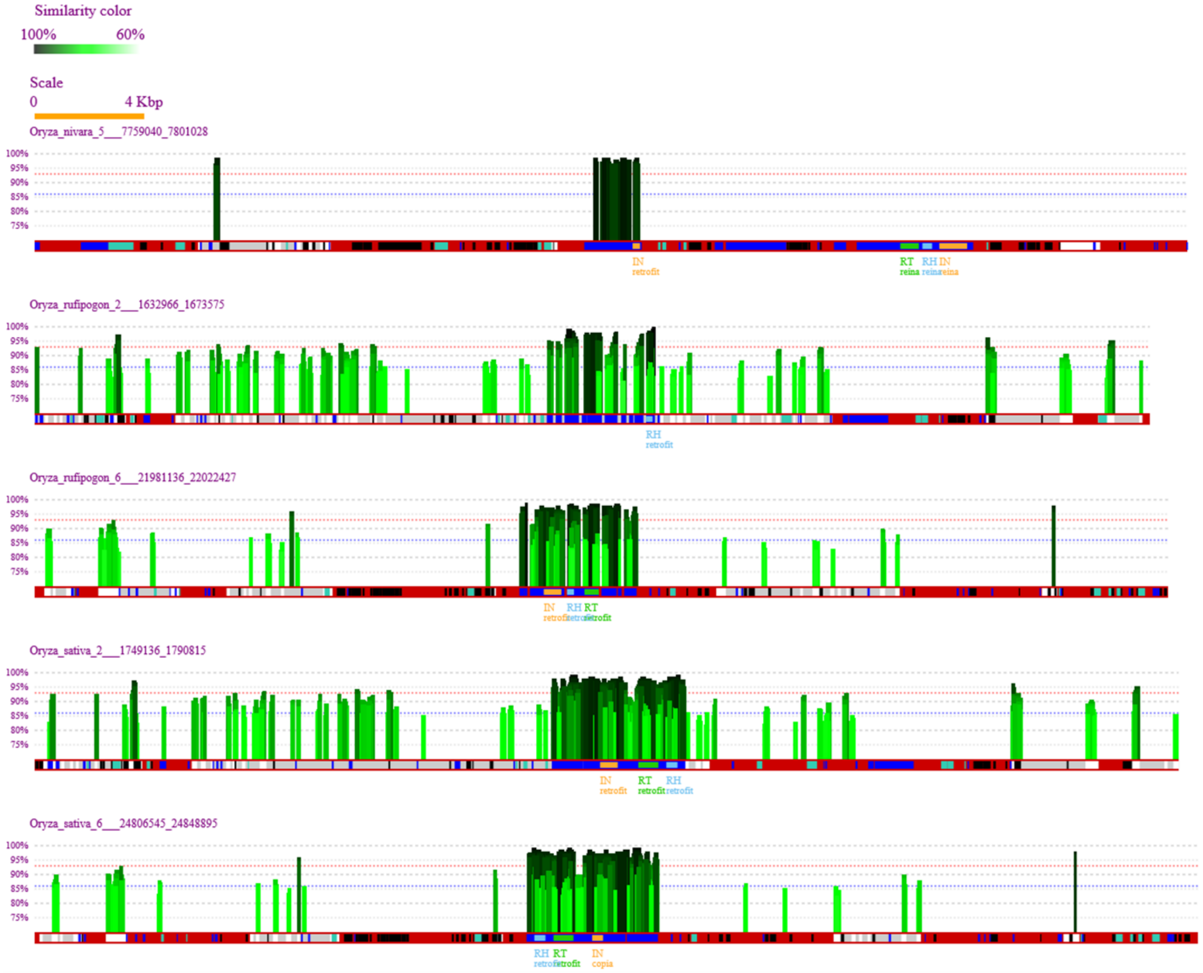

HT_cluster05

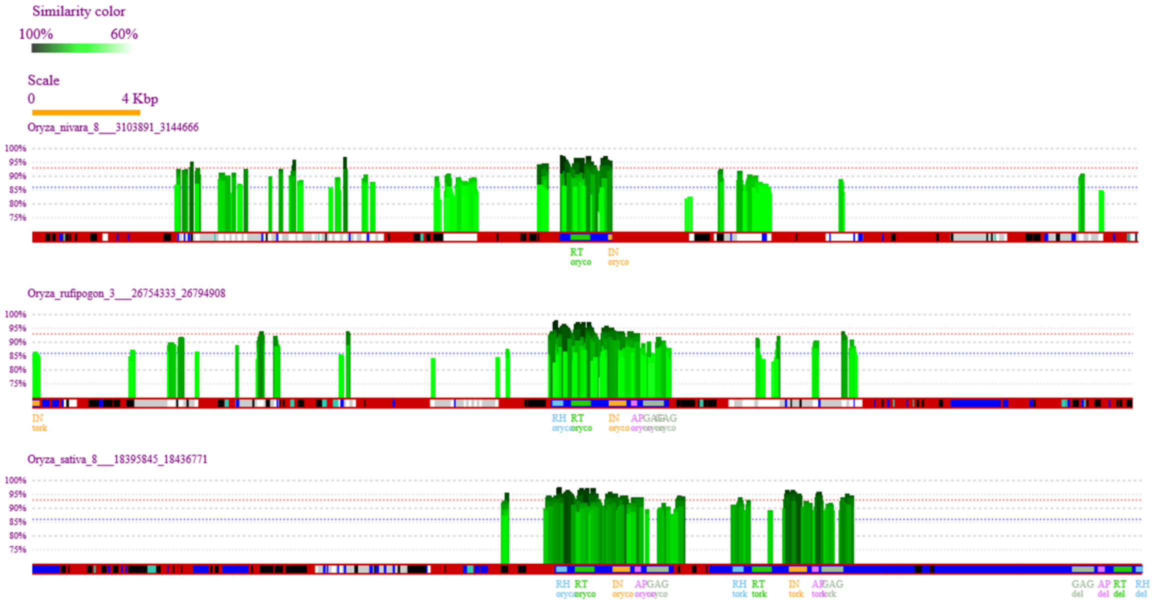

HT_cluster06

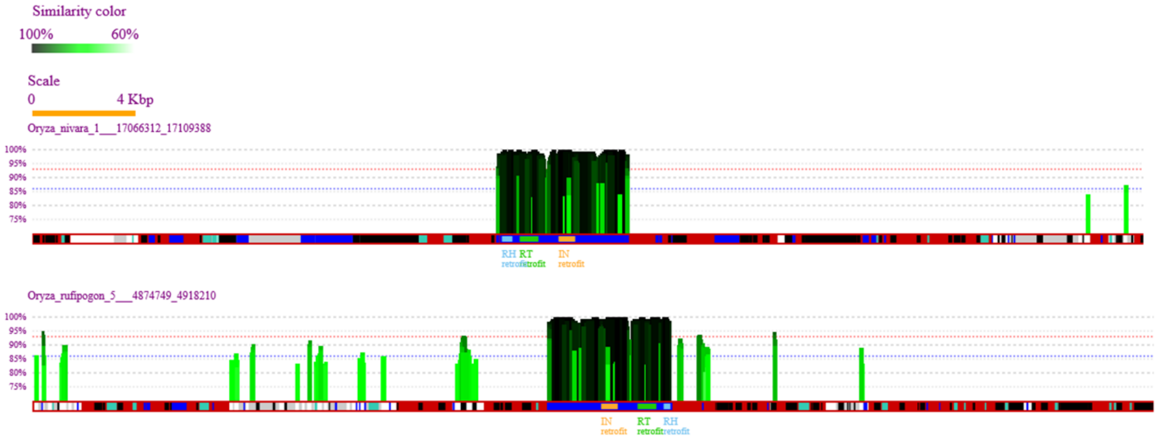

HT_cluster07

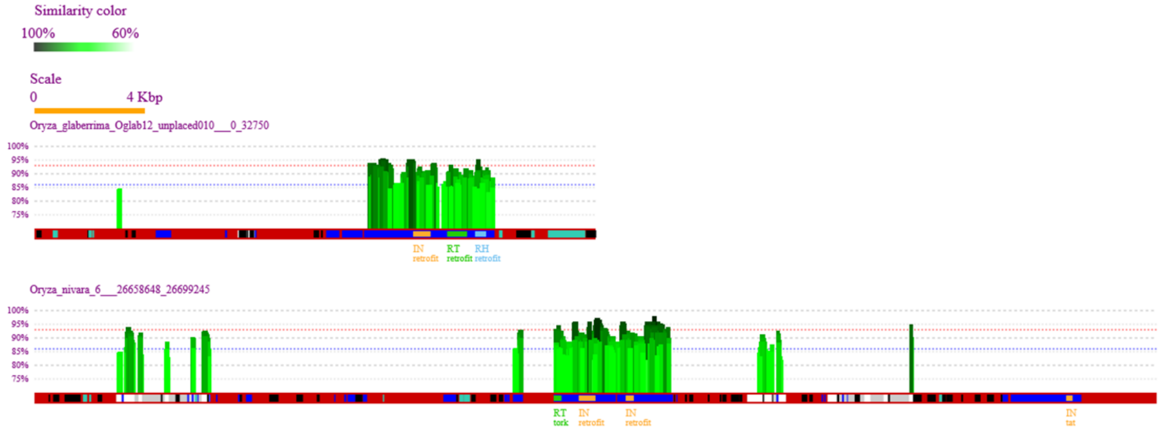

HT_cluster08

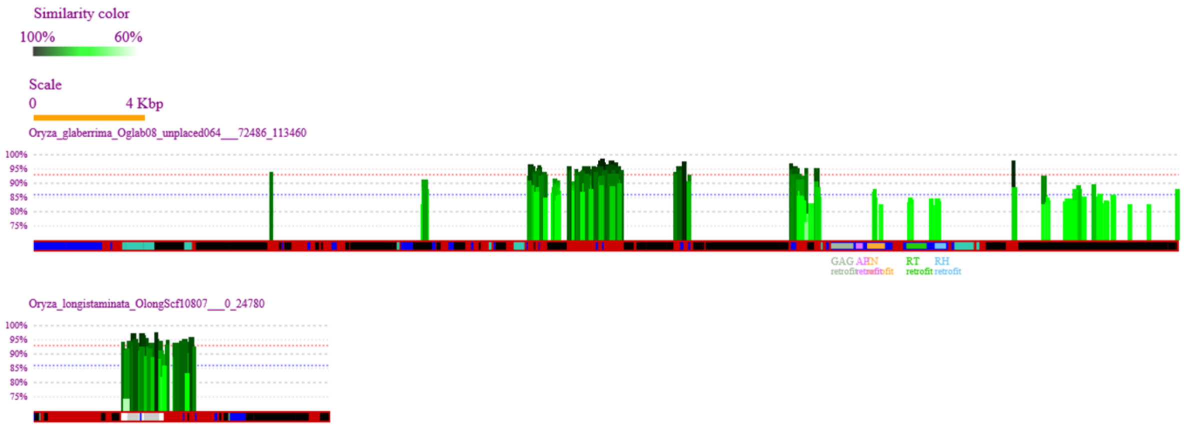

HT_cluster09

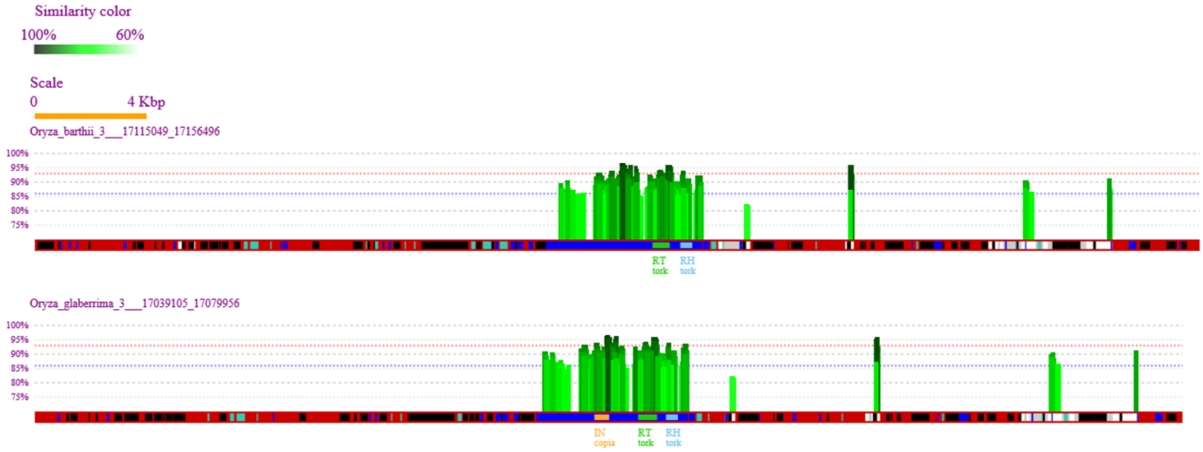

HT_cluster10

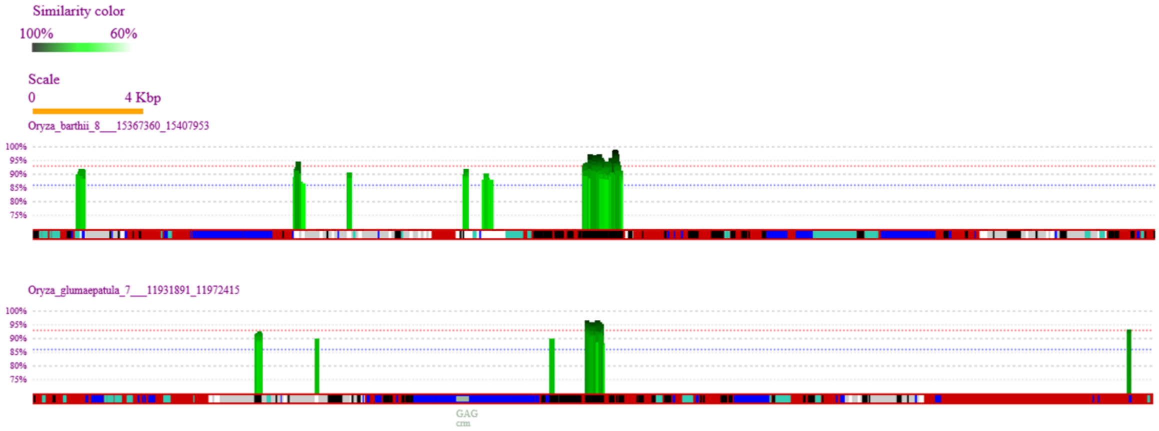

HT_cluster11

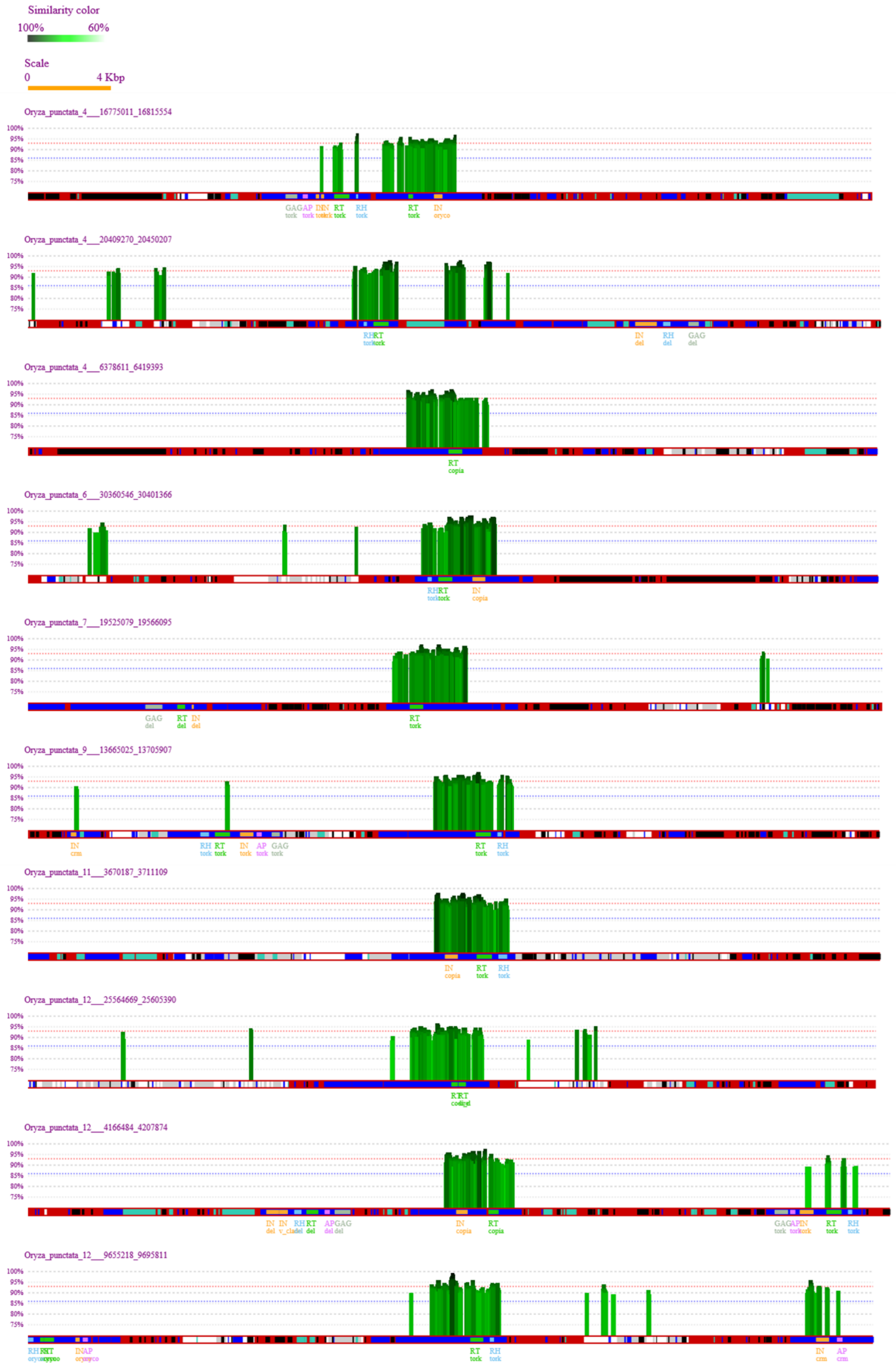

HT_cluster11

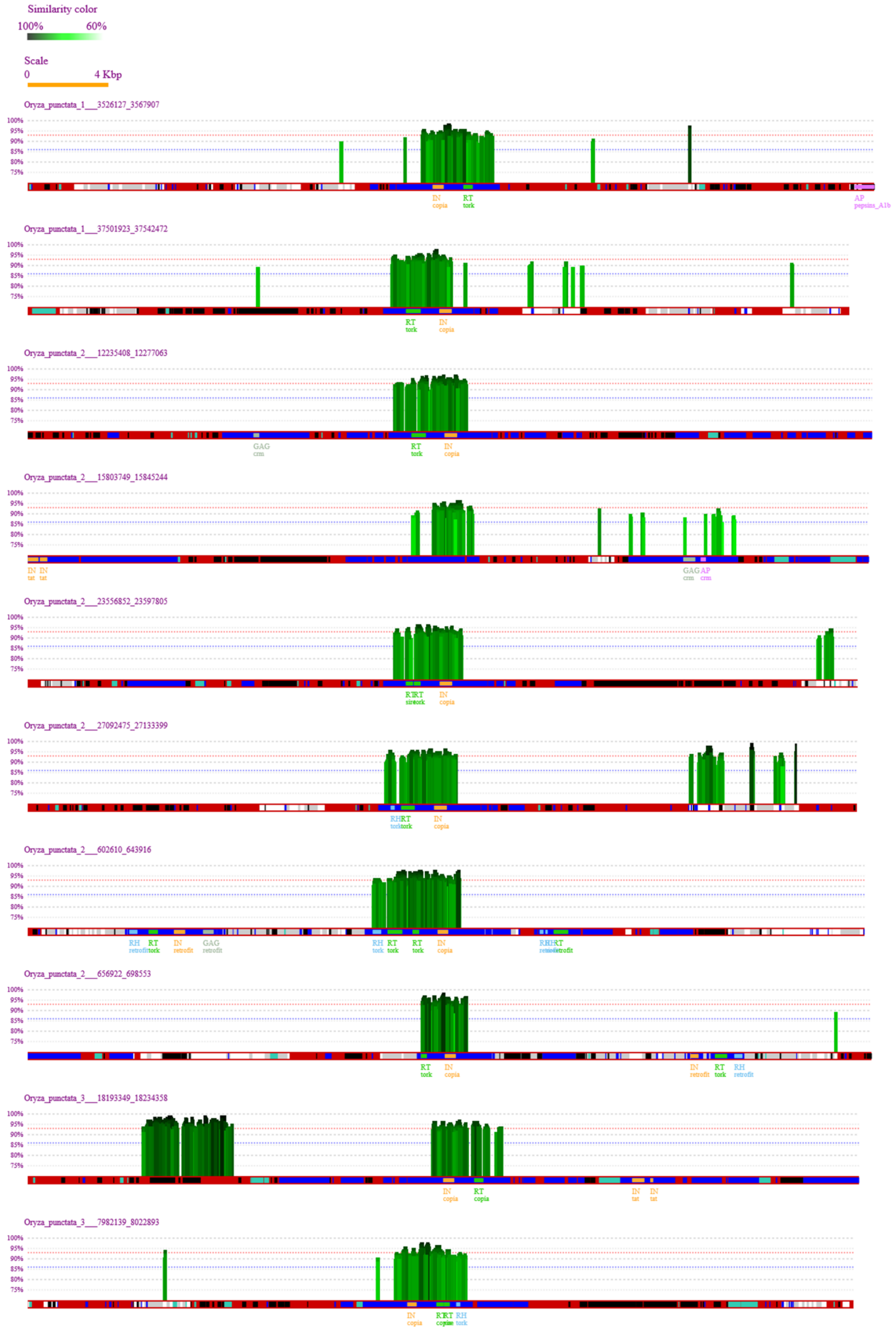

HT_cluster12

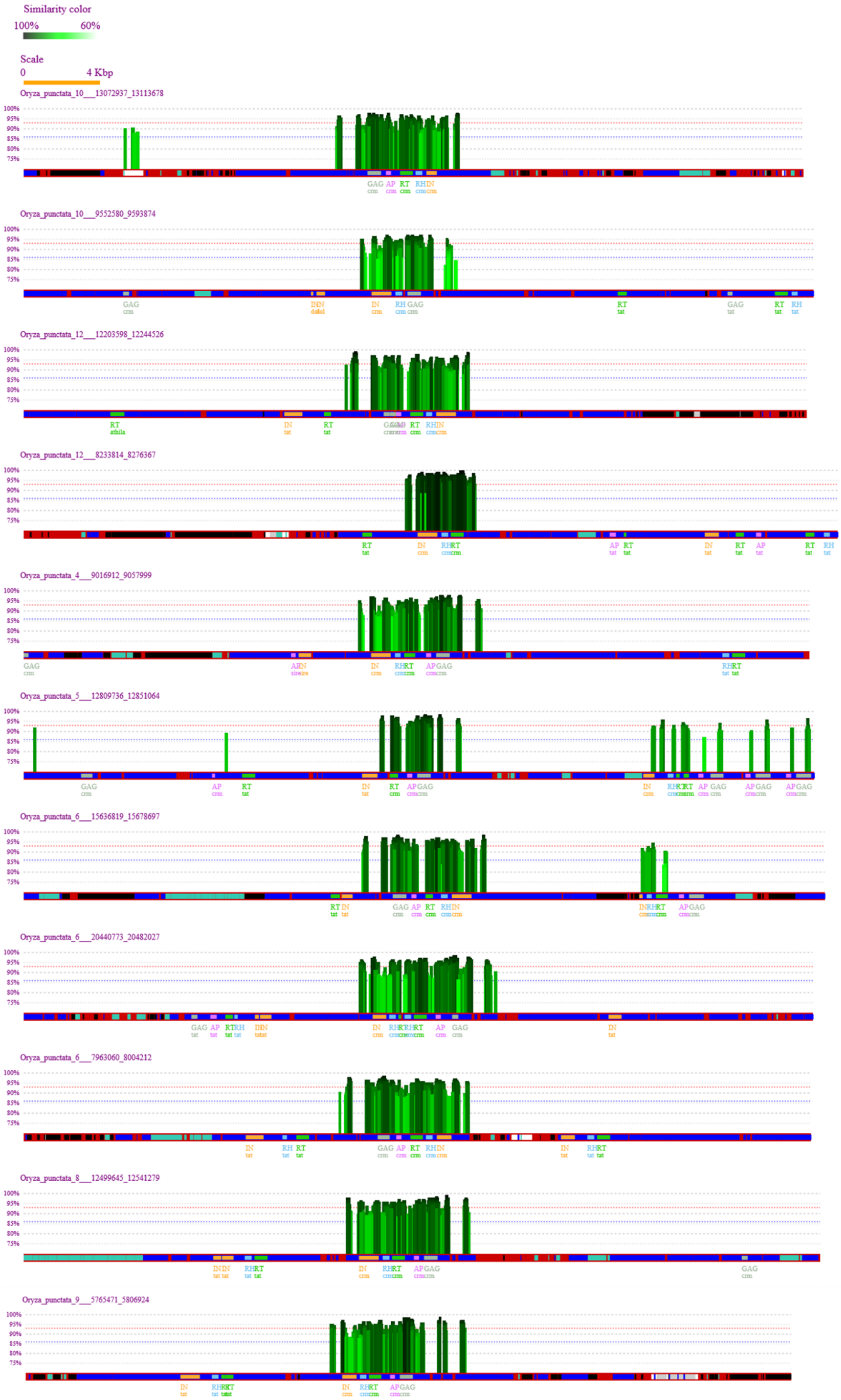

HT_cluster13

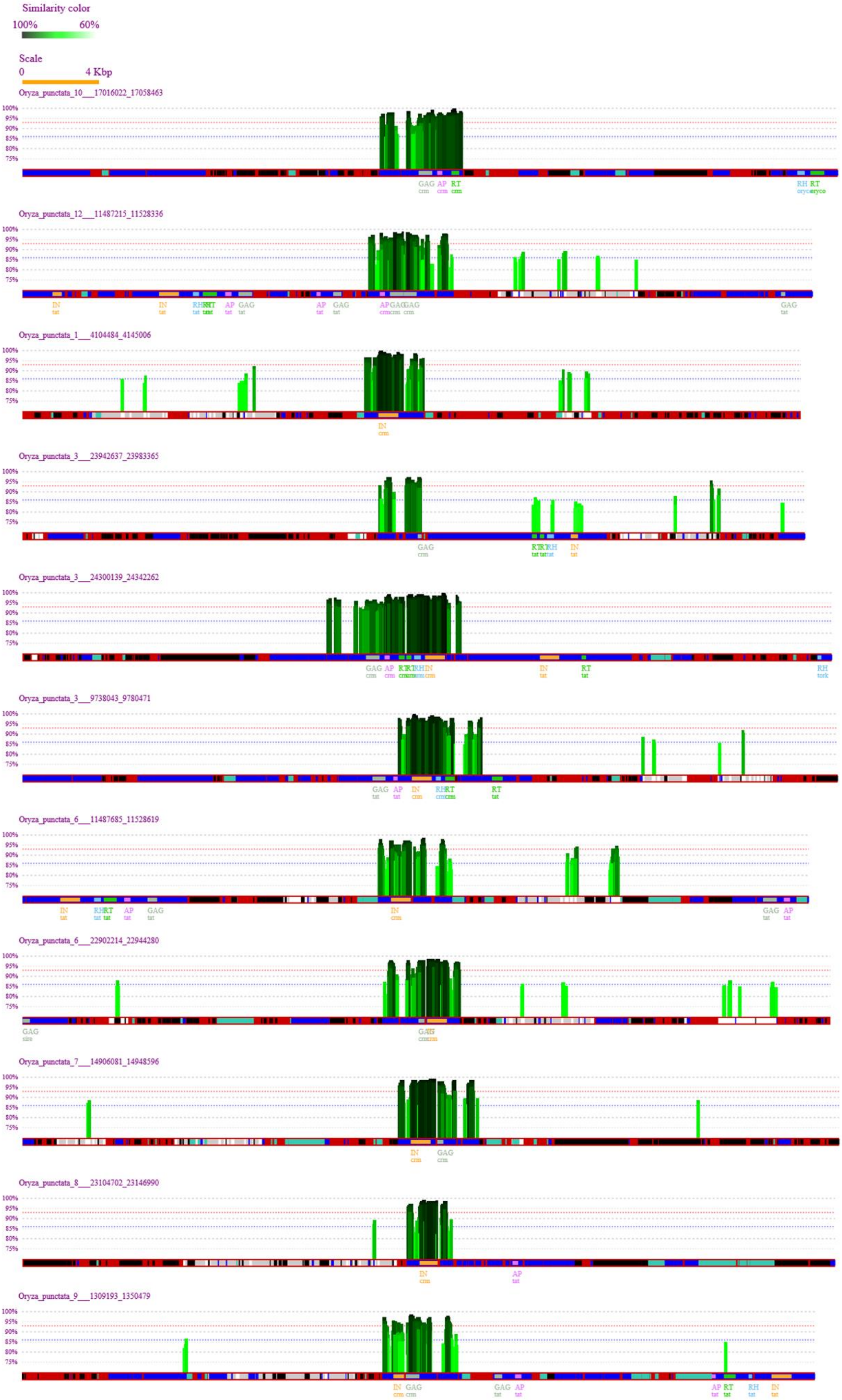

HT_cluster14

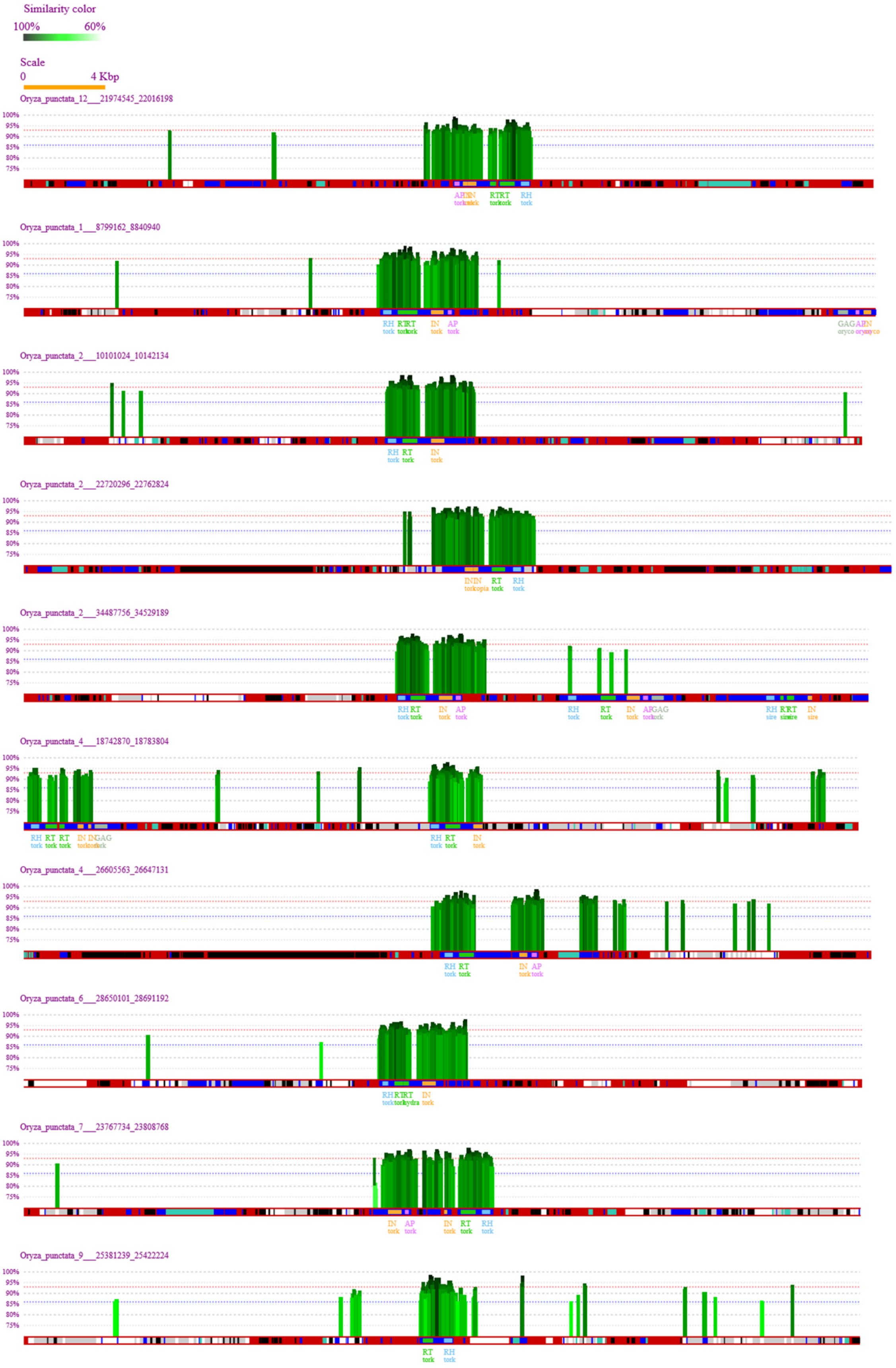

HT_cluster15

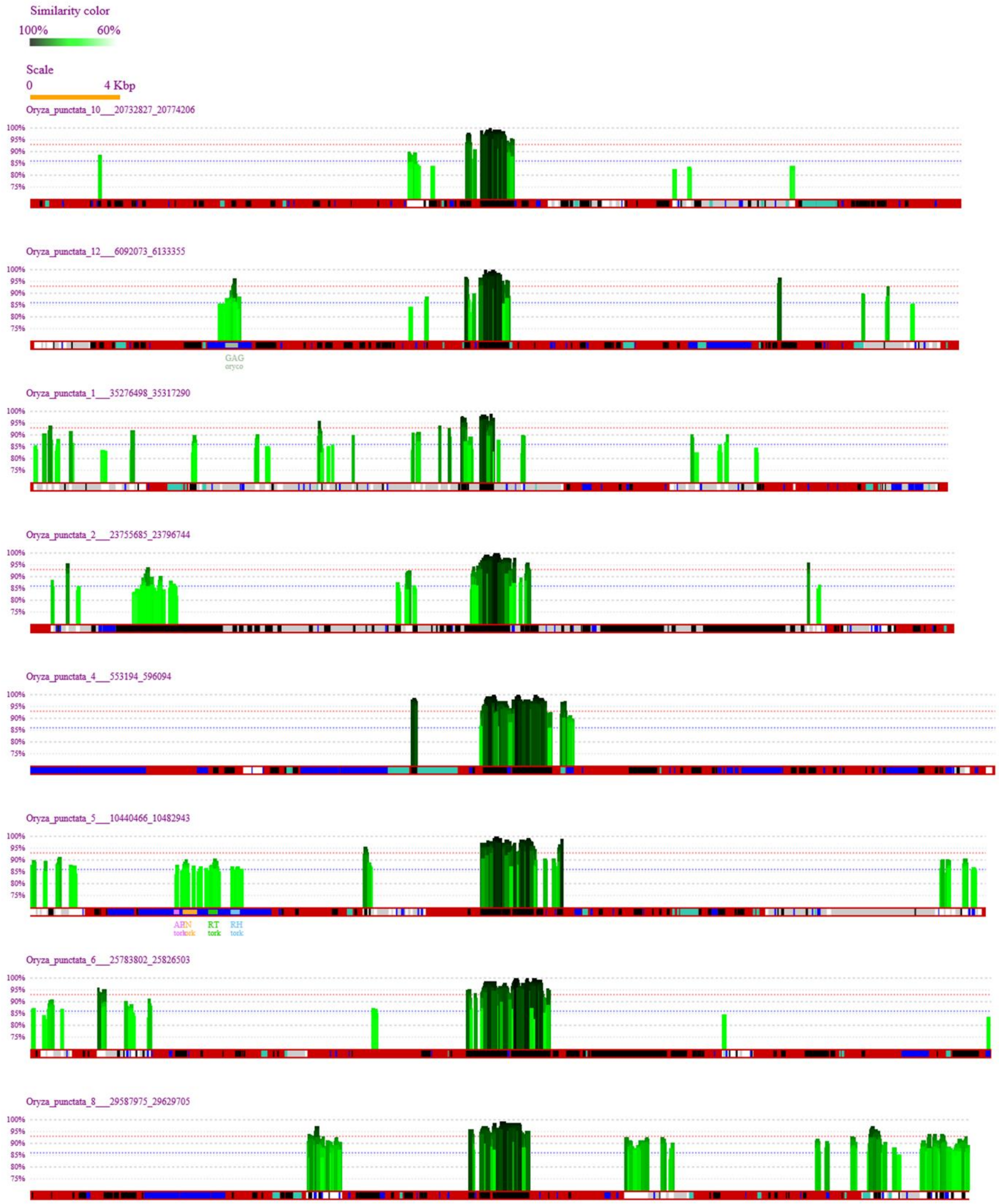

HT_cluster16

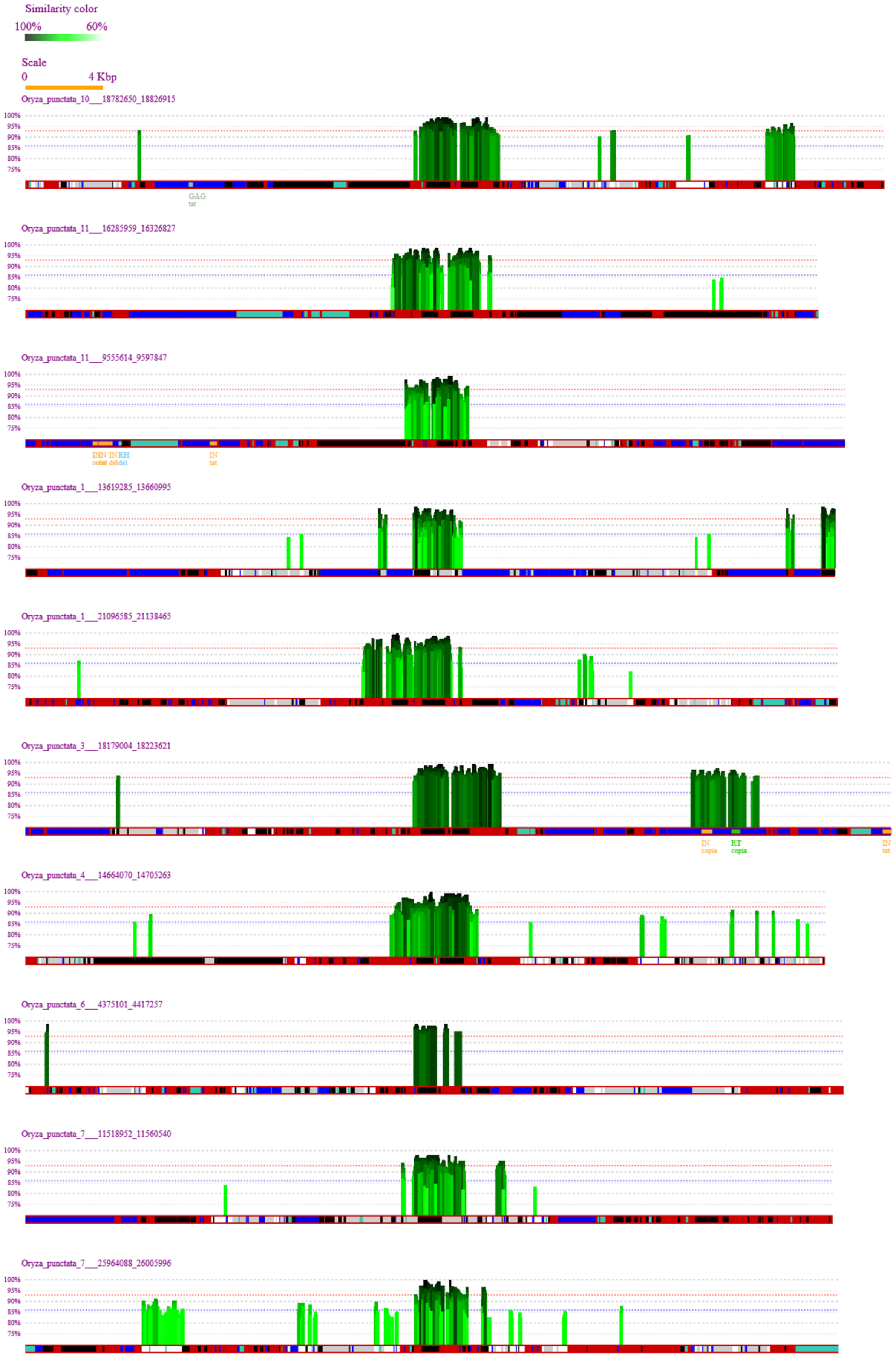

HT_cluster17

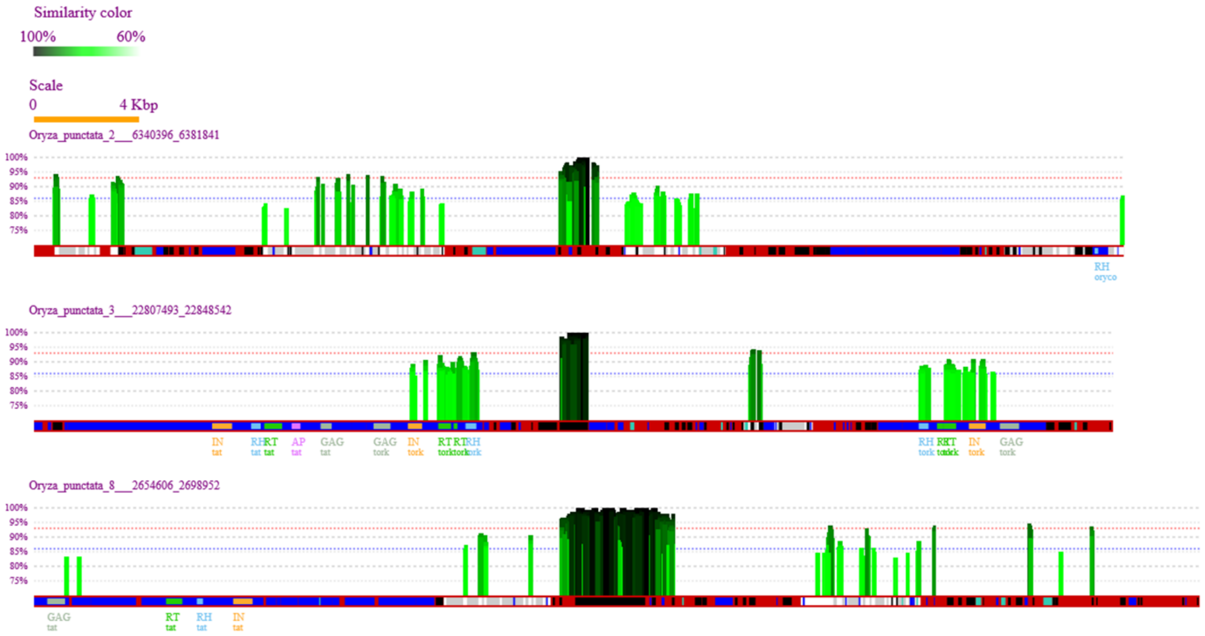

HT_cluster18

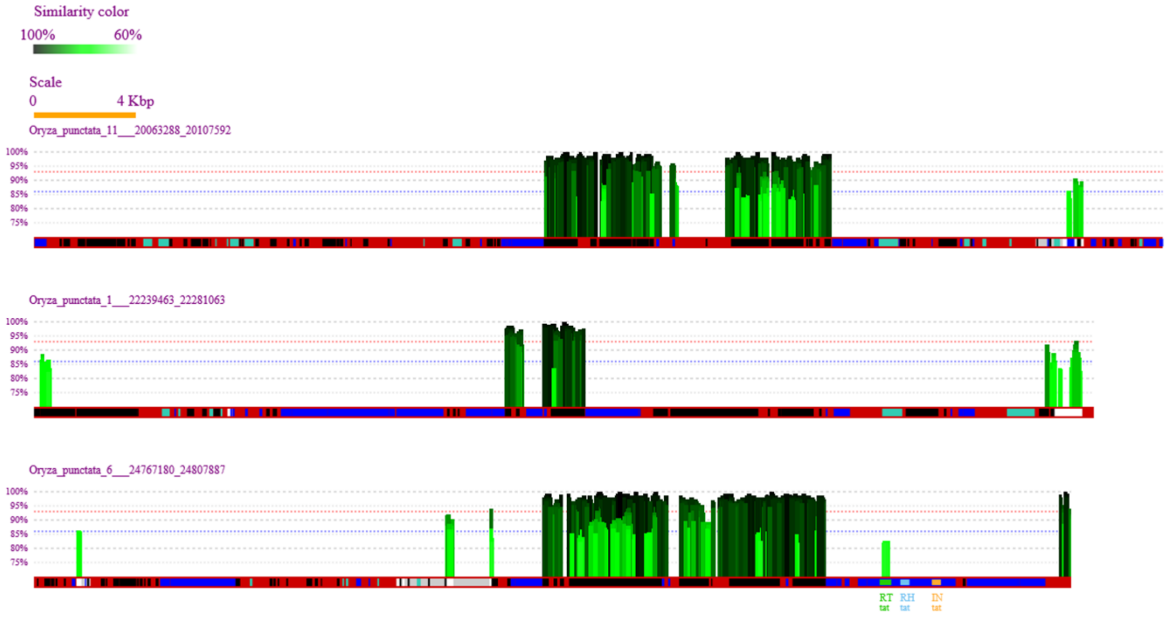

HT_cluster19

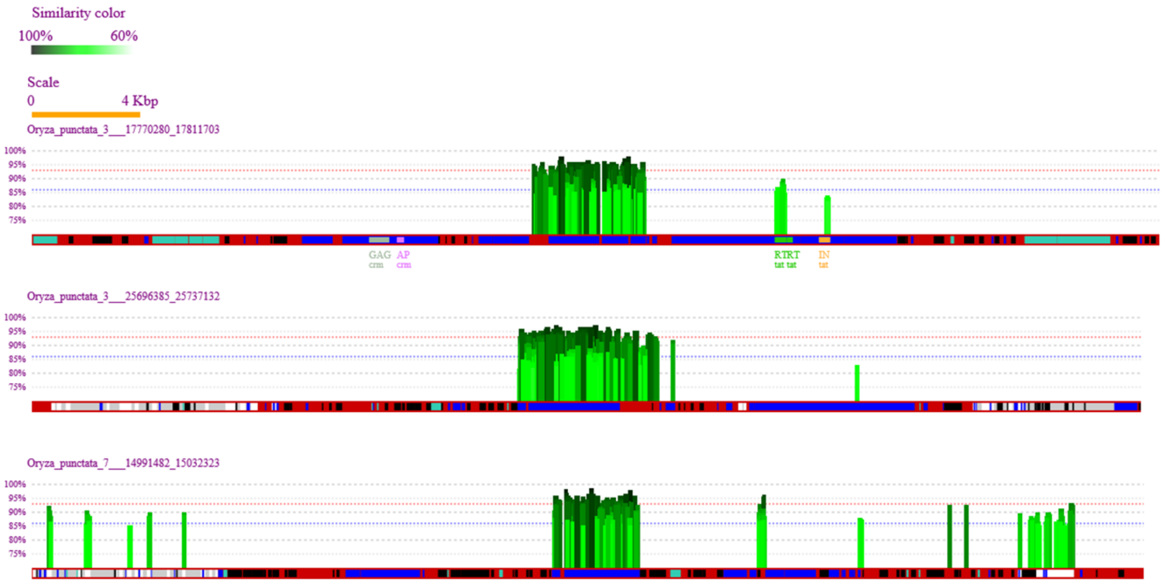

HT_cluster20

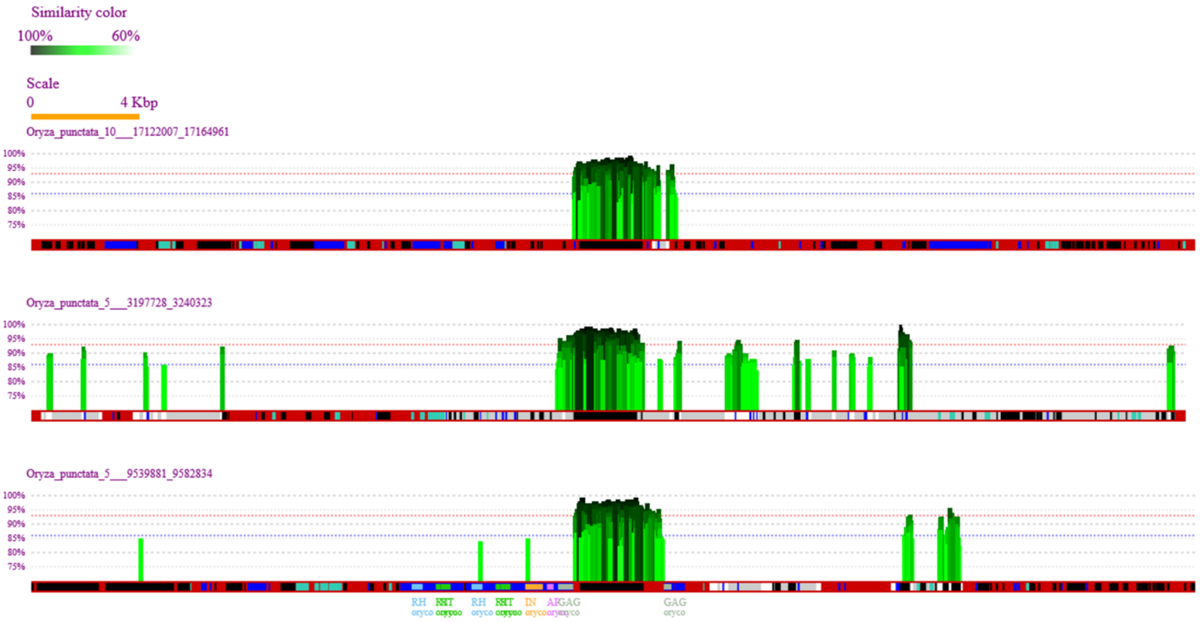

HT_cluster21

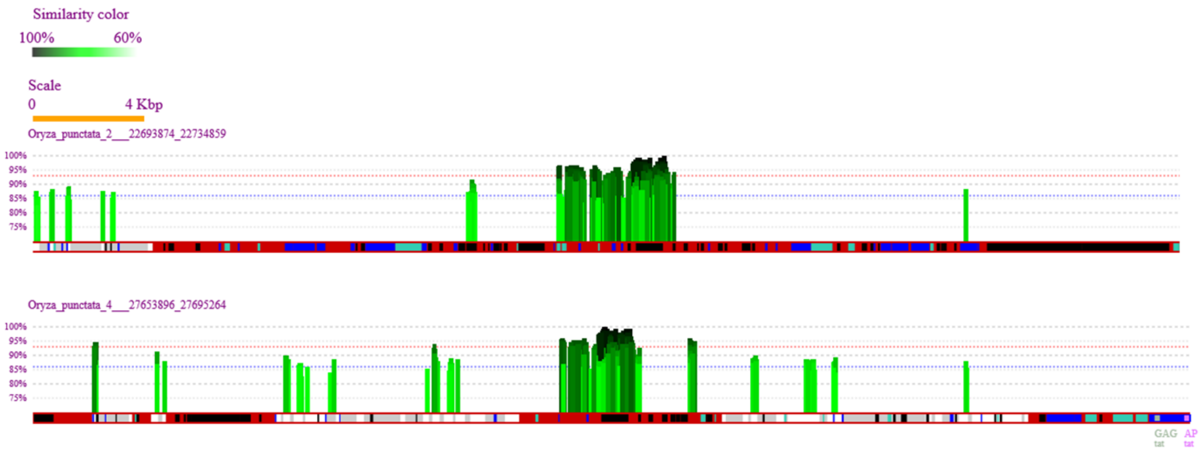

HT_cluster22

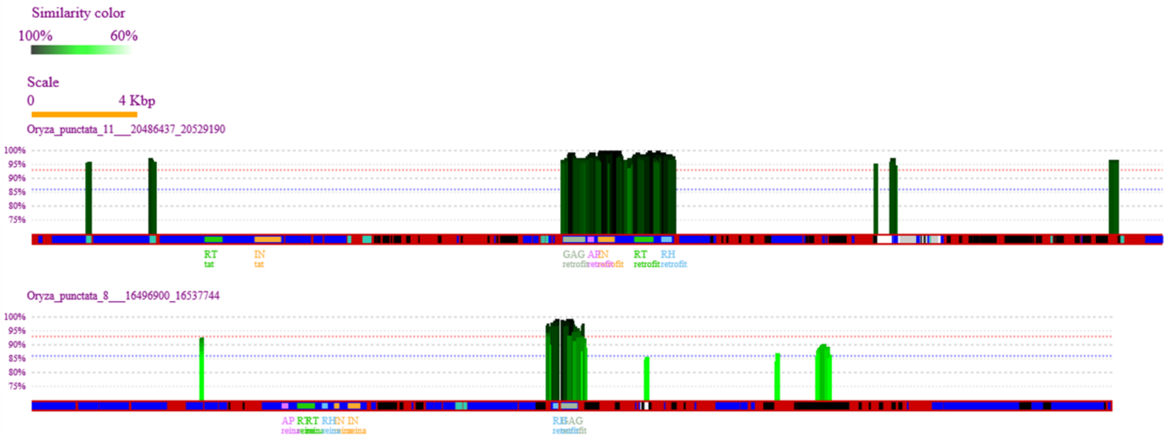

HT_cluster23

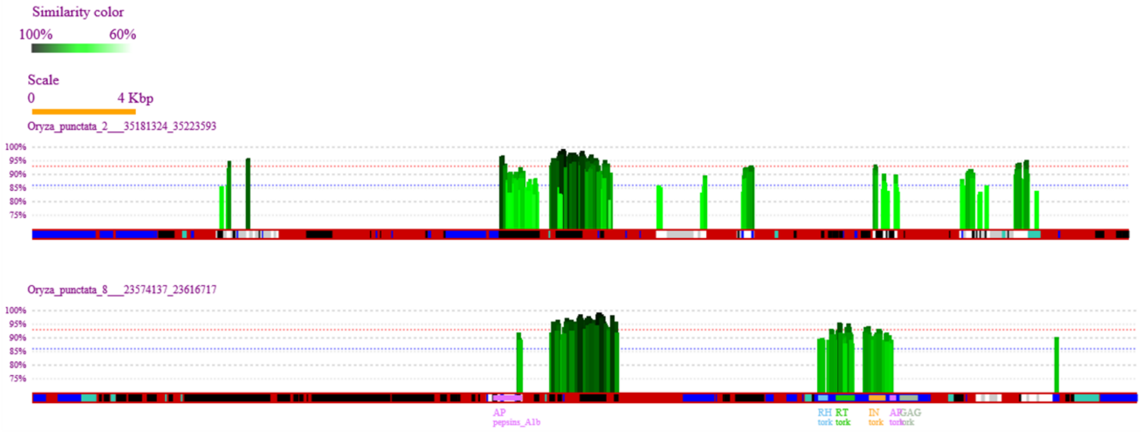

HT_cluster24

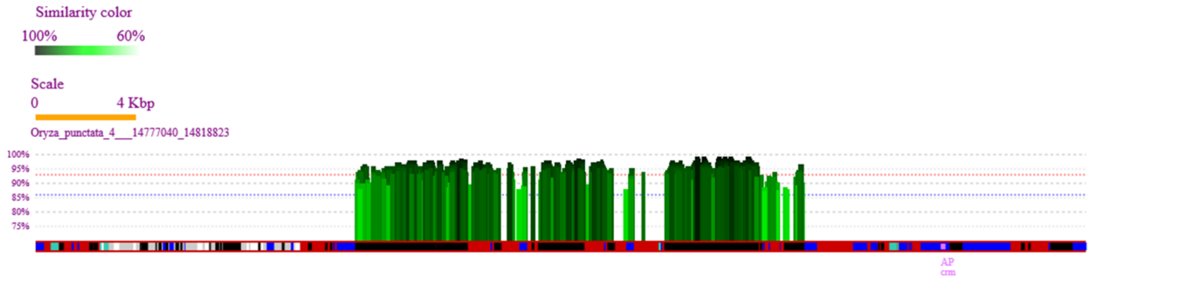

HT_cluster25

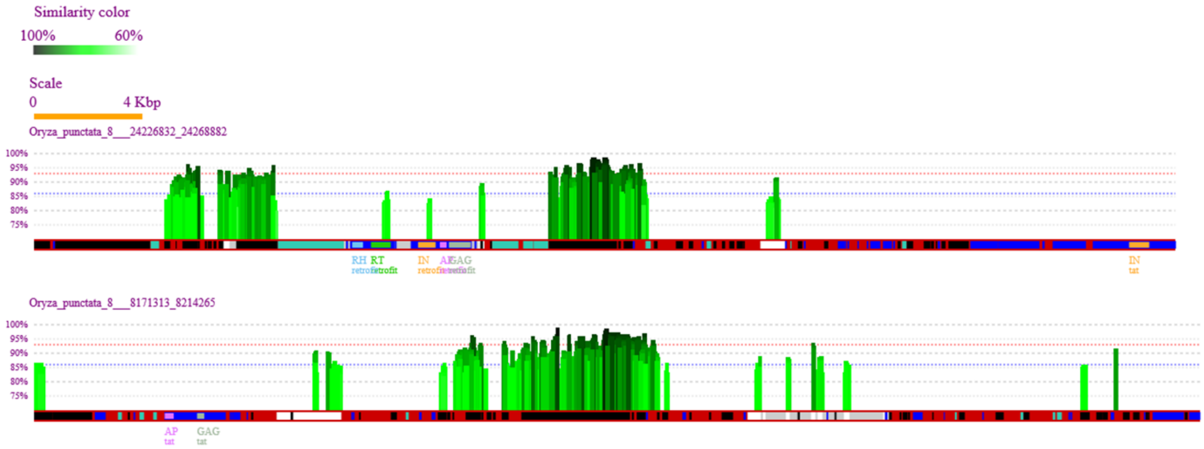

HT_cluster26

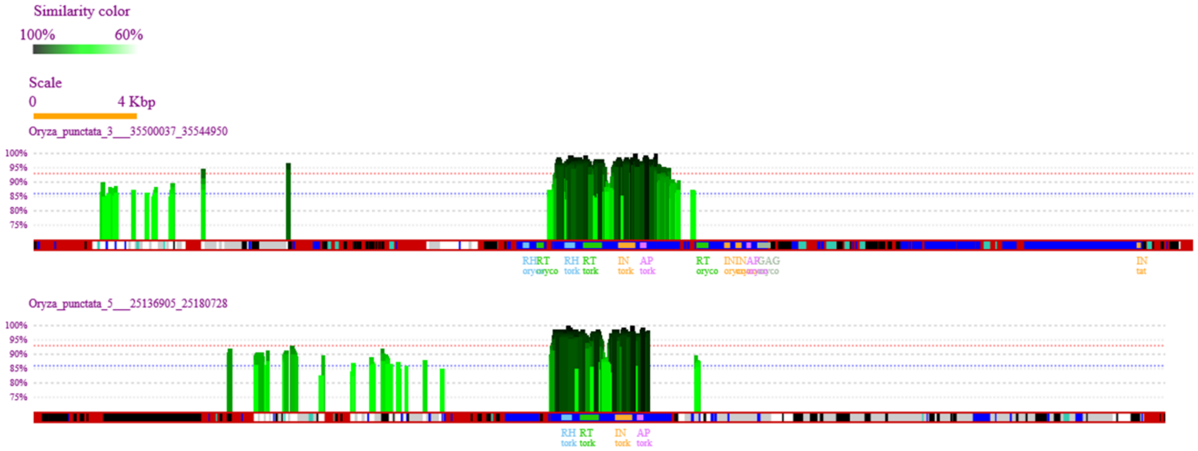

HT_cluster30

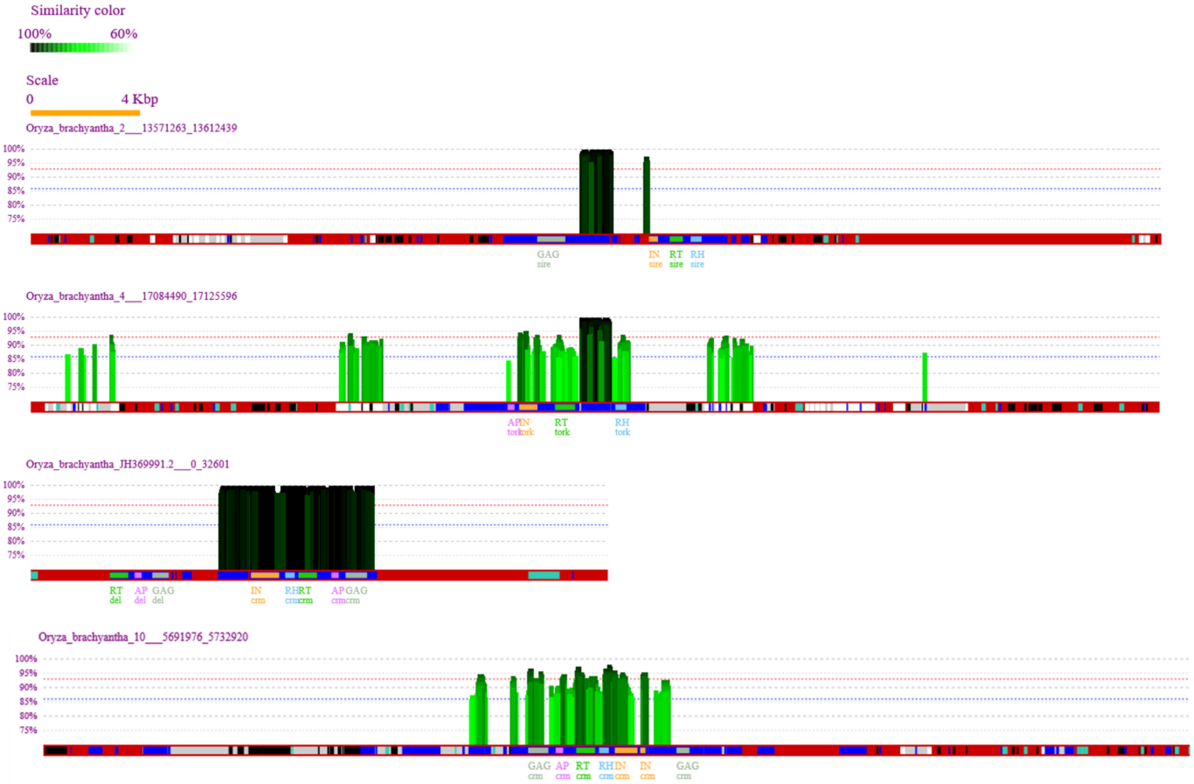

Singlet

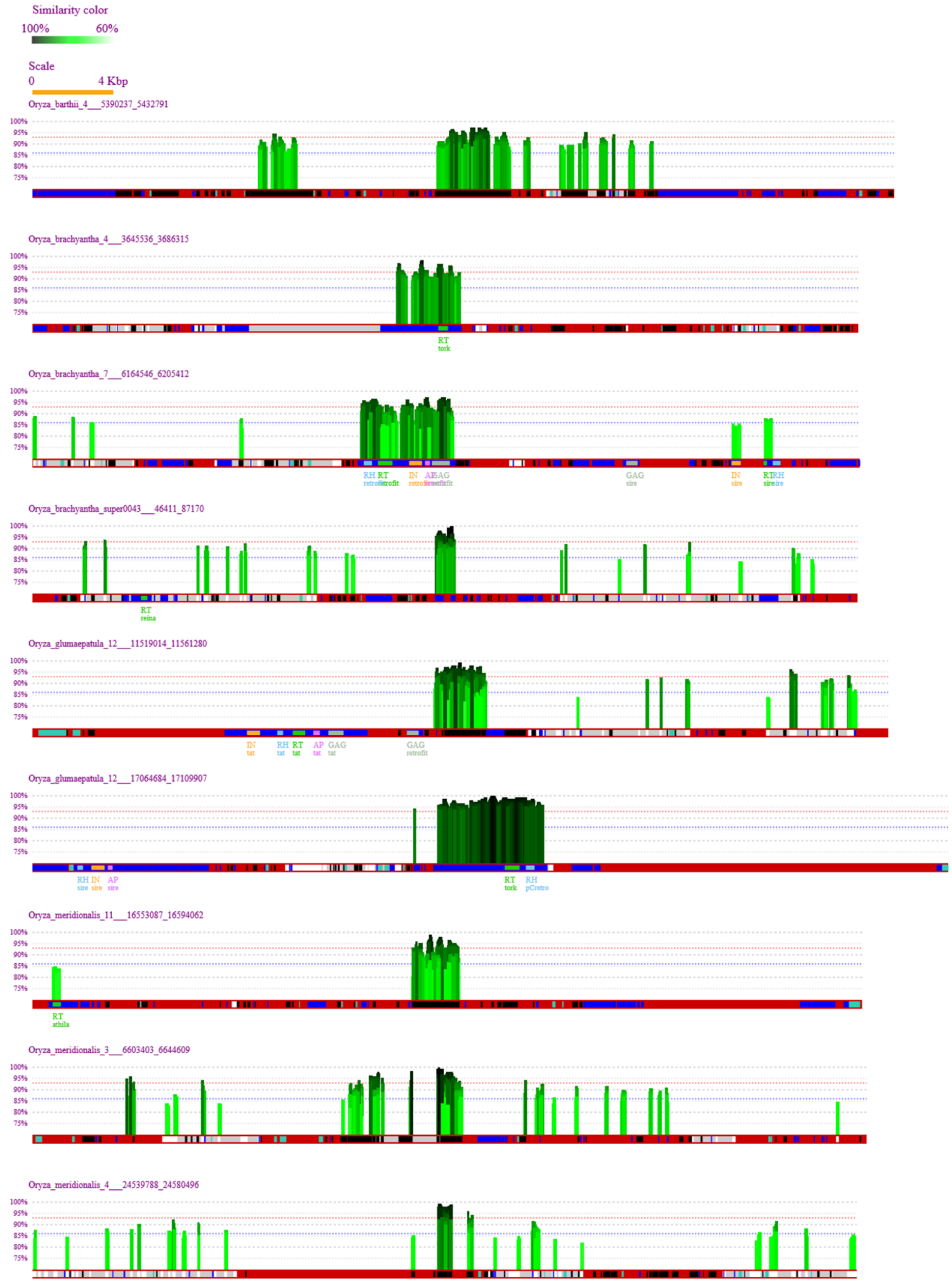

Similarity color
100%
60%
Scale
0
4 Kbp
Oryza_barthii_4___5390237_5432791
Oryza_brachyantha_4___3645536_3686315
Oryza_brachyantha_7___6164546_6205412
Oryza_brachyantha_super0043___46411_87170
Oryza_glumaepatula_12___11519014_11561280
Oryza_glumaepatula_12___17064684_17109907
Oryza_meridionalis_11___16553087_16594062
Oryza_meridionalis_3___6603403_6644609
Oryza_meridionalis_4___24539788_24580496

Singlet

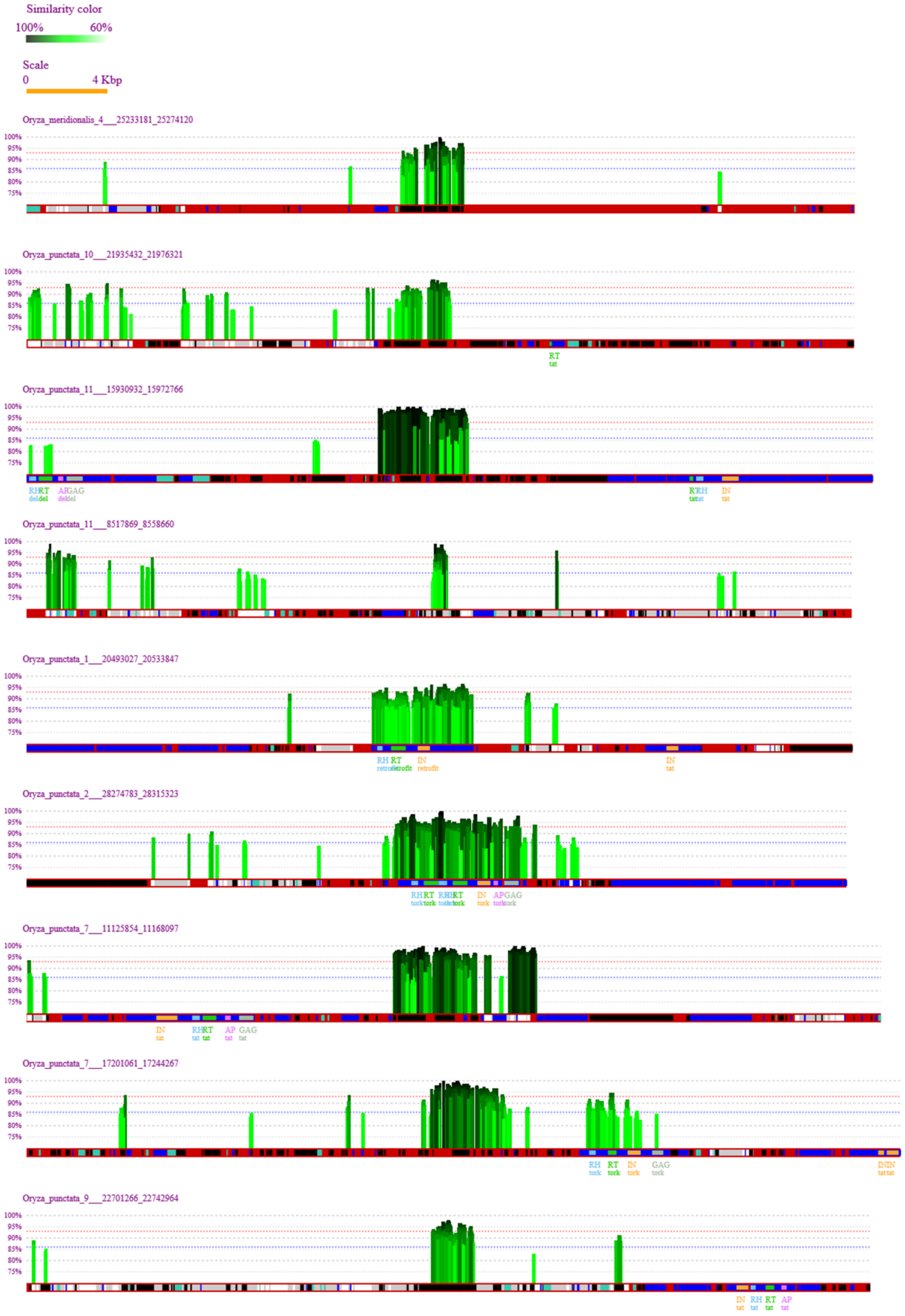
Similarity color
100%
60%
Scale
0
4 Kbp
Oryza_meridionalis_4___25233181_25274120
Oryza_punctata_10___21935432_21976321
Oryza_punctata_11___15930932_15972766
Oryza_punctata_11___8517869_8558660
Oryza_punctata_1___20493027_20533847
Oryza_punctata_2___28274783_28315323
Oryza_punctata_7___11125854_11168097
Oryza_punctata_7___17201061_17244267
Oryza_punctata_9___22701266_22742964
