# Supplementary material for: Sample Sequence Analysis Uncovers Recurrent Horizontal Transfers of Transposable Elements among Grasses
Source: Mol Biol Evol. 2021 May 8;38(9):3664–75. doi: 10.1093/molbev/msab133 (PMC8382918; doi:10.1093/molbev/msab133)
Supplement: msab133_Supplementary_Data [file msab133_supplementary_data.zip › Supplementary_Tables.pdf]

Supplementary Table S1. List and origins of plant samples used in this study.

Species	Source	GenBank Accession
Cenchrus echinatus[a]	B&T World Seeds *86365	SRX8344121
Cenchrus pilosus[a]	AusTRCF Q9142	SRX8344131
Cenchrus setigerus[a]	KEW *0071532	SRX8344132
Cenchrus sieberianus[a]	GRIN PI532675	SRX8344133
Setaria cf. palmifolia	Arusha, Tanzania	SRX8344124
Setaria cf. sulcata	Tanzania (Lundgren and Christin 5; SHD)	SRX8344125
Setaria barbata[a]	GRIN PI862293	SRX8344123
Zuloagaea bulbosa[a]	GRIN PI422481	SRX8344128
Tricholaena monachne[a]	GRIN PI368260	SRX8344126
Melinis ambigua[a]	AusTRCF IBS 594 52263	SRX8344138
Urochloa mosambicensis[a]	AusTRCF CQ 3352	SRX8344127
Eriochloa meyeriana[a]	GRIN PI 364840 01 SD	SRX8344136
Megathyrsus maximus[a]	-	SRX8344122
Echinochloa pyramidalis[a]	GRIN PI 299897	SRX8344130
Echinochloa muricata[a]	GRIN PI 649370	SRX8344129
Echinochloa haploclada[a]	GRIN PI 319195	SRX8344135
Cymbopogon citratus[a]	B&T World Seeds *40533	SRX8344120
Iseilema membranaceum[a]	GRIN PI 240840	SRX8344137
Dichanthium aristatum[a]	GRIN PI 301994 01 SD	SRX8344134

[a] Samples grown by Atkinson *et al*. (2016)

Supplemental Table S2. List of plant genomes used for horizontal transfer investigation.

Species	Family	Citation
Abrus precatorius	Papilionoideae	(Hovde et al. 2019)
Aegilops tauschii	Poaceae	(Jia et al. 2013)
Aethionema arabicum	Brassicaceae	(Haudry et al. 2013)
Amaranthus hypochondriacus	Amaranthaceae	(Clouse et al. 2016)
Amborella trichopoda	Amborellaceae	(Amborella Genome Project 2013)
Ananas comosus	Bromeliaceae	(Ming et al. 2015)
Aquilaria agallocha	Thymelaeaceae	(Chen et al. 2014)
Arabidopsis lyrata	Brassicaceae	(Hu et al. 2011)
Arabidopsis thaliana	Brassicaceae	(The Arabidopsis Genome Initiative 2000)
Arabis alpina	Brassicaceae	(Willing et al. 2015)
Arachis duranensis	Fabaceae	(Bertioli et al. 2016)
Arachis hypogaea	Fabaceae	(Bertioli et al. 2019)
Arachis ipaensis	Fabaceae	(Bertioli et al. 2019)
Asparagus officinalis	Asparagaceae	(Harkess et al. 2017)
Beta vulgaris	Chenopodiaceae	(Dohm et al. 2013)
Brachypodium distachyon	Poaceae	(The International Brachypodium Initiative 2010)
Brassica napus	Brassicaceae	(Chalhoub et al. 2014)
Brassica oleracea	Brassicaceae	(Liu, Liu et al. 2014)
Brassica rapa	Brassicaceae	(Wang et al. 2011)
Camelina sativa	Brassicaceae	(Chaudhary et al. 2020)
Camellia sinensis	Theaceae	(Wei et al. 2018)
Cannabis sativa	Cannabaceae	(van Bakel et al. 2011)
Capsella rubella	Brassicaceae	(Slotte et al. 2013)
Capsicum annuum	Solanaceae	(Kim et al. 2014)
Carica papaya	Caricaceae	(Ming et al. 2008)
Chenopodium quinoa	Amaranthaceae	(Jarvis et al. 2017)
Cicer arietinum	Fabaceae	(Varshney et al. 2013)
Citrus clementina	Rutaceae	(Wu et al. 2014)
Citrus sinensis	Rutaceae	(Xu et al. 2012)
Coffea arabica	Rubiaceae	(Scalabrin et al. 2020)
Conradina grandiflora	Lamiaceae	n/a*
Cucumis melo	Cucurbitaceae	(Garcia-Mas et al. 2012)
Cucumis sativus	Cucurbitaceae	(Huang et al. 2009)
Cucurbita pepo	Cucurbitaceae	(Xanthopoulou et al. 2019)
Cynara cardunculus	Asteraceae	(Scaglione et al. 2016)
Daucus carota	Apiaceae	(Iorizzo et al. 2016)
Dendrobium catenatum	Orchidaceae	(Zhang et al. 2016)
Dianthus caryophyllus	Caryophyllaceae	(Yagi et al. 2014)
Durio zibethinus	Malvaceae	(Teh et al. 2017)
Elaeis guineensis	Arecaceae	(Singh et al. 2013)

Ensete ventricosum	Musaceae	(Harrison et al. 2014)
Erythranthe guttata	Phrymaceae	n/a
Eucalyptus grandis	Myrtaceae	(Myburg et al. 2014)
Eutrema salsugineum	Brassicaceae	(Yang et al. 2013)
Fragaria vesca	Rosaceae	(Shulaev et al. 2011)
Fragaria x ananassa	Rosaceae	(Hirakawa et al. 2014)
Fraxinus excelsior	Oleaceae	(Sollars et al. 2016)
Genlisea aurea	Lentibulariaceae	(Leushkin et al. 2013)
Glycine max	Fabaceae	(Schmutz et al. 2010)
Glycine soja	Fabaceae	(Kim et al. 2010)
Gossypium arboreum	Malvaceae	(Li et al. 2014)
Gossypium hirsutum	Malvaceae	(Huang et al. 2020)
Gossypium raimondii	Malvaceae	(Wang et al. 2012)
Helianthus annuus	Asteraceae	(Badouin et al. 2017)
Herrania umbratica	Malvaceae	n/a
Hevea brasiliensis	Euphorbiaceae	(Rahman et al. 2013)
Ipomoea nil	Convolvulaceae	(Hoshino et al. 2016)
Ipomoea triloba	Convolvulaceae	(Wu et al. 2018)
Jatropha curcas	Euphorbiaceae	(Sato et al. 2011)
Juglans regia	Juglandaceae	(Zhu et al. 2019)
Lactuca sativa	Asteraceae	(Verwaaijen et al. 2018)
Lagenaria siceraria	Cucurbitaceae	(Wu et al. 2017)
Leavenworthia alabamica	Brassicaceae	(Haudry et al. 2013)
Leersia perrieri	Poaceae	n/a
Linum usitatissimum	Linaceae	(Zhiwen et al. 2012)
Lupinus angustifolius	Fabaceae	(K. et al. 2017)
Malus domestica	Rosaceae	(Velasco et al. 2010)
Manihot esculenta	Euphorbiaceae	(Wang, Feng et al. 2014)
Medicago truncatula	Fabaceae	(Young et al. 2011)
Momordica charantia	Cucurbitaceae	(Urasaki et al. 2016)
Morus notabilis	Moraceae	(He et al. 2013)
Musa acuminata	Musaceae	(D'Hont et al. 2012)
Nelumbo nucifera	Nelumbonaceae	(Ming et al. 2013)
Nicotiana attenuata	Solanaceae	(Xu, Brockmöller et al. 2017)
Nicotiana sylvestris	Solanaceae	(Xu, Brockmöller et al. 2017)
Nicotiana tabacum	Solanaceae	(Sierro et al. 2013)
Nicotiana tomentosiformis	Solanaceae	(Sierro et al. 2014)
Nymphaea colorata	Nymphaeaceae	(Zhang et al. 2020)
Olea europaea	Oleaceae	(Cruz et al. 2016)
Oryza sativa	Poaceae	(Goff et al. 2002)
Papaver somniferum	Papaveraceae	(Hu et al. 2018)
Phalaenopsis equestris	Orchidaceae	(Cai et al. 2015)
Phaseolus vulgaris	Fabaceae	(Schmutz et al. 2014)

Phoenix dactylifera	Arecaceae	(Al-Dous et al. 2011)
Pistacia vera	Anacardiaceae	(Zeng et al. 2019)
Populus euphratica	Salicaceae	(Ma et al. 2013)
Populus trichocarpa	Salicaceae	(Tuskan et al. 2006)
Prosopis alba	Fabaceae	(Torales et al. 2013)
Prunus avium	Rosaceae	(Shirasawa et al. 2017)
Prunus mume	Rosaceae	(Zhang et al. 2012)
Prunus persica	Rosaceae	(The International Peach Genome Initiative 2013)
Punica granatum	Lythraceae	(Yuan et al. 2018)
Pyrus bretschneideri	Rosaceae	(Wu et al. 2013)
Quercus lobata	Fagaceae	(Sork et al. 2016)
Quercus suber	Fagaceae	(Ramos et al. 2018)
Raphanus sativus	Brassicaceae	(Kitashiba et al. 2014)
Rhodamnia argentea	Myrtaceae	n/a
Ricinus communis	Euphorbiaceae	(Chan et al. 2010)
Rosa chinensis	Rosaceae	(Hibrand Saint-Oyant et al. 2018)
Sesamum indicum	Pedaliaceae	(Wang, Yu et al. 2014)
Sisymbrium irio	Brassicaceae	(Haudry et al. 2013)
Solanum lycopersicum	Solanaceae	(The Tomato Genome Consortium 2012)
Solanum pennellii	Solanaceae	(Bolger et al. 2014)
Solanum tuberosum	Solanaceae	(Xu et al. 2011)
Spinacia oleracea	Amaranthaceae	(Xu, Jiao *et al*. 2017)
Syzygium oleosum	Myrtaceae	n/a
Tarenaya hassleriana	Cleomaceae	(Cheng et al. 2013)
Theobroma cacao	Malvaceae	(Argout et al. 2010)
Triticum urartu	Poaceae	(Ling et al. 2013)
Vigna angularis	Fabaceae	(Kang et al. 2015)
Vigna radiata	Fabaceae	(Kang et al. 2014)
Vigna unguiculata	Fabaceae	(Lonardi et al. 2019)
Vitis vinifera	Vitaceae	(The French–Italian Public Consortium for Grapevine Genome Characterization 2007)
Zizania latifolia	Poaceae	(Longbiao et al. 2015)
Ziziphus jujuba	Rhamnaceae	(Liu, Zhao et al. 2014)

*n/a: not applicable

Supplementary Table S3. Information about the six additional *Echinochloa* species.

Species	Sequence size (Gb)	Source	GenBank Accession
Echinochloa esculenta	8.0	United States	SRX9503310
Echinochloa crus-galli	7.6	United States	SRX9503311
Echinochloa oryzoides	7.5	Former Soviet Union	SRX9503312
Echinochloa callopus	11.0	Sudan	SRX9503313
Echinochloa colona	8.8	South Africa	SRX9503309
Echinochloa frumentacea	8.9	India	SRX9503314

Supplementary Table S4. Classified TEs from non-targeted HT investigation.

Class (super family)	Family	Number
LTR-retrotransposon (*Gypsy*)	*CRM1*	27
	wihov	9
	pebi	3
LTR-retrotranposon (*Copia*)	*ibulaf*	21
	unknown	20
	dounil	12
	homy	5
	volo	3
	debeh	3
	hani	2
	ijiret	2
	fourf	1
	tiwe	1
DNA transposon	*harbinger*	27
	MuDR	11
	EnSpm	6
	hAT	5
LINE	*L1*	3
Unknown		4
Total		165

Supplementary Table S5. Classification of the singlet horizontally transferred TEs.

Contig name	Class (superfamily)	Family
*O. barthii*_4___5390237_5432791	DNA transposon	*EnSpm*
*O. meridionalis*_3___6603403_6644609	DNA transposon	*Harbinger*
*O. meridionalis*_4___24539788_24580496	DNA transposon	*harbinger*
*O. meridionalis*_4___25233181_25274120	DNA transposon	*harbinger*
*O. punctata*_7___11125854_11168097	DNA transposon	*harbinger*
*O. punctata*_10___21935432_21976321	DNA transposon	*harbinger*
*O. punctata*_11___15930932_15972766	DNA transposon	*harbinger*
*O. punctata*_7___17201061_17244267	DNA transposon	*harbinher*
*O. glumaepatula*_12___11519014_11561280	DNA transposon	*hAT*
*O. punctata*_11___8517869_8558660	DNA transposon	*hAT*
*O. meridionalis*_11___16553087_16594062	DNA transposon	*MuDR*
*O. punctata*_9___22701266_22742964	DNA transposon	*MuDR*
*O. glumaepatula*_12___17064684_17109907	LTR-retrotransposon (*Copia*)	*debeh*
*O. punctata*_2___28274783_28315323	LTR-retrotransposon (*Copia*)	*fourf*
*O. brachyantha*_4___3645536_3686315	LTR-retrotransposon (*Copia*)	*ibulaf*
*O. brachyantha*_7___6164546_6205412	LTR-retrotransposon (*Copia*)	*tiwe*
*O. punctata*_1___20493027_20533847	LTR-retrotransposon (*Copia*)	unknown
*O. brachyantha*_super0043___46411_87170	LTR-retrotransposon (*Gypsy*)	*wihov*

References

Al-Dous EK, George B, Al-Mahmoud ME, Al-Jaber MY, Wang H, Salameh YM, Al-Azwani EK, Chaluvadi S, Pontaroli AC, DeBarry J, et al. 2011. De novo genome sequencing and comparative genomics of date palm (*Phoenix dactylifera*). *Nat Biotechnol*. 29:521-528.

Amborella Genome Project. 2013. The *Amborella* genome and the evolution of flowering plants. *Science* 342:1241089.

Argout X, Salse J, Aury J-M, Guiltinan MJ, Droc G, Gouzy J, Allegre M, Chaparro C, Legavre T, Maximova SN, et al. 2010. The genome of *Theobroma cacao*. *Nat Genet*. 43:101-108.

Badouin H, Gouzy J, Grassa CJ, Murat F, Staton SE, Cottret L, Lelandais-Brière C, Owens GL, Carrère S, Mayjonade B, et al. 2017. The sunflower genome provides insights into oil metabolism, flowering and Asterid evolution. *Nature* 546:148-152.

Bertioli DJ, Cannon SB, Froenicke L, Huang G, Farmer AD, Cannon EKS, Liu X, Gao D, Clevenger J, Dash S, et al. 2016. The genome sequences of *Arachis duranensis* and *Arachis ipaensis*, the diploid ancestors of cultivated peanut. *Nat Genet*. 48:438-446.

Bertioli DJ, Jenkins J, Clevenger J, Dudchenko O, Gao D, Seijo G, Leal-Bertioli SCM, Ren L, Farmer AD, Pandey MK, et al. 2019. The genome sequence of segmental allotetraploid peanut *Arachis hypogaea*. *Nat Genet*. 51: 877-884.

Bolger A, Scossa F, Bolger ME, Lanz C, Maumus F, Tohge T, Quesneville H, Alseekh S, Sørensen I, Lichtenstein G, et al. 2014. The genome of the stress-tolerant wild tomato species *Solanum pennellii*. *Nat Genet*. 46: 1034-1038.

Cai J, Liu X, Vanneste K, Proost S, Tsai W-C, Liu K-W, Chen L-J, He Y, Xu Q, Bian C, et al. 2015. The genome sequence of the orchid *Phalaenopsis equestris*. *Nat Genet*. 47:65-72.

Chalhoub B, Denoeud F, Liu S, Parkin IAP, Tang H, Wang X, Chiquet J, Belcram H, Tong C, Samans B, et al. 2014. Early allopolyploid evolution in the post-Neolithic *Brassica napus* oilseed genome. *Science* 345:950-953.

Chan AP, Crabtree J, Zhao Q, Lorenzi H, Orvis J, Puiu D, Melake-Berhan A, Jones KM, Redman J, Chen G, et al. 2010. Draft genome sequence of the oilseed species *Ricinus communis*. *Nat Biotechnol*. 28: 951-956.

Chaudhary R, Koh CS, Kagale S, Tang L, Wu SW, Lv Z, Mason AS, Sharpe AG, Diederichsen A, Parkin IAP. 2020. Assessing diversity in the *Camelina* genus provides insights into the genome structure of *Camelina sativa*. *G3-Genes Genom Genet*. 10:1297-1308.

Chen C-H, Kuo TC-Y, Yang M-H, Chien T-Y, Chu M-J, Huang L-C, Chen C-Y, Lo H-F, Jeng S-T, Chen L-FO. 2014. Identification of cucurbitacins and assembly of a draft genome for *Aquilaria agallocha*. *BMC Genomics* 15:578.
Cheng S, van den Bergh E, Zeng P, Zhong X, Xu J, Liu X, Hofberger J, de Bruijn S, Bhide AS, Kuelahoglu C, et al. 2013. The *Tarenaya hassleriana* genome provides insight into reproductive trait and genome evolution of crucifers. *Plant Cell* 25:2813-2830.
Clouse JW, Adhikary D, Page JT, Ramaraj T, Deyholos MK, Udall JA, Fairbanks DJ, Jellen EN, Maughan PJ. 2016. The amaranth genome: genome, transcriptome, and physical map assembly. *Plant Genome* 9
Cruz F, Julca I, Gómez-Garrido J, Loska D, Marcet-Houben M, Cano E, Galán B, Frias L, Ribeca P, Derdak S, et al. 2016. Genome sequence of the olive tree, *Olea europaea*. *Giga Science* 5
D'Hont A, Denoeud F, Aury J-M, Baurens F-C, Carreel F, Garsmeur O, Noel B, Bocs S, Droc G, Rouard M, et al. 2012. The banana (*Musa acuminata*) genome and the evolution of monocotyledonous plants. *Nature* 488:213-217.
Dohm JC, Minoche AE, Holtgräwe D, Capella-Gutiérrez S, Zakrzewski F, Tafer H, Rupp O, Sörensen TR, Stracke R, Reinhardt R, et al. 2013. The genome of the recently domesticated crop plant sugar beet (*Beta vulgaris*). *Nature* 505:546-549.
Garcia-Mas J, Benjak A, Sanseverino W, Bourgeois M, Mir G, González VM, Hénaff E, Câmara F, Cozzuto L, Lowy E, et al. 2012. The genome of melon (*Cucumis melo*L.). *Proc Natl Acad Sci U S A*. 109:11872-11877.
Goff SA, Ricke D, Lan T-H, Presting G, Wang R, Dunn M, Glazebrook J, Sessions A, Oeller P, Varma H, et al. 2002. A draft sequence of the rice genome (*Oryza sativa* L. ssp. *japonica*). *Science* 296:92-100.
Harkess A, Zhou J, Xu C, Bowers JE, Van der Hulst R, Ayyampalayam S, Mercati F, Riccardi P, McKain MR, Kakrana A, et al. 2017. The asparagus genome sheds light on the origin and evolution of a young Y chromosome. *Nat Commun*. 8:1279.
Harrison J, Moore K, Paszkiewicz K, Jones T, Grant M, Ambacheew D, Muzemil S, Studholme D. 2014. A draft genome sequence for *Ensete ventricosum*, the drought-rolerant "tree against hunger". *Agronomy* 4:13-33.

Haudry A, Platts AE, Vello E, Hoen DR, Leclercq M, Williamson RJ, Forczek E, Joly-Lopez Z, Steffen JG, Hazzouri KM, et al. 2013. An atlas of over 90,000 conserved noncoding sequences provides insight into crucifer regulatory regions. *Nat Genet*. 45:891-898.
He N, Zhang C, Qi X, Zhao S, Tao Y, Yang G, Lee T-H, Wang X, Cai Q, Li D, et al. 2013. Draft genome sequence of the mulberry tree *Morus notabilis*. *Nat Commun*.4:2445.
Hibrand Saint-Oyant L, Ruttink T, Hamama L, Kirov I, Lakhwani D, Zhou NN, Bourke PM, Daccord N, Leus L, Schulz D, et al. 2018. A high-quality genome sequence of *Rosa chinensis* to elucidate ornamental traits. *Nat Plants* 4:473-484.
Hirakawa H, Shirasawa K, Kosugi S, Tashiro K, Nakayama S, Yamada M, Kohara M, Watanabe A, Kishida Y, Fujishiro T, et al. 2014. Dissection of the octoploid strawberry genome by deep sequencing of the genomes of *Fragaria s*pecies. *DNA Res*. 21:169-181.
Hoshino A, Jayakumar V, Nitasaka E, Toyoda A, Noguchi H, Itoh T, Shin-I T, Minakuchi Y, Koda Y, Nagano AJ, et al. 2016. Genome sequence and analysis of the Japanese morning glory *Ipomoea nil*. *Nat Commun*. 7:13295.
Hovde BT, Daligault HE, Hanschen ER, Kunde YA, Johnson MB, Starkenburg SR, 1 SLJ. 2019. Detection of abrin-like and prepropulchellin-like toxin genes and transcripts using whole genome sequencing and full-length transcript sequencing of *Abrus precatorius. Toxins* 11:691.
Hu TT, Pattyn P, Bakker EG, Cao J, Cheng J-F, Clark RM, Fahlgren N, Fawcett JA, Grimwood J, Gundlach H, et al. 2011. The *Arabidopsis lyrata* genome sequence and the basis of rapid genome size change. *Nat Genet*. 43:476-481.
Hu Y, Zhao R, Xu P, Jiao Y. 2018. The genome of opium poppy reveals evolutionary history of morphinan pathway. *Genom Proteom Bioinf*. 16:460-462.
Huang G, Wu Z, Percy RG, Bai M, Li Y, Frelichowski JE, Hu J, Wang K, Yu JZ, Zhu Y. 2020. Genome sequence of *Gossypium herbaceum* and genome updates of *Gossypium arboreum* and *Gossypium hirsutum* provide insights into cotton A-genome evolution. *Nat Genet*. 52:516-524.
Huang S, Li R, Zhang Z, Li L, Gu X, Fan W, Lucas WJ, Wang X, Xie B, Ni P, et al. 2009. The genome of the cucumber, *Cucumis sativus* L. *Nat Genet*. 41:1275-1281.
Iorizzo M, Ellison S, Senalik D, Zeng P, Satapoomin P, Huang J, Bowman M, Iovene M, Sanseverino W, Cavagnaro P, et al. 2016. A high-quality carrot genome assembly provides new insights into carotenoid accumulation and asterid genome evolution. *Nat Genet*. 48:657-666.

Jarvis DE, Ho YS, Lightfoot DJ, Schmöckel SM, Li B, Borm TJA, Ohyanagi H, Mineta K, Michell CT, Saber N, et al. 2017. The genome of *Chenopodium quinoa*. *Nature* 542:307-312.

Jia J, Zhao S, Kong X, Li Y, Zhao G, He W, Appels R, Pfeifer M, Tao Y, Zhang X, et al. 2013. *Aegilops tauschii* draft genome sequence reveals a gene repertoire for wheat adaptation. *Nature* 496:91-95.

K. HJ, Yao M, G. KL, N. NM, Gagan G, A. AC, E. BP, Armando B, Scott B, Steven C, et al. 2017. A comprehensive draft genome sequence for lupin (*Lupinus angustifolius*), an emerging health food: insights into plant–microbe interactions and legume evolution. *Plant Biotechnol J*. 15:318-330.

Kang YJ, Kim SK, Kim MY, Lestari P, Kim KH, Ha B-K, Jun TH, Hwang WJ, Lee T, Lee J, et al. 2014. Genome sequence of mungbean and insights into evolution within *Vigna* species. *Nat Commun*. 5:5443.

Kang YJ, Satyawan D, Shim S, Lee T, Lee J, Hwang WJ, Kim SK, Lestari P, Laosatit K, Kim KH, et al. 2015. Draft genome sequence of adzuki bean, *Vigna angularis*. *Sci Rep*. 8069

Kim MY, Lee S, Van K, Kim T-H, Jeong S-C, Choi I-Y, Kim D-S, Lee Y-S, Park D, Ma J, et al. 2010. Whole-genome sequencing and intensive analysis of the undomesticated soybean (*Glycine soja* Sieb. and Zucc.) genome. *Proc Natl Acad Sci U S A*. 107:22032-22037.

Kim S, Park M, Yeom S-I, Kim Y-M, Lee JM, Lee H-A, Seo E, Choi J, Cheong K, Kim K-T, et al. 2014. Genome sequence of the hot pepper provides insights into the evolution of pungency in *Capsicum* species. *Nat Genet*. 46:270-278.

Kitashiba H, Li F, Hirakawa H, Kawanabe T, Zou Z, Hasegawa Y, Tonosaki K, Shirasawa S, Fukushima A, Yokoi S, et al. 2014. Draft sequences of the radish (*Raphanus sativus* L.) genome. *DNA Res*. 21:481-490.

Leushkin EV, Sutormin RA, Nabieva ER, Penin AA, Kondrashov AS, Logacheva MD. 2013. The miniature genome of a carnivorous plant *Genlisea aurea* contains a low number of genes and short non-coding sequences. *BMC Genomics* 14:476.

Li F, Fan G, Wang K, Sun F, Yuan Y, Song G, Li Q, Ma Z, Lu C, Zou C, et al. 2014. Genome sequence of the cultivated cotton *Gossypium arboreum*. *Nat Genet*. 46:567-572.

Ling H-Q, Zhao S, Liu D, Wang J, Sun H, Zhang C, Fan H, Li D, Dong L, Tao Y, et al. 2013. Draft genome of the wheat A-genome progenitor *Triticum urartu*. *Nature* 496:87-90.

Liu M-J, Zhao J, Cai Q-L, Liu G-C, Wang J-R, Zhao Z-H, Liu P, Dai L, Yan G, Wang W-J, et al. 2014. The complex jujube genome provides insights into fruit tree biology. *Nat Commun*. 5:5315.

Liu S, Liu Y, Yang X, Tong C, Edwards D, Parkin IAP, Zhao M, Ma J, Yu J, Huang S, et al. 2014. The *Brassica oleracea* genome reveals the asymmetrical evolution of polyploid genomes. *Nat Commun*. 5:3930.

Lonardi S, Muñoz-Amatriaín M, Liang Q, Shu S, Wanamaker SI, Lo S, Tanskanen J, Schulman AH, Zhu T, Luo M-C, et al. 2019. The genome of cowpea (*Vigna unguiculata* [L.] Walp.). *Plant J*. 98:767-782.

Longbiao G, Jie Q, Zujing H, Zihong Y, Chao C, Chuanjun L, Xiufang X, Chu-Yu Y, Ying-Ying W, Hongqing X, et al. 2015. A host plant genome (*Zizania latifolia*) after a century-long endophyte infection. *Plant J*. 83:600-609.

Ma T, Wang J, Zhou G, Yue Z, Hu Q, Chen Y, Liu B, Qiu Q, Wang Z, Zhang J, et al. 2013. Genomic insights into salt adaptation in a desert poplar. *Nat Commun*. 4:2797.

Ming R, Hou S, Feng Y, Yu Q, Dionne-Laporte A, Saw JH, Senin P, Wang W, Ly BV, Lewis KLT, et al. 2008. The draft genome of the transgenic tropical fruit tree papaya (*Carica papaya* Linnaeus). *Nature* 452:991-996.

Ming R, VanBuren R, Liu Y, Yang M, Han Y, Li L-T, Zhang Q, Kim M-J, Schatz MC, Campbell M, et al. 2013. Genome of the long-living sacred lotus (*Nelumbo nucifera* Gaertn.). *Genome Biol*. 14:R41.

Ming R, VanBuren R, Wai CM, Tang H, Schatz MC, Bowers JE, Lyons E, Wang M-L, Chen J, Biggers E, et al. 2015. The pineapple genome and the evolution of CAM photosynthesis. *Nat Genet*. 47:1435-1442.

Myburg AA, Grattapaglia D, Tuskan GA, Hellsten U, Hayes RD, Grimwood J, Jenkins J, Lindquist E, Tice H, Bauer D, et al. 2014. The genome of *Eucalyptus grandis. Nature* 510:356-362.

Rahman AYA, Usharraj AO, Misra BB, Thottathil GP, Jayasekaran K, Feng Y, Hou S, Ong SY, Ng FL, Lee LS, et al. 2013. Draft genome sequence of the rubber tree *Hevea brasiliensis*. *BMC Genomics* 14:75.

Ramos AM, Usié A, Barbosa P, Barros PM, Capote T, Chaves I, Simões F, Abreu I, Carrasquinho I, Faro C, et al. 2018. The draft genome sequence of cork oak. *Sci Data* 5:180069.

Sato S, Hirakawa H, Isobe S, Fukai E, Watanabe A, Kato M, Kawashima K, Minami C, Muraki A, Nakazaki N, et al. 2011. Sequence analysis of the genome of an oil-bearing tree, *Jatropha curcas* L.. *DNA Res*. 18:65-76.

Scaglione D, Reyes-Chin-Wo S, Acquadro A, Froenicke L, Portis E, Beitel C, Tirone M, Mauro R, Lo Monaco A, Mauromicale G, et al. 2016. The genome sequence of the outbreeding globe artichoke constructed *de novo* incorporating a phase-aware low-pass sequencing strategy of F1 progeny. *Sci Rep*. 6:19427.

Scalabrin S, Toniutti L, Di Gaspero G, Scaglione D, Magris G, Vidotto M, Pinosio S, Cattonaro F, Magni F, Jurman I, et al. 2020. A single polyploidization event at the origin of the tetraploid genome of *Coffea arabica* is responsible for the extremely low genetic variation in wild and cultivated germplasm. *Sci Rep*. 10:4642.

Schmutz J, Cannon SB, Schlueter J, Ma J, Mitros T, Nelson W, Hyten DL, Song Q, Thelen JJ, Cheng J, et al. 2010. Genome sequence of the palaeopolyploid soybean. *Nature* 463:178-183.

Schmutz J, McClean PE, Mamidi S, Wu GA, Cannon SB, Grimwood J, Jenkins J, Shu S, Song Q, Chavarro C, et al. 2014. A reference genome for common bean and genome-wide analysis of dual domestications. *Nat Genet*. 46:707-713.

Shirasawa K, Isuzugawa K, Ikenaga M, Saito Y, Yamamoto T, Hirakawa H, Isobe S. 2017. The genome sequence of sweet cherry (*Prunus avium*) for use in genomics-assisted breeding. *DNA Res*. 24:499-508.

Shulaev V, Sargent DJ, Crowhurst RN, Mockler TC, Folkerts O, Delcher AL, Jaiswal P, Mockaitis K, Liston A, Mane SP, et al. 2011. The genome of woodland strawberry (*Fragaria vesca*). *Nat Genet*. 43:109-116.

Sierro N, Battey JN, Ouadi S, Bovet L, Goepfert S, Bakaher N, Peitsch MC, Ivanov NV. 2013. Reference genomes and transcriptomes of *Nicotiana sylvestris* and *Nicotiana tomentosiformis*. *Genome Biol*. 14:R60.

Sierro N, Battey JND, Ouadi S, Bakaher N, Bovet L, Willig A, Goepfert S, Peitsch MC, Ivanov NV. 2014. The tobacco genome sequence and its comparison with those of tomato and potato. *Nat Commun*. 5:3833.

Singh R, Ong-Abdullah M, Low E-TL, Manaf MAA, Rosli R, Nookiah R, Ooi LC-L, Ooi SE, Chan K-L, Halim MA, et al. 2013. Oil palm genome sequence reveals divergence of interfertile species in old and new worlds. *Nature* 500:335-339.

Slotte T, Hazzouri KM, Ågren JA, Koenig D, Maumus F, Guo Y-L, Steige K, Platts AE, Escobar JS, Newman LK, et al. 2013. The *Capsella rubella* genome and the genomic consequences of rapid mating system evolution. *Nat Genet*. 45:831-835.

Sollars ESA, Harper AL, Kelly LJ, Sambles CM, Ramirez-Gonzalez RH, Swarbreck D, Kaithakottil G, Cooper ED, Uauy C, Havlickova L, et al. 2016. Genome sequence and genetic diversity of European ash trees. *Nature* 541: 212-216.

Sork VL, Fitz-Gibbon ST, Puiu D, Crepeau M, Gugger PF, Sherman R, Stevens K, Langley CH, Pellegrini M, Salzberg SL. 2016. First draft assembly and annotation of the genome of a california endemic oak *Quercus lobata* Née (Fagaceae). *G3* 6:3485-3495.

Teh BT, Lim K, Yong CH, Ng CCY, Rao SR, Rajasegaran V, Lim WK, Ong CK, Chan K, Cheng VKY, et al. 2017. The draft genome of tropical fruit durian (*Durio zibethinus*). *Nat Genet*. 49:1633-1641.

The Arabidopsis Genome Initiative. 2000. Analysis of the genome sequence of the flowering plant *Arabidopsis thaliana*. *Nature* 408:796-815.

The French–Italian Public Consortium for Grapevine Genome Characterization. 2007. The grapevine genome sequence suggests ancestral hexaploidization in major angiosperm phyla. *Nature* 449:463-467.

The International Brachypodium Initiative. 2010. Genome sequencing and analysis of the model grass *Brachypodium distachyon*. *Nature* 463:763-768.

The International Peach Genome Initiative. 2013. The high-quality draft genome of peach (*Prunus persica*) identifies unique patterns of genetic diversity, domestication and genome evolution. *Nat Genet*. 45:487-494.

The Tomato Genome Consortium. 2012. The tomato genome sequence provides insights into fleshy fruit evolution. *Nature* 485:635-641.

Torales SL, Rivarola M, Pomponio MF, Gonzalez S, Acuña CV, Fernández P, Lauenstein DL, Verga AR, Hopp HE, Paniego NB, et al. 2013. *De novo* assembly and characterization of leaf transcriptome for the development of functional molecular markers of the extremophile multipurpose tree species *Prosopis alba*. *BMC Genomics* 14:705.

Tuskan GA, DiFazio S, Jansson S, Bohlmann J, Grigoriev I, Hellsten U, Putnam N, Ralph S, Rombauts S, Salamov A, et al. 2006. The genome of black cottonwood, *Populus trichocarpa* (Torr. Gray). *Science* 313:1596-1604.

Urasaki N, Takagi H, Natsume S, Uemura A, Taniai N, Miyagi N, Fukushima M, Suzuki S, Tarora K, Tamaki M, et al. 2016. Draft genome sequence of bitter gourd (*Momordica charantia*), a vegetable and medicinal plant in tropical and subtropical regions. *DNA Res*. 24.

van Bakel H, Stout JM, Cote AG, Tallon CM, Sharpe AG, Hughes TR, Page JE. 2011. The draft genome and transcriptome of *Cannabis sativa*. *Genome Biol*. 12:R102-R102.

Varshney RK, Song C, Saxena RK, Azam S, Yu S, Sharpe AG, Cannon S, Baek J, Rosen BD, Tar'an B, et al. 2013. Draft genome sequence of chickpea (*Cicer arietinum*) provides a resource for trait improvement. *Nat Biotechnol*. 31:240-246.

Velasco R, Zharkikh A, Affourtit J, Dhingra A, Cestaro A, Kalyanaraman A, Fontana P, Bhatnagar SK, Troggio M, Pruss D, et al. 2010. The genome of the domesticated apple (*Malus* × *domestica* Borkh.). *Nat Genet*. 42:833-839.

Verwaaijen B, Wibberg D, Nelkner J, Gordin M, Rupp O, Winkler A, Bremges A, Blom J, Grosch R, Pühler A, et al. 2018. Assembly of the *Lactuca sativa*, L. cv. Tizian draft genome sequence reveals differences within major resistance complex 1 as compared to the cv. Salinas reference genome. *J Biotechnol*. 267:12-18.

Wang K, Wang Z, Li F, Ye W, Wang J, Song G, Yue Z, Cong L, Shang H, Zhu S, et al. 2012. The draft genome of a diploid cotton *Gossypium raimondii*. *Nat Genet*. 44:1098-1103.

Wang L, Yu S, Tong C, Zhao Y, Liu Y, Song C, Zhang Y, Zhang X, Wang Y, Hua W, et al. 2014. Genome sequencing of the high oil crop sesame provides insight into oil biosynthesis. *Genome Biol*.15:R39.

Wang W, Feng B, Xiao J, Xia Z, Zhou X, Li P, Zhang W, Wang Y, Møller BL, Zhang P, et al. 2014. Cassava genome from a wild ancestor to cultivated varieties. *Nat Commun*. 5:5110.

Wang X, Wang H, Wang J, Sun R, Wu J, Liu S, Bai Y, Mun J-H, Bancroft I, Cheng F, et al. 2011. The genome of the mesopolyploid crop species *Brassica rapa*. *Nat Genet*. 43:1035-1039.

Wei C, Yang H, Wang S, Zhao J, Liu C, Gao L, Xia E, Lu Y, Tai Y, She G, et al. 2018. Draft genome sequence of *Camellia sinensis* var. *sinensis* provides insights into the evolution of the tea genome and tea quality. *Proc Natl Acad Sci U S A*. 115:E4151.

Willing E-M, Rawat V, Mandáková T, Maumus F, James GV, Nordström KJV, Becker C, Warthmann N, Chica C, Szarzynska B, et al. 2015. Genome expansion of *Arabis alpina* linked with retrotransposition and reduced symmetric DNA methylation. *Nat Plants* 1:14023.

Wu GA, Prochnik S, Jenkins J, Salse J, Hellsten U, Murat F, Perrier X, Ruiz M, Scalabrin S, Terol J, et al. 2014. Sequencing of diverse mandarin, pummelo and orange genomes reveals complex history of admixture during citrus domestication. *Nat Biotechnol*. 32:656-662.
Wu J, Wang Z, Shi Z, Zhang S, Ming R, Zhu S, Khan MA, Tao S, Korban SS, Wang H, et al. 2013. The genome of the pear (*Pyrus bretschneideri* Rehd.). *Genome Res*. 23:396-408.
Wu S, Lau KH, Cao Q, Hamilton JP, Sun H, Zhou C, Eserman L, Gemenet DC, Olukolu BA, Wang H, et al. 2018. Genome sequences of two diploid wild relatives of cultivated sweetpotato reveal targets for genetic improvement. *Nat Commun*. 9:4580.
Wu S, Shamimuzzaman M, Sun H, Salse J, Sui X, Wilder A, Wu Z, Levi A, Xu Y, Ling K-S, et al. 2017. The bottle gourd genome provides insights into Cucurbitaceae evolution and facilitates mapping of a *Papaya ring-spot virus* resistance locus. *Plant J*. 92:963-975.
Xanthopoulou A, Montero-Pau J, Mellidou I, Kissoudis C, Blanca J, Picó B, Tsaballa A, Tsaliki E, Dalakouras A, Paris HS, et al. 2019. Whole-genome resequencing of *Cucurbita pepo* morphotypes to discover genomic variants associated with morphology and horticulturally valuable traits. *Hortic Res*. 6:94.
Xu C, Jiao C, Sun H, Cai X, Wang X, Ge C, Zheng Y, Liu W, Sun X, Xu Y, et al. 2017. Draft genome of spinach and transcriptome diversity of 120 Spinacia accessions. *Nat Commun*. 8:15275.
Xu Q, Chen L-L, Ruan X, Chen D, Zhu A, Chen C, Bertrand D, Jiao W-B, Hao B-H, Lyon MP, et al. 2012. The draft genome of sweet orange (*Citrus sinensis*). *Nat Genet*. 45:59-66.
Xu S, Brockmöller T, Navarro-Quezada A, Kuhl H, Gase K, Ling Z, Zhou W, Kreitzer C, Stanke M, Tang H, et al. 2017. Wild tobacco genomes reveal the evolution of nicotine biosynthesis. *Proc Natl Acad Sci U S A*. 114:6133-6138.
Xu X, Pan S, Cheng S, Zhang B, Mu D, Ni P, Zhang G, Yang S, Li R, Wang J, et al. 2011. Genome sequence and analysis of the tuber crop potato. *Nature* 475:189-195.
Yagi M, Kosugi S, Hirakawa H, Ohmiya A, Tanase K, Harada T, Kishimoto K, Nakayama M, Ichimura K, Onozaki T, et al. 2014. Sequence analysis of the genome of carnation (*Dianthus caryophyllus* L.). *DNA Res*. 21:231-241.
Yang R, Jarvis DE, Chen H, Beilstein MA, Grimwood J, Jenkins J, Shu S, Prochnik S, Xin M, Ma C, et al. 2013. The reference genome of the halophytic plant *Eutrema salsugineum*. *Front Plant Sci*. 4:46.

Young ND, Debellé F, Oldroyd GED, Geurts R, Cannon SB, Udvardi MK, Benedito VA, Mayer KFX, Gouzy J, Schoof H, et al. 2011. The medicago genome provides insight into the evolution of rhizobial symbioses. Nature 480:520-524.

Yuan Z, Fang Y, Zhang T, Fei Z, Han F, Liu C, Liu M, Xiao W, Zhang W, Wu S, et al. 2018. The pomegranate (*Punica granatum* L.) genome provides insights into fruit quality and ovule developmental biology. *Plant Biotechnol J*. 16:1363-1374.

Zeng L, Tu X-L, Dai H, Han F-M, Lu B-S, Wang M-S, Nanaei HA, Tajabadipour A, Mansouri M, Li X-L, et al. 2019. Whole genomes and transcriptomes reveal adaptation and domestication of pistachio. *Genome Biol*. 20:79.

Zhang G-Q, Xu Q, Bian C, Tsai W-C, Yeh C-M, Liu K-W, Yoshida K, Zhang L-S, Chang S-B, Chen F, et al. 2016. The *Dendrobium catenatum* Lindl. genome sequence provides insights into polysaccharide synthase, floral development and adaptive evolution. *Sci Rep*. 6:19029.

Zhang L, Chen F, Zhang X, Li Z, Zhao Y, Lohaus R, Chang X, Dong W, Ho SYW, Liu X, et al. 2020. The water lily genome and the early evolution of flowering plants. Nature 577:79-84.

Zhang Q, Chen W, Sun L, Zhao F, Huang B, Yang W, Tao Y, Wang J, Yuan Z, Fan G, et al. 2012. The genome of *Prunus mume*. *Nat Commun*. 3:1318.

Zhiwen W, Neil H, Leonardo G, Shilin Z, Daihu S, Joshua M, Linfeng Y, Simon H, Godfrey N, Raju D, et al. 2012. The genome of flax (*Linum usitatissimum*) assembled de novo from short shotgun sequence reads. *Plant J*. 72:461-473.

Zhu T, Wang L, You FM, Rodriguez JC, Deal KR, Chen L, Li J, Chakraborty S, Balan B, Jiang C-Z, et al. 2019. Sequencing a *Juglans regia* × *J. microcarpa* hybrid yields high-quality genome assemblies of parental species. *Hortic Res*. 6:55.
